# Evidence that genes involved in hedgehog signaling are associated with both bipolar disorder and high BMI

**DOI:** 10.1038/s41398-019-0652-x

**Published:** 2019-11-21

**Authors:** Claudia Pisanu, Michael J. Williams, Diana M. Ciuculete, Gaia Olivo, Maria Del Zompo, Alessio Squassina, Helgi B. Schiöth

**Affiliations:** 1 Unit of Functional Pharmacology, Department of Neuroscience, Uppsala University, Uppsala, Sweden; 2 Department of Biomedical Sciences, Section of Neuroscience and Clinical Pharmacology, University of Cagliari, Cagliari, Italy; 3 Institute for Translational Medicine and Biotechnology, Sechenov First Moscow State Medical University, Moscow, Russia

**Keywords:** Clinical genetics, Bipolar disorder

## Abstract

Patients with bipolar disorder (BD) show higher frequency of obesity and type 2 diabetes (T2D), but the underlying genetic determinants and molecular pathways are not well studied. Using large publicly available datasets, we (1) conducted a gene-based analysis using MAGMA to identify genes associated with BD and body mass index (BMI) or T2D and investigated their functional enrichment; and (2) performed two meta-analyses between BD and BMI, as well as BD and T2D using Metasoft. Target druggability was assessed using the Drug Gene Interaction Database (DGIdb). We identified 518 and 390 genes significantly associated with BD and BMI or BD and T2D, respectively. A total of 52 and 12 genes, respectively, were significant after multiple testing correction. Pathway analyses conducted on nominally significant targets showed that genes associated with BD and BMI were enriched for the Neuronal cell body Gene Ontology (GO) term (*p* = 1.0E−04; false discovery rate (FDR) = 0.025) and different pathways, including the Signaling by Hedgehog pathway (*p* = 4.8E−05, FDR = 0.02), while genes associated with BD and T2D showed no specific enrichment. The meta-analysis between BD and BMI identified 64 relevant single nucleotide polymorphisms (SNPs). While the majority of these were located in intergenic regions or in a locus on chromosome 16 near and in the *NPIPL1* and *SH2B1* genes (best SNP: rs4788101, *p* = 2.1E−24), five were located in the *ETV5* gene (best SNP: rs1516725, *p* = 1E−24), which was previously associated with both BD and obesity, and one in the *RPGRIP1L* gene (rs1477199, *p* = 5.7E−09), which was also included in the Signaling by Hedgehog pathway. The meta-analysis between BD and T2D identified six significant SNPs, three of which were located in *ALAS1* (best SNP: rs352165, *p* = 3.4E−08). Thirteen SNPs associated with BD and BMI, and one with BD and T2D, were located in genes which are part of the druggable genome. Our results support the hypothesis of shared genetic determinants between BD and BMI and point to genes involved in Hedgehog signaling as promising targets.

## Introduction

Bipolar disorder (BD) is a chronic and disabling psychiatric illness, with a prevalence of 0.8–1.2% in the general population^1^, characterized by recurrent manic and depressive episodes. Being characterized by high rates of physical comorbidity and increased risk for suicide, BD is associated with decreased life expectancy and increased all-cause mortality, and therefore is a major socio-economic burden^2^.

Patients with BD show increased frequency of overweight and obesity (41% compared to 27% in general population in US)^3,4^. Additionally, patients with BD show a three-fold higher risk of type 2 diabetes (T2D)^5,6^ compared to the general population. These two conditions greatly contribute to a higher risk of cardiovascular diseases, which represent the leading cause for increased mortality in bipolar patients^7^. Indeed, obesity exerts a negative impact on the course of BD, as this comorbidity is associated with higher episode frequency^8^, rates of disability^9^, suicide attempts^10^, psychiatric and medical comorbidities^11^ as well as cognitive impairment^12^ and white matter abnormalities^13,14^. Conversely, a lower body mass index (BMI) has been associated with positive response to lithium treatment^9^, which represents the first line of treatment in BD, being effective in preventing relapses of both polarities^15^. These results suggest a potential interplay between BMI and molecular targets involved in the mechanism of action of this mood stabilizer. Although a link between BD and BMI has been established, the molecular mechanisms underlying this comorbidity have only recently started to be investigated and are still largely unknown^16,17^. Increasing our scarce knowledge of the factors predisposing patients with BD to develop metabolic comorbidities such as obesity and T2D would be of great importance, eventually allowing us to identify patients that could benefit from early monitoring and/or intervention.

Although lifestyle factors and adverse effects of pharmacotherapy play an important role in the increased susceptibility of BD patients to metabolic comorbidities^5^, a higher BMI has also been reported in adolescents and drug naïve patients^18,19^, thus suggesting the existence of common pathophysiological processes, as well as genetic/epigenetic links underlying these conditions^5,20,21^. Indeed, BD, obesity and T2D are multifactorial disorders that show a high heritability, estimated at 60–80% in BD^22^, 40–70% in obesity^23,24^ and 30–70% in T2D^25^. In a recent study, it was shown that *Ets96B* (the *ETV5* homolog in *Drosophila melanogaster*) regulates a number of genes involved in neuroprotection and that its inhibition induces BD- and obesity-related phenotypes^20^. Moreover, single nucleotide polymorphisms (SNPs) located in the human *ETV5* gene were associated with BD^20^. Furthermore, a recent review suggested that 24 genes previously associated with cardiometabolic phenotypes are also associated with mood disorders (major depressive disorder (MDD) and BD)^26^. An overview of studies previously investigating the role of genes potentially involved in both BD and obesity or T2D^16,17,21,27–36^ is reported in Table 1. The majority of these studies investigated variants already known to be associated with T2D or other metabolic phenotypes.

**Table 1:** Overview of previous studies investigating molecular links between bipolar disorder and obesity or T2D in humans.

Sample	Main results	Study
Post-mortem dlPFC samples from 268 patients (SCZ = 113, MD = 155)^a^ and 191 HC; hippocampal samples from 219 patients (SCZ = 96, MD = 113)^a^ and 169 HC^a^	Compared to HC, patients with mood disorders showed differential expression of *GLP-1R* and *GLP-2R* in the dlPFC and of *GLP-1R* in the hippocampus	Mansur et al.^27^
Post-mortem dlPFC samples from 268 patients (SCZ = 113, MD = 155)^a^ and 191 HC; hippocampal samples from 219 patients (SCZ = 96, MD = 113)^a^ and 169 HC	Significant diagnosis by BMI interaction modulates expression of genes involved in the reeling pathway (*RELN, CAMK2A, CAMK2N2* and *GRIN2A*)	Brietzke et al.^17^
Post-mortem dlPFC samples from 321 patients (SCZ = 142, MDD = 99, BD = 80) and 209 HC; hippocampal samples from 196 patients (SCZ = 102, MDD = 52, BD = 42) and 180 HC	Changes in the expression of insulin receptor-related genes in the postmortem brain tissue of patients with mood and psychotic disorders mediate the expression of dopamine regulation-related genes	Mansur et al.^16^
100 patients with BD type II	Association between the TT genotype of the *GNB3* C825T variant and lower BMI in patients treated with valproate	Chen et al.^28^
284 patients with psychosis (32 with BD)	The addition of genetic to clinical data does not improve prediction of BMI or BMI gain after 1 year	Harrison et al.^29^
662 patients with BD and 616 HC	Interactions between different *TCF7L2* variants and BMI modulate susceptibility to BD	Cuellar-Barboza^30^
139 patients with BD and 137 HC	Association between the Met allele of the *BDNF* Val66Met variant and lower frequency of overweight and obesity	Morales-Marin et al.^31^
384 probands (SCZ = 129, SAD = 85, BD = 160), 413 non-affected relatives and 218 HC	Lack of association between a T2D PRS and proband or relative status; lack of association between a SCZ PRS and prevalence of diabetes	Padmanabhan et al.^32^
90 patients with BD, 76 with SCZ or SAD	Association between the Met66 allele of the *BDNF* Val66Met functional variant and weight gain in patients treated with atypical antipsychotics	Bonaccorso et al.^33^
81 patients with SCZ or BD^a^	Association between *CYP2D6* phenotype and weight gain in patients treated with atypical antipsychotics	Nussbaum et al.^34^
388 BD patients and 1020 HC	Interaction between the *TCF7L2* rs12772424 variant and BMI in modulating susceptibility to BD	Winham et al.^21^
930 patients with SCZ, 869 with BD and 876 HC	No association between BD and 32 SNPs previously associated with T2D	Kajio et al.^35^
96 patients with BD	Association between the T allele of the *GNB3* C825T variant and lower BMI in patients treated with valproate	Chang et al.^36^

*BD* bipolar disorder, *dlPFC* dorsolateral prefrontal cortex, *HC* healthy controls, *MD* mood disorders, *MDD* major depressive disorder, *PRS* polygenic risk score, *SAD* schizoaffective disorder, *SCZ* schizophrenia

^a^The number of mood disorder patients with a diagnosis of BD was not specified

Since the potential molecular mechanisms that could predispose BD patients to an increased susceptibility to obesity or T2D are largely unknown, studies not restricted to specific candidate genes or pathways, able to identify novel molecular targets and pathways commonly associated with BD and metabolic phenotypes, are urgently needed. Genetic variants suggested to be associated to BD or metabolic phenotypes by previous studies generally showed small effect sizes, as in the case of most complex phenotypes. To this regard, gene-based analysis is a powerful method to identify novel genes associated with a complex trait, as this method is able to globally evaluate the cumulative effect of multiple SNPs located in a gene. This approach gives the opportunity to build new knowledge upon genome-wide association studies (GWAS) that have already been produced with huge investments, thus accelerating the discovery of associations between genetic variation and complex traits. To our knowledge, no study has hitherto used this approach to investigate targets commonly associated with BD and metabolic phenotypes. In this sense, public databases of GWAS conducted on large samples of patients affected by BD or metabolic conditions and healthy controls represent an extraordinary opportunity to expand our knowledge on genes that might be implicated in these conditions.

In the present study, we took advantage of large publicly available GWAS datasets to conduct a gene-based analysis as well as cross-trait meta-analyses aiming at (1) identifying genes commonly associated with BD and BMI or BD and T2D, and (2) investigating if genes shared between these conditions are enriched for pathways previously implicated in BD pathophysiology or in the mechanism of action of medications used to treat BD.

## Material and methods

### Sample

The present study was conducted on the summary statistics of three large public GWAS datasets. Summary statistics include all SNPs that were analyzed, together with the calculated effect sizes. Evaluation of BD-associated genes was performed using the summary statistics from the largest GWAS conducted by the Psychiatric Genomics Consortium (PGC) Bipolar Disorder Working Group to date^37^. The dataset included GWAS results from logistic regression analyses on over 13.4 million autosomal SNPs on 20,352 BD cases according to DSM-IV, ICD-9 or ICD-10 criteria and 31,358 controls of European descent. Detailed characteristics of each included cohort have been described in the work from Stahl and coworkers^37^. Further, to identify genes associated with T2D, we used the DIAGRAM 1000G GWAS meta-analysis Stage 1 dataset, including 26,676 T2D cases and 132,532 Caucasian controls from 18 studies^38^. Criteria for T2D diagnosis differed among the studies and included self-reported diagnosis of diabetes by a physician, self-reported use of medication to treat diabetes, fasting glucose ≥ 7.0 mmol/l, or non-fasting glucose ≥ 11.1 mmol/l, hemoglobin A1c (HbA1c) > = 6.5%^38^. This dataset reported association summary statistics for over 12.06 million autosomal SNPs.

Finally, genes associated with BMI were investigated using the GWAS plus Metabochip meta-analysis dataset from the GIANT Consortium^39^. This dataset included summary association statistics of 322,154 subjects of European ancestry. Participants were recruited from 125 studies (82 with GWAS and 43 with results Metabochip results). In this dataset, over 2.47 million autosomal genotyped or imputed variants were tested for association with transformed BMI residuals in linear regressions assuming an additive genetic model^39^.

### Gene-based analysis

For each of the three datasets, a gene-based analysis was performed with MAGMA^40^, using the FUMA platform^41^. MAGMA is a tool for gene analysis that estimates gene-based statistics taking into account the linkage disequilibrium (LD) of the included SNPs. LD was estimated using the European panel of the 1000 Genomes phase 3 data as reference^42^. Locations of protein-coding genes were defined as the regions from transcription start site to transcription stop site (default option in MAGMA). In case a dataset reported information on imputation quality or frequency of the minor allele (MAF), SNPs with imputation quality score < 0.3 or MAF < 0.01 were excluded. Additionally, in the case of the BD dataset, SNPs that were missing in more than 1% of the subjects were excluded. SNPs not represented in all three datasets as well as ambiguous SNPs (G/C or A/T) were also excluded. Three sets of genes significantly associated with BD, BMI and T2D were obtained. Multiple testing correction was performed according to the Benjamini and Hochberg (BH) procedure^43^ using the p.adjust function in R^44^. The hypergeometric test was used to assess over-representation of genes significantly associated with BD and either BMI or T2D after multiple testing correction. Analyses were conducted using R v. 3.6.1 (ref. ^44^).

### Pathway analysis

Enrichment for non-redundant Gene Ontology (GO) terms and Reactome pathways among the genes commonly associated with BD and BMI, BD and T2D or all three phenotypes was assessed using the WebGestalt functional enrichment analysis tool (www.webgestalt.org/). In order to include the largest number of potentially relevant genes in the pathway analysis, we used the list of genes nominally associated with the traits of interests, with a *p* < 0.05, without applying a correction for multiple testing at this step. The enrichment analysis was performed using the hypergeometric test with default settings (minimum number of genes for a category: 5; multiple-testing adjustment: BH method). In order to obtain more information on the possible interactions between proteins coded by the genes found to be commonly associated with BD and BMI or T2D, a protein–protein interaction (PPI) analysis was conducted using STRING^45^. For this analysis, the interaction score was set to high confidence (score = 0.7) and all the active interaction sources supported by the tool were included (text mining, experiments, databases, co-expression, neighborhood, gene fusion and co-occurrence).

### Meta-analysis and functional effects of SNPs associated with BD and BMI or T2D

In order to pinpoint specific SNPs associated with BD and BMI or T2D, we conducted two meta-analyses between BD and BMI, as well as BD and T2D using Metasoft^46^. This software provides effect estimates, heterogeneity estimates as well as a posterior probability that an effect exists in each study (*m*-value statistics > 0.9). The two meta-analyses were conducted with a conservative random-effect model using the same list of SNPs of the gene-based analysis as input. For both meta-analyses, we selected SNPs with a meta-analysis *p* < 5E−08, an *m*-value > 0.9 and a *p* < 0.05 in each original study. The putative functional role of the significant SNPs was evaluated using RegulomeDB^47^. Among SNPs predicted by RegulomeDB to likely affect binding of transcription factors (score < 3), the presence of significant expression quantitative trait loci (eQTL) in adipose tissue as well as in different brain regions was investigated using GTEx^48^. Finally, the presence of SNPs located in potentially druggable genes was investigated using the Drug Gene Interaction database (DGIdb)^49^.

## Results

### Gene-based analysis

A flow-chart of the analyses is reported in Supplementary Fig. 1. A total of 2,013,566 SNPs that passed quality control and were present in all datasets were used as input for the gene-based analysis. These SNPs allowed conducting a gene-based analysis for 17,455 genes. Gene-based analyses conducted with MAGMA identified 2700, 2144 and 1988 genes nominally associated with BD, BMI and T2D, respectively. Among these, a total of 579, 549 and 173 were significant after multiple testing correction with BH, respectively.

When comparing the three lists, 518 genes were found to be commonly associated with BD and BMI, 52 of which were significant after multiple testing correction (Supplementary Table 1). The number of genes associated with both phenotypes was 2.86-fold higher than expected based on results of the hypergeometric test (*p* = 9.4E−12, Supplementary Table 2). Regarding BD and T2D, 390 genes were associated with both phenotypes, 12 of which were significant after multiple testing correction (Supplementary Table 3). The number of overlapping genes was 2.09-fold higher than expected based on results of the hypergeometric test (*p* = 0.01, Supplementary Table 2). Similarly, genes associated with BD were enriched for targets associated with either BMI or T2D (*p* = 2.6E−12, Supplementary Table 2). Finally, 93 genes, three of which were significant after multiple testing correction, were associated with all investigated phenotypes (Supplementary Table 4). Genes associated with BD were not significantly enriched for targets associated with both BMI and T2D (*p* = 0.076, Supplementary Table 2).

### Pathway analyses

**Fig. 1:**
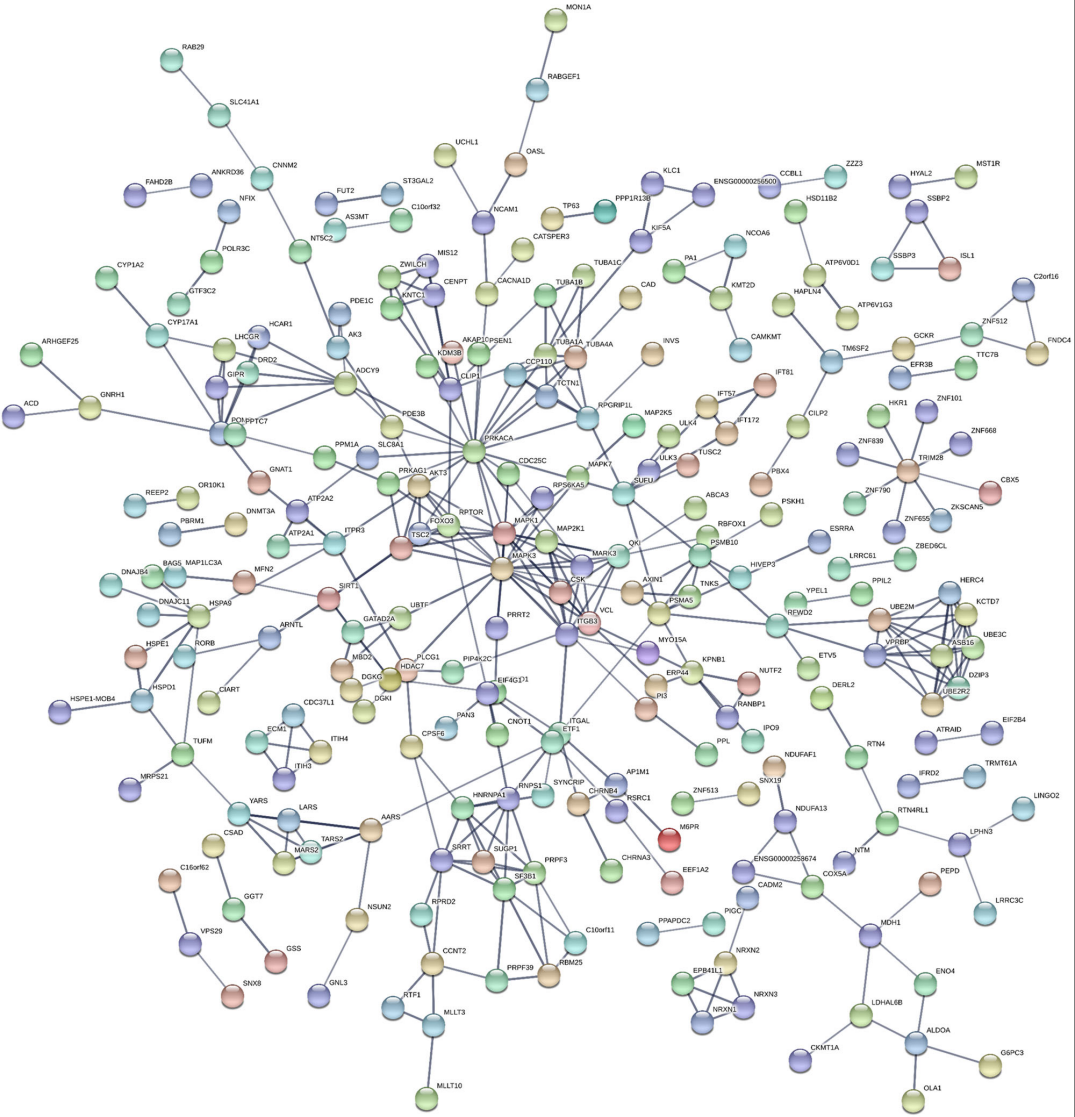
Predicted interactions between proteins encoded by genes commonly associated to bipolar disorder and BMI. Output of the protein–protein interaction analysis conducted using STRING with genes associated with bipolar disorder and BMI as input. Each node represents all the proteins produced by a single protein-coding gene locus (splice isoforms are collapsed), while edges represent protein–protein associations. The interaction score was set to high confidence (score = 0.7) and all the active interaction sources supported by the tool were included (text mining, experiments, databases, co-expression, neighborhood, gene fusion and co-occurrence). The network of proteins encoded by genes commonly associated with bipolar disorder and BMI presents more interactions compared to the number expected for a random set of proteins of similar size extracted from the genome (number of nodes: 504, expected number of edges: 332, observed number of edges: 392, protein–protein interaction enrichment *p* = 0.0007).

The analyses conducted using WebGestalt showed that the genes commonly associated with BD and BMI were enriched for one cellular-component GO term: Neuronal cell body (*p* = 0.0001; false discovery rate (FDR) = 0.025) (Table 2). Additionally, genes commonly associated with BD and BMI were enriched for 12 pathways (Supplementary Table 5). The weighted set cover method implemented by WebGestalt, which reduces redundancy of the gene sets, confirmed a significant association for four of these pathways: Hemostasis, Signaling by Hedgehog, L1CAM interactions and Signaling by BRAF and RAF fusions (Table 2), as well as for the GO term. We further explored the potential interactions between the proteins encoded by the 518 genes commonly associated with BD and BMI using STRING. This analysis showed that the network of proteins encoded by these genes presented a number of interactions greater than expected for a random set of proteins of similar size extracted from the genome (expected number of edges: 332; observed number of edges: 392; PPI enrichment *p* = 0.0007, Fig. 1), supporting the hypothesis that proteins encoded by genes commonly associated with BD and BMI could be biologically connected.

**Table 2:** Weighted set of significantly enriched GO terms and pathways for genes commonly associated with bipolar disorder and BMI.

	Gene set size	Overlap	Enrichment ratio	*p*	FDR	Included genes
GO terms						
Neuronal cell body	486	29	2.08	1.0E−04	0.025	*CAD, CHRNA3, CRTC1, CYP17A1, DDN, DGKI, DRD2, EEF1A2, FXR2, GNAT1, GNRH1, GRIK5, HPCA, ITPR3, KCNN2, KIF5A, KLC1, MAPK1, NRXN1, PARD3, PDE1C, PSEN1, RPTOR, RTN4, RTN4RL1, SLC1A3, SLC4A10, UCHL1, SHTN1*
Pathways						
Hemostasis	620	38	2.25	2.1E−06	0.004	*AK3, AKAP10, ALDOA, ATP2A1, ATP2A2, ATP2B2, CBX5, CDC37L1, CSK, DAGLA, DGKG, DGK1, ECM1, ITGAL, ITGB3, ITIH3, ITIH4, ITPR3, KIF5A, KLC1, MAPK1, MAPK3, MFN2, NEF2, OLA1, PDE3B, PLCG1, PPIL2, PRKACA, SH2B1, SLC7A6, SLC8A1, TUBA1A, TUBA1B, TUBA1C, TUBA4A, VCL, ZFPM2*
Signaling by Hedgehog	147	14	3.50	4.8E−05	0.020	*ADCY9, DERL2, IFT172, IFT57, PRKACA, PSMA5, PSMB10, RPGRIP1L, SUFU, TUBA1A, TUBA1B, TUBA1C, TUBA4A, ULK3*
L1CAM interactions	117	12	3.76	8.1E−05	0.020	*ITGB3, KCNQ2, MAP2K1, MAPK1, MAPK3, NCAM1, RPS6KA5, SHTN1, TUBA1A, TUBA1B, TUBA1C, TUBA4A*
Signaling by BRAF and RAF fusions	60	8	4.89	0.0002	0.030	*CSK, ITGB3, MAP2K1, MAPK1, MAPK3, MARK3, QKI, VCL*

Enriched GO terms and REACTOME pathways were obtained using the weighted set cover method implemented by WebGestalt, which reduces redundancy of the gene sets

*BMI* body mass index, *FDR* false discovery rate

No significantly enriched category of GO terms or pathways was observed for genes commonly associated with BD and T2D. However, the network of proteins encoded by these genes presented more interactions than what would be expected for a random set of proteins of similar size extracted from the genome (expected number of edges: 163; observed number of edges: 215; PPI enrichment *p* = 5.8E−05, Supplementary Fig. 2).

Genes associated with BD, BMI and T2D were not found to be significantly enriched for specific GO terms or pathways. Furthermore, the network of proteins encoded by these genes did not present a significantly higher number of interactions than what would be expected for a random set of proteins of similar size extracted from the genome (expected number of edges: 10; observed number of edges: 10; PPI enrichment *p* = 0.58).

### Meta-analysis and functional effects of SNPs associated with BD and BMI or T2D

The meta-analyses with Metasoft were conducted on the 2,013,566 SNPs for which data were available in all three datasets, which were also used as input for the gene-based analysis. The meta-analysis between BD and BMI identified 64 significant SNPs relevant for both traits (*m*-value > 0.9) (Table 3).

**Table 3:** Results of the meta-analysis showing SNPs significantly associated with bipolar disorder and BMI.

						Meta-analysis			Bipolar disorder			BMI				
SNP	Gene	Chr	Position (hg19)	Effect allele	Other allele	*p*	*B*	SE	OR	SE	*p*	*B*	SE	*p*	Direction	RegDB score
rs1516725	*ETV5*	3	185824004	T	C	1.0E−24	−0.046	0.004	0.94	0.02	0.002	−0.05	0.005	1.9E−22	−−	>=3
rs3888190	*NPIPL1*	16	28889486	A	C	1.1E−24	0.031	0.003	1.03	0.01	0.016	0.03	0.003	3.1E−23	++	1f
rs4788101	*NPIPL1-SH2B1*	16	28867804	T	C	2.1E−24	0.031	0.003	1.03	0.01	0.015	0.03	0.003	4.8E−23	++	>=3
rs7498665	*NPIPL1-SH2B1*	16	28883241	A	G	2.1E−24	−0.031	0.003	0.99	1.14	0.109	−0.03	0.003	9.2E−23	−−	1f
rs8061590	*NPIPL1-SH2B1*	16	28883241	A	G	2.1E−24	−0.031	0.003	0.97	0.01	0.023	−0.03	0.003	4.3E−23	−−	>= 3
rs4234589	*ETV5*	3	185818866	A	G	2.5E−24	0.046	0.004	1.07	0.02	0.002	0.04	0.005	3.8E−22	++	>= 3
rs8055982	*NPIPL1-SH2B1*	16	28881202	A	C	2.8E−24	−0.031	0.003	0.97	0.01	0.015	−0.03	0.003	6.7E−23	−−	1f
rs4788102	*NPIPL1-SH2B1*	16	28873398	A	G	2.8E−24	0.031	0.003	1.03	0.01	0.015	0.03	0.003	7.4E−23	++	1f
rs4788099	*NPIPL1-TUFM*	16	28855727	A	G	2.9E−24	−0.031	0.003	0.97	0.01	0.010	−0.03	0.003	1.1E−22	−−	>= 3
rs7359397	*NPIPL1-SH2B1*	16	28885659	T	C	3.5E−24	0.031	0.003	1.03	0.01	0.020	0.03	0.003	7.4E−23	++	1f
rs12325113	*NPIPL1-ATXN2L*	16	28848668	T	C	5.2E−24	−0.031	0.003	0.97	0.01	0.014	−0.03	0.003	1.3E−22	−−	>= 3
rs12443881	*NPIPL1-ATXN2L*	16	28841777	T	C	6.2E−24	0.030	0.003	1.03	0.01	0.012	0.03	0.003	1.7E−22	++	1 f
rs6809651	*ETV5*	3	185814641	A	G	1.1E−23	−0.046	0.005	0.94	0.02	0.002	−0.05	0.005	1.0E−21	–	>= 3
rs10513801	*ETV5*	3	185822353	T	G	1.3E−23	0.046	0.005	1.07	0.02	0.001	0.04	0.005	1.1E−21	+ +	>= 3
rs7187776	*NPIPL1-ATXN2L*	16	28857645	A	G	3.9E−23	−0.030	0.003	0.97	0.01	0.023	−0.03	0.003	1.1E−21	−−	1 f
rs8049439	*NPIPL1-ATXN2L*	16	28837515	T	C	1.0E−22	−0.030	0.003	0.97	0.01	0.024	−0.03	0.003	1.6E−21	−−	1b
rs7647305	Intergenic	3	185834290	T	C	2.2E−22	−0.036	0.004	0.96	0.02	0.014	−0.04	0.004	1.3E−20	−−	>= 3
rs2008514	*NPIPL1-AK125489*	16	28825605	A	G	3.8E−22	0.030	0.003	1.03	0.01	0.023	0.03	0.003	1.7E−21	++	>= 3
rs8055138	*NPIPL1-ATP2A1*	16	28891465	T	C	9.4E−21	0.031	0.003	1.03	0.01	0.016	0.03	0.003	1.3E−19	++	2b
rs151181	*NPIPL1-CLN3*	16	28490517	T	C	3.0E−19	−0.027	0.003	0.97	0.01	0.021	−0.03	0.003	8.5E−18	−−	>= 3
rs4788084	*NPIPL1*	16	28539848	T	C	6.5E−18	0.026	0.003	1.03	0.01	0.045	0.03	0.003	1.5E−17	++	1a
rs12446550	*NPIPL1*	16	28543381	A	G	6.5E−18	0.026	0.003	1.03	0.01	0.045	0.03	0.003	1.5E−17	++	>= 3
rs7635103	Intergenic	3	185833759	A	C	7.8E−18	0.030	0.004	1.04	0.02	0.006	0.03	0.004	2.8E−16	++	>= 3
rs4788100	*NPIPL1-SH2B1*	16	28864673	T	C	1.9E−15	−0.029	0.004	0.96	0.01	0.010	−0.03	0.004	5.2E−14	−−	>= 3
rs12325278	*NPIPL1*	16	28848818	A	G	3.0E−15	−0.029	0.004	0.97	0.01	0.010	−0.03	0.004	7.8E−14	−−	>= 3
rs10968576	*LINGO2*	9	28414339	A	G	3.4E−15	−0.025	0.003	0.97	0.01	0.020	−0.02	0.003	6.6E−14	−−	>= 3
rs7205323	*NPIPL1-SH2B1*	16	28865892	T	C	3.9E−15	0.029	0.004	1.03	0.01	0.023	0.03	0.004	5.2E−14	++	>= 3
rs7187333	*NPIPL1-SH2B1*	16	28865916	A	G	4.5E−15	0.029	0.004	1.03	0.01	0.028	0.03	0.004	5.2E−14	++	>= 3
rs2183824	*LINGO2*	9	28412078	T	C	4.9E−15	0.025	0.003	1.03	0.01	0.024	0.02	0.003	8.3E−14	++	>= 3
rs8062405	*NPIPL1-ATXN2L*	16	28837906	A	G	5.0E−15	−0.029	0.004	0.97	0.01	0.012	−0.03	0.004	1.2E−13	−−	>= 3
rs2183825	*NPIPL1-ATXN2L*	16	28837906	T	C	9.4E−15	−0.025	0.003	0.97	0.01	0.022	−0.02	0.003	6.9E−14	−−	>= 3
rs16912921	*LINGO2*	9	28413461	A	C	1.0E−14	0.025	0.003	1.03	0.01	0.026	0.02	0.003	9.6E−14	++	>= 3
rs12928404	*NPIPL1-ATXN2L*	16	28847246	T	C	2.9E−14	−0.028	0.004	0.97	0.01	0.038	−0.03	0.004	2.6E−13	−−	1b
rs4234588	*ETV5*	3	185786406	A	G	5.4E−14	0.043	0.006	1.06	0.02	0.002	0.04	0.005	8.5E−18	++	>= 3
rs10968577	*LINGO2*	9	28415512	T	C	4.1E−13	0.026	0.004	1.03	0.01	0.020	0.03	0.004	5.3E−12	++	>= 3
rs1620977	*NEGR1*	1	72729142	A	G	8.7E−13	0.024	0.003	1.03	0.02	0.050	0.02	0.004	5.3E−12	++	>= 3
rs2968487	Intergenic	1	96887370	T	C	3.3E−12	0.025	0.004	1.03	0.01	0.025	0.02	0.004	2.8E−11	++	>= 3
rs587242	Intergenic	1	96886267	A	G	1.3E−11	0.025	0.004	1.04	0.02	0.015	0.02	0.004	2.1E−10	++	>= 3
rs2127162	*MAP2K5*	15	68090824	A	C	4.7E−11	−0.023	0.004	0.97	0.01	0.015	−0.02	0.004	7.2E−10	−−	>= 3
rs1830868	Intergenic	1	96895906	A	G	5.9E−11	0.026	0.004	1.04	0.01	0.012	0.03	0.004	1.1E−09	++	>= 3
rs7543671	Intergenic	1	96892262	T	C	6.9E−11	−0.026	0.004	0.96	0.01	0.016	−0.03	0.004	1.1E−09	−−	>= 3
rs7543590	Intergenic	1	96892216	T	C	8.2E−11	−0.026	0.004	0.96	0.01	0.016	−0.02	0.004	1.3E−09	−−	>= 3
rs661573	Intergenic	1	96891371	A	G	1.1E−10	0.026	0.004	1.03	0.01	0.024	0.02	0.004	1.3E−09	++	>= 3
rs7551466	Intergenic	1	96892427	T	G	1.1E−10	0.025	0.004	1.04	0.01	0.016	0.02	0.004	1.7E−09	++	>= 3
rs1853738	Intergenic	1	96895992	A	G	1.2E−10	−0.025	0.004	0.97	0.01	0.017	−0.02	0.004	1.7E−09	−−	>= 3
rs12758621	Intergenic	1	96898421	T	C	1.2E−10	0.025	0.004	1.04	0.01	0.017	0.02	0.004	1.7E−09	++	>= 3
rs550739	Intergenic	1	96887109	A	G	1.2E−10	0.025	0.004	1.03	0.01	0.023	0.02	0.004	1.5E−09	++	>= 3
rs12030271	Intergenic	1	96892099	A	G	1.3E−10	−0.025	0.004	0.96	0.01	0.016	−0.02	0.004	2.0E−09	−−	>= 3
rs10489740	Intergenic	1	96919182	A	G	1.3E−10	0.025	0.004	1.04	0.01	0.012	0.02	0.004	2.3E−09	++	>= 3
rs722155	Intergenic	1	96906374	A	G	1.4E−10	−0.025	0.004	0.97	0.01	0.017	−0.02	0.004	2.0E−09	−−	>= 3
rs505066	Intergenic	1	96882671	A	C	3.8E−10	0.025	0.004	1.04	0.01	0.021	0.02	0.004	4.7E−09	++	>= 3
rs12739556	Intergenic	1	96977901	T	C	4.5E−10	0.025	0.004	1.04	0.01	0.018	0.02	0.004	6.3E−09	++	>= 3
rs12566597	Intergenic	1	96978626	A	G	6.9E−10	0.025	0.004	1.04	0.01	0.017	0.02	0.004	9.6E−09	++	>= 3
rs1412234	*LINGO2*	9	28410683	T	C	8.5E−10	−0.024	0.004	0.97	0.01	0.023	−0.02	0.004	9.9E−09	−−	>= 3
rs4341405	Intergenic	1	96902589	A	G	9.0E−10	−0.025	0.004	0.97	0.01	0.017	−0.02	0.004	1.3E−08	−−	>= 3
rs4141973	Intergenic	1	50946521	T	C	1.6E−09	0.029	0.005	1.04	0.02	0.042	0.03	0.005	1.5E−08	++	>= 3
rs7541994	Intergenic	1	96984344	A	G	2.1E−09	0.024	0.004	1.03	0.02	0.025	0.02	0.004	2.2E−08	++	>= 3
rs4000303	Intergenic	1	96913765	A	G	4.8E−09	−0.024	0.004	0.97	0.01	0.048	−0.02	0.004	3.6E−08	−−	>= 3
rs1477199	*RPGRIP1L*	16	53712135	A	G	5.7E−09	−0.025	0.004	0.96	0.02	0.035	−0.02	0.004	4.6E−08	−−	>= 3
rs17203016	Intergenic	2	208255518	A	G	1.0E−08	−0.022	0.004	0.96	0.02	0.033	−0.02	0.004	8.1E−08	−−	>= 3
rs6671342	Intergenic	1	97000957	T	G	1.6E−08	−0.023	0.004	0.97	0.02	0.031	−0.02	0.004	1.4E−07	−−	>= 3
rs4729098	*DTX2P1-UPK3BP1-PMS2P11*	7	76637070	A	G	2.9E−08	−0.037	0.007	0.96	0.02	0.043	−0.04	0.007	2.3E−07	−−	>= 3
rs12751763	Intergenic	1	96997655	T	C	3.6E−08	0.023	0.004	1.03	0.02	0.040	0.02	0.004	2.8E−07	++	>= 3
rs2814944	Intergenic	6	34552797	A	G	4.4E−08	0.023	0.004	1.04	0.02	0.050	0.02	0.004	2.2E−07	++	>= 3

SNPs showing a RegulomeDB score < 3 are more likely to affect binding of transcription factors

*BMI* body mass index, *Chr* chromosome, *OR* odds ratio, *RegDB* RegulomeDB, *SE* standard error, *SNP* single nucleotide polymorphism

The majority of SNPs shared between BD and BMI were located in intergenic regions (*n* = 26) or in a locus in chromosome 16 spanning the *NPIPL1, SH2B1, TUFM, ATP2A1, AK125489, CLN3* and *ATXN2L* genes (*n* = 24 SNPs; best SNP: rs3888190, *p* = 1.1E−24). Five SNPs were located in the *ETV5* gene (best SNP: rs1516725, *p* = 1.0E−24), five in the *LINGO2* gene (best SNP: rs2183824, *p* = 4.9E−15), while the remaining four SNPs were located in the *NEGR1* (rs1620977, *p* = 8.7E−13), *MAP2K5* (rs2127162, *p* = 4.7E−11), *RPGRIP1L* (rs1477199, *p* = 5.7E−09) and *DTX2P1* genes (rs4729098, *p* = 2.9E−08). Interestingly, three of these genes (*RPGRIP1L, SH2B1* and *ATP2A1*) were included in significantly enriched pathways identified by WebGestalt. Specifically, *RPGRIP1L* was included in the Signaling by Hedgehog pathway, while *SH2B1* and *ATP2A1* in the Hemostasis pathway (Table 2).

A total of 11 SNPs located in three genes were predicted to affect the binding of transcription factors by RegulomeDB (score < 3, Table 3). All SNPs for which RegulomeDB predicted a functional effect were also found to be significant eQTLs in adipose tissue and in different brain regions in GTEx (Supplementary Table 6). Finally, 13 SNPs were located in genes that are part of the druggable genome (*LINGO2, NEGR1, MAP2K5, DTX2P1* and *ATP2A1*) according to DGIdb.

The meta-analysis between BD and T2D identified six SNPs relevant for both traits (Table 4), three of which were located in the *ALAS1* gene (best SNP: rs352165, *p* = 3.4E−08), two in intergenic regions and one in the *KCNG1* gene (rs6091248, *p* = 4.3E−08). The three SNPs rs61428, rs352162 (intergenic regions) and rs164640 (*ALAS1*) were predicted to affect the binding of transcription factors by RegulomeDB (Table 4) and were also found to be significant eQTLs in cerebellum (Supplementary Table 6). *KCNG1* was found to be part of the druggable genome by DGIdb.

**Table 4:** Results of the meta-analysis showing SNPs significantly associated with bipolar disorder and type 2 diabetes.

						Meta-analysis			Bipolar disorder			T2D				
SNP	Gene	Chr	Position (hg19)	Effect allele	Other allele	Beta	SE	*p*	OR	SE	*p*	*B*	SE	*p*	Direction	RegDB score
rs614288	Intergenic	3	52220203	T	C	0.05	0.01	2.2E−08	1.05	0.01	8.8E−05	0.05	0.01	8.9E−05	++	1f
rs352165	*ALAS1*	3	52242902	A	G	0.05	0.01	2.6E−08	1.06	0.01	2.2E−05	0.04	0.01	3.2E−04	++	>= 3
rs164640	*ALAS1*	3	52247314	T	C	−0.05	0.01	3.3E−08	0.94	0.01	1.9E−05	−0.04	0.01	3.5E−04	−−	1f
rs352162	Intergenic	3	52252969	T	C	0.05	0.01	3.3E−08	1.06	0.01	4.4E−06	0.04	0.01	3.4E−04	++	1d
rs352163	*ALAS1*	3	52247110	A	G	−0.05	0.01	3.5E−08	0.94	0.01	1.9E−05	−0.04	0.01	4.0E−04	−−	>= 3
θrs6091248	*KCNG1*	20	49630618	A	G	0.05	0.01	4.3E−08	1.06	0.01	1.7E−05	0.05	0.01	3.7E−04	++	>= 3

SNPs showing a RegulomeDB score <3 are more likely to affect binding of transcription factors

*Chr* chromosome, *OR* odds ratio, *RegDB* RegulomeDB, *SE* standard error, *SNP* single nucleotide polymorphism

Finally, we found no SNP in common between the two meta-analyses (i.e. SNPs significant in both comparisons BD and BMI as well as BD and T2D)

## Discussion

Our study supports the existence of shared genetic factors between BD and BMI. To the best of our knowledge, this is the first study to investigate genes commonly associated with BD, BMI and T2D using a gene-based approach and without a specific focus on single candidate genes and pathways previously suggested to be involved in BD. We showed that BD and BMI share a higher number of susceptibility genes compared to BD and T2D. Some of the genes we found to be associated with BD and either BMI or T2D (*CACNA1D, ITIH4, NCAN, CRY2* and *POMC*) were previously reported by a recent systematic review to be associated with cardiometabolic phenotypes and mood disorders (MDD and BD)^26^. Besides validating these genes, we reported many novel targets and found that genes commonly associated with BD and BMI are enriched for the cellular-component Neuronal cell body GO term.

The pathway analysis from WebGestalt further supports the hypothesis that genes commonly associated with BD and BMI show a significant functional enrichment. Two of the pathways (Signaling by Hedgehog and Hedgehog “off” state) were related to Hedgehog signaling and the former was also included among the four pathways of the weighted set (i.e. the set including the most representative pathways that can cover all the genes from the enriched sets). The Hedgehog signaling pathway plays a crucial role in neural and limb development, cell growth, differentiation and survival^50^. The mechanisms underlying Hedgehog signaling are complex and not completely understood. This pathway takes the name from the Hedgehog (Hh) ligand, which was first identified in *D. melanogaster*^51^. This ligand has three counterparts in mammalians: sonic hedhgehog (SHH), Indian hedgehog (IHH) and desert hedgehog (DHH)^52^. Through a finely regulated switching between “off” and “on” states, the Hedgehog pathway modulates a signaling cascade that ultimately targets the Gli transcription factors. In the absence of the ligand (Hedgehog off state), the cytosolic Gli proteins are cleaved to a truncated form, translocate to the nucleus and repress gene expression. Conversely, in the presence of the Hedgehog ligand (Hedgehog on state), the Gli transcription factors are stabilized in their full-length form, which is able to stimulate gene expression^50^.

Recent evidence suggests that the Hedgehog transduction pathway shows a bi-directional connection with metabolism^53^. In fact, on one hand Hedgehog proteins are modified by fatty acids and cholesterol (these modifications being essential for their maturation and activity)^53^. On the other hand, this signaling system might play a role in adipocyte differentiation and regulation of energy homeostasis^53^. Although the role of the Hedgehog pathway in the pathophysiology of BD has not been systematically investigated, disruption of sonic hedgehog signaling associated with Ellis-van Creveld syndrome (a form of chondrodysplastic dwarfism) has been suggested to exert a protective role against BD^54^. Interestingly, in vitro treatment with lithium was found to modulate Hedgehog signaling in pancreatic adenocarcinoma cells via the inhibition of the GSK-3 serine/threonine kinase^55^. In this study, lithium exerted a biphasic effect, i.e. an initial increase of GLI1 cellular levels due to the inhibition of the ubiquitin-proteasome-mediated GLI degradation, followed by a downregulation of expression and activity of GLI1 after lithium treatment for 18 h^55^. These results suggest that lithium might interfere with Hedgehog signaling via modulation of the ubiquitin-proteasome degradation.

The meta-analysis approach also supported the hypothesis that genes involved in Hedgehog signaling might play an important role. In fact, among SNPs significantly associated with BD and BMI, rs1477199 was located in the *RPGRIP1L* gene. This gene encodes a protein localized at the transition zone of the primary cilium^56^ and is required for hypothalamic arcuate neuron development^57^. *RPGRIP1L* represents a particularly interesting target, as its expression and activity are regulated by intronic variants located in the *FTO* gene (which is strongly associated with obesity and T2D) through long-range regulation^58^. *RPGRIP1L* was recently associated with BD in a sample including 276 patients and 170 controls of Mexican origin^59^. Taken together, our findings suggest that complementary analytical approaches may provide converging evidence and should be used together to be able to identify genes in which multiple SNPs with small effect sizes might play an additive effect, as well as genes in which single SNPs might play a more relevant role. The two different approaches we used support the potential role of Hedgehog signaling in both BD and BMI.

Other relevant pathways shared between BD and BMI were revealed, such as “L1CAM interactions”, which has been implicated in neuritogenesis and neuroprotection^60^. Indeed, the neural cell adhesion molecule L1 plays a crucial role in nervous system development, being involved in neurite outgrowth, adhesion, axon guidance, myelination and synaptic plasticity^61^. Preclinical studies support the hypothesis that dysregulation of neuronal synaptic plasticity might mediate a potential role of L1 and other adhesion molecules in the pathogenesis of mood disorders^62^. Interestingly, state-dependent alterations of L1 peripheral messenger RNA (mRNA) levels in patients with BD have been suggested^63^. Specifically, L1 levels were increased in a sample of 13 BD patients in a current depressive state, but not in patients in a remissive state (*n* = 29)^63^.

Results from our meta-analysis confirmed the important role played by other genes previously suggested to be associated with BD and BMI. Specifically, we found five SNPs to be located in the *ETV5* gene. Inhibition of the *ETV5* homolog in *D. melanogaster (Ets96B*) induces BD- and obesity-related phenotypes^20^.

Our hypothesis that genetic variants might contribute to explain the increased comorbidity between BD and obesity is in contrast with findings from a recent study showing that the inclusion of genetic data in a model comprising clinical characteristics did not improve prediction of BMI or BMI gain after 1 year in a sample of 284 patients with psychosis^29^. However, in this study only 32 patients had a diagnosis of BD, suggesting the need to conduct further studies specifically including BD patients.

While our results support the existence of shared functional pathways between BD and BMI, we did not observe a functional enrichment for genes commonly associated with BD and T2D. However, proteins encoded by these genes showed more interactions compared to what would be expected for a random set or proteins of similar size extracted from the genome. The limited number of genetic targets we observed to be shared between BD and T2D is in accordance with previous studies showing a lack of association between T2D polygenic risk scores (PRS) and diagnosis of psychiatric disorders^32,35^. Although the aggregated effect of multiple T2D-associated variants might not play a relevant role in BD pathophysiology, the contribution of specific genes might still be important. In our meta-analysis, the largest number of SNPs commonly associated with BD and T2D was located in the *ALAS1* gene, which might represent a promising target to further investigate. This gene catalyzes the rate-limiting step in heme (iron-protoporphyrin) biosynthesis, a process which has been shown to be bidirectionally connected with the regulation of the circadian clock^64,65^. Based on a large body of evidence supporting the existence of circadian disturbances in patients with BD^66^ as well as the important role of circadian genes in both BD and response to lithium^67–70^, it might be important for future studies to evaluate the role of genes interacting with circadian systems as in the case of *ALAS1*.

Our results have to be interpreted in light of some limitations. Firstly, we chose to select genes nominally associated with both BD and BMI or T2D in GWAS datasets without applying a correction for multiple testing at this step, in order to include the largest number of potentially relevant genes in the pathway analysis. Although this might have increased the risk to include less-relevant genes in the pathway analysis, this aspect was partly addressed by applying a FDR correction on pathways as well as searching for converging evidence from the meta-analysis approach to support the potential relevance of the identified pathways. Secondly, publicly available GWAS datasets do not generally include information on concomitant disturbances. Therefore, it was not possible to assess the prevalence of BD as well as other psychiatric comorbidities in the BMI and T2D datasets. As our analyses could not be adjusted for potential comorbidities, it is not possible to exclude that comorbidity with psychiatric disorders could play a role in explaining our findings. Thirdly, although GWAS summary statistics can be analyzed with a wide range of methods, they do not provide individual-level genotyping data, thus not allowing to conduct other analyses that can provide important insight such as PRS analysis. Finally, although we conducted our analyses on the largest and most recent BD dataset released by the PGC to date, the smaller number of participants included in this dataset compared to the BMI and T2D GWAS datasets might have limited our ability to identify shared genetics determinants between BD and the other traits. The main strengths of our study are the large sample size of the included datasets and the choice not to restrict our investigation to specific candidate genes or pathways to overcome limitations of previous studies focused on candidate targets.

It is important to note that our analyses were conducted on subjects of European ancestry. Further studies will be needed to assess the potential generalizability of these results to other populations. While our study was focused on BD, other psychiatric disorders such as MDD and schizophrenia show increased frequency of obesity^71^. A recent study investigating the role of the candidate gene *CADM2*, which encodes a synaptic cell adhesion molecule, reported that this gene might be associated with a wide range of psychological (neuroticism, mood instability and risk-taking) and metabolic traits, and that regulation of this gene in adipose tissue might mediate common biological mechanisms across phenotypes^72^. In accordance with these results, our gene-based analysis identified this gene as commonly associated with BD and BMI and this association survived multiple testing correction. Further studies will be needed to understand which of the targets we identified might be specific for BD and which genes or pathways might play a role in different psychiatric disorders.

To conclude, our results suggest the existence of shared genetic determinants between BD and BMI and support the relevance of genes implicated in Hedgehog signaling. Studies using other instruments such as PRS to further evaluate shared etiology between these traits, as well as to assess the aggregated effect of multiple variants with small effect sizes, are warranted. Future studies on independent samples for which information on comorbidities and potential confounding factors are available are needed to confirm our results and explore the potential role of these genes in the mechanism of action of mood stabilizers in patients with BD. Finally, based on the fact that some of the targets and pathways we reported to be associated with both BD and BMI have been implicated in the mechanism of action of lithium, it would be important to explore if at least part of the genetic determinants common to these traits might play a role in response to this mood stabilizer.

## Supplementary information

Supplementary Figure and Table Legends

Supplementary Table 1

Supplementary Table 2

Supplementary Table 3

Supplementary Table 4

Supplementary Table 5

Supplementary Table 6

Supplementary Figure 1

Supplementary Figure 2

## Data Availability

Datasets used in this article are publicly available and can be downloaded from the Psychiatric Genomics Consortium, the DIAGRAM Consortium and the GIANT consortium websites.

## Code availability

Code is available upon request.
