# Supplementary material for: Evidence that genes involved in hedgehog signaling are associated with both bipolar disorder and high BMI
Source: Transl Psychiatry. 2019 Nov 21;9:315. doi: 10.1038/s41398-019-0652-x (PMC6872724; doi:10.1038/s41398-019-0652-x)
Supplement: Supplementary file 1 — Supplementary Figure and Table Legends [file 41398_2019_652_MOESM1_ESM.docx]

**Supplementary Figures Title and Legends**

**Supplementary Figure 1. Flow-chart of the analyses showing genes associated with Bipolar Disorder, BMI and Type 2 Diabetes**

Abbreviations: BD, bipolar disorder; BMI, body mass index; n, number; SNP, single nucleotide polymorphism; T2D, type 2 diabetes.

The lists of genes and SNPs commonly associated with BD and BMI or BD and T2D are reported in Supplementary Tables 1 and 3 (genes), Tables 3 and 4 (SNPs), respectively.

**Supplementary Figure 2. Predicted interactions between proteins encoded by genes commonly associated to bipolar disorder and type 2 diabetes**

Output of the protein-protein interaction analysis conducted using STRING with genes associated with bipolar disorder and type 2 diabetes as input. Each node represents all the proteins produced by a single protein-coding gene locus (splice isoforms are collapsed), while edges represent protein-protein associations. The interaction score was set to high confidence (score = 0.7) and all the active interaction sources supported by the tool were included (text mining, experiments, databases, co-expression, neighborhood, gene fusion and co-occurrence). The network of proteins encoded by genes commonly associated with bipolar disorder and type 2 diabetes presents a number of interactions greater than expected for a random set of proteins of similar size extracted from the genome (number of nodes: 380, expected number of edges: 163, observed number of edges: 215, protein-protein interaction enrichment p-value = 5.8E-05)
