# Supplementary material for: Evidence that genes involved in hedgehog signaling are associated with both bipolar disorder and high BMI
Source: Transl Psychiatry. 2019 Nov 21;9:315. doi: 10.1038/s41398-019-0652-x (PMC6872724; doi:10.1038/s41398-019-0652-x)
Supplement: Supplementary file 2 — Supplementary Table 1 [file 41398_2019_652_MOESM2_ESM.docx]

**Supplementary Table 1. Genes associated with both bipolar disorder and BMI in the gene-based analysis conducted with MAGMA**

|  |  |  |  | **Bipolar disorder** | | | **BMI** | | |
| --- | --- | --- | --- | --- | --- | --- | --- | --- | --- |
| **Gene** | **Chr** | **# SNPS** | **NPARAM** | **Z** | **p** | **adj p (BH)** | **Z** | **p** | **adj p (BH)** |
| ***ITIH3*** | **3** | **7** | **2** | **5.51** | **1.8E-08** | **4.97E-05** | **3.37** | **0.0004** | **0.0190** |
| ***TSSK6*** | **19** | **1** | **1** | **5.29** | **6.1E-08** | **0.0001** | **3.30** | **0.0005** | **0.0230** |
| ***HAPLN4*** | **19** | **2** | **1** | **5.15** | **1.3E-07** | **0.0001** | **2.98** | **0.0015** | **0.0481** |
| ***MAPK1*** | **22** | **66** | **7** | **4.93** | **4.1E-07** | **0.0003** | **3.00** | **0.0014** | **0.0465** |
| ***CILP2*** | **19** | **2** | **1** | **4.88** | **5.3E-07** | **0.0004** | **3.43** | **0.0003** | **0.0168** |
| ***NDUFA13*** | **19** | **7** | **2** | **4.84** | **6.4E-07** | **0.0004** | **3.78** | **0.0001** | **0.0067** |
| ***CTC-260F20.3*** | **19** | **9** | **2** | **4.76** | **9.7E-07** | **0.0005** | **3.68** | **0.0001** | **0.0088** |
| ***YJEFN3*** | **19** | **9** | **2** | **4.76** | **9.7E-07** | **0.0005** | **3.68** | **0.0001** | **0.0088** |
| ***ITIH1*** | **3** | **16** | **2** | **4.65** | **1.7E-06** | **0.0008** | **3.44** | **0.0003** | **0.0166** |
| ***KMT2D*** | **12** | **11** | **2** | **4.63** | **1.9E-06** | **0.0008** | **4.11** | **2.0E-05** | **0.0023** |
| ***TFAP2B*** | **6** | **22** | **6** | **4.60** | **2.1E-06** | **0.0008** | **7.28** | **1.7E-13** | **2.4E-10** |
| ***TM6SF2*** | **19** | **3** | **1** | **4.54** | **2.8E-06** | **0.0008** | **3.41** | **0.0003** | **0.0173** |
| ***PRKAG1*** | **12** | **1** | **1** | **4.36** | **6.5E-06** | **0.0014** | **3.53** | **0.0002** | **0.0127** |
| ***NCOA6*** | **20** | **42** | **4** | **4.27** | **9.6E-06** | **0.0018** | **3.27** | **0.0005** | **0.0245** |
| ***C10orf32*** | **10** | **8** | **3** | **4.26** | **1.0E-05** | **0.0019** | **5.13** | **1.5E-07** | **3.7E-05** |
| ***GGT7*** | **20** | **8** | **2** | **4.22** | **1.2E-05** | **0.0021** | **3.46** | **0.0003** | **0.0154** |
| ***MAU2*** | **19** | **17** | **3** | **4.16** | **1.6E-05** | **0.0026** | **3.58** | **0.0002** | **0.0117** |
| ***PDE1C*** | **7** | **618** | **76** | **4.13** | **1.8E-05** | **0.0028** | **3.35** | **0.0004** | **0.0200** |
| ***STAB1*** | **3** | **14** | **4** | **4.08** | **2.3E-05** | **0.0034** | **3.20** | **0.0007** | **0.0292** |
| ***CENPT*** | **16** | **5** | **2** | **4.01** | **3.1E-05** | **0.0041** | **3.28** | **0.0005** | **0.0241** |
| ***C10orf32-ASMT*** | **10** | **32** | **6** | **3.97** | **3.5E-05** | **0.0046** | **5.24** | **8.1E-08** | **2.2E-05** |
| ***CYP17A1*** | **10** | **9** | **2** | **3.97** | **3.6E-05** | **0.0046** | **4.92** | **4.3E-07** | **0.0001** |
| ***SUGP1*** | **19** | **22** | **5** | **3.89** | **4.9E-05** | **0.0055** | **3.55** | **0.0002** | **0.0124** |
| ***GATAD2A*** | **19** | **48** | **4** | **3.86** | **0.0001** | **0.0059** | **3.79** | **0.0001** | **0.0064** |
| ***C2orf16*** | **2** | **4** | **2** | **3.77** | **0.0001** | **0.0075** | **3.01** | **0.0013** | **0.0452** |
| ***GCKR*** | **2** | **13** | **3** | **3.75** | **0.0001** | **0.0078** | **3.08** | **0.0010** | **0.0388** |
| ***UBAP2*** | **9** | **72** | **8** | **3.71** | **0.0001** | **0.0087** | **3.39** | **0.0004** | **0.0184** |
| ***RBFOX1*** | **16** | **2551** | **302** | **3.64** | **0.0001** | **0.0102** | **2.97** | **0.0015** | **0.0485** |
| ***LMBR1L*** | **12** | **5** | **1** | **3.62** | **0.0001** | **0.0107** | **3.41** | **0.0003** | **0.0173** |
| ***BRE*** | **2** | **252** | **23** | **3.57** | **0.0002** | **0.0120** | **3.24** | **0.0006** | **0.0261** |
| ***UBE2R2*** | **9** | **54** | **9** | **3.55** | **0.0002** | **0.0129** | **3.90** | **4.9E-05** | **0.0046** |
| ***NFIX*** | **19** | **20** | **5** | **3.54** | **0.0002** | **0.0130** | **3.12** | **0.0009** | **0.0360** |
| ***CADM2*** | **3** | **700** | **41** | **3.51** | **0.0002** | **0.0140** | **6.89** | **2.9E-12** | **2.7E-09** |
| ***NCAM1*** | **11** | **303** | **29** | **3.50** | **0.0002** | **0.0144** | **5.84** | **2.7E-09** | **1.0E-06** |
| ***TAOK2*** | **16** | **11** | **1** | **3.37** | **0.0004** | **0.0200** | **4.28** | **9.4E-06** | **0.0013** |
| ***GPN3*** | **12** | **4** | **2** | **3.29** | **0.0005** | **0.0237** | **3.16** | **0.0008** | **0.0325** |
| ***ETV5*** | **3** | **23** | **3** | **3.27** | **0.0005** | **0.0250** | **7.28** | **1.6E-13** | **2.4E-10** |
| ***TSNARE1*** | **8** | **108** | **14** | **3.24** | **0.0006** | **0.0272** | **3.24** | **0.0006** | **0.0263** |
| ***TMEM219*** | **16** | **3** | **1** | **3.21** | **0.0007** | **0.0286** | **4.06** | **2.4E-05** | **0.0027** |
| ***TP53INP2*** | **20** | **4** | **1** | **3.20** | **0.0007** | **0.0289** | **2.97** | **0.0015** | **0.0485** |
| ***KNTC1*** | **12** | **32** | **6** | **3.20** | **0.0007** | **0.0292** | **3.11** | **0.0009** | **0.0365** |
| ***RND1*** | **12** | **3** | **2** | **3.18** | **0.0007** | **0.0307** | **2.97** | **0.0015** | **0.0484** |
| ***HS6ST3*** | **13** | **518** | **43** | **3.17** | **0.0008** | **0.0309** | **4.96** | **3.5E-07** | **0.0001** |
| ***SPHKAP*** | **2** | **154** | **18** | **3.16** | **0.0008** | **0.0319** | **3.39** | **0.0003** | **0.0183** |
| ***ARNTL*** | **11** | **103** | **16** | **3.14** | **0.0009** | **0.0332** | **3.40** | **0.0003** | **0.0181** |
| ***MSRA*** | **8** | **545** | **43** | **3.13** | **0.0009** | **0.0332** | **3.37** | **0.0004** | **0.0190** |
| ***RPGRIP1L*** | **16** | **67** | **8** | **3.11** | **0.0009** | **0.0347** | **3.50** | **0.0002** | **0.0139** |
| ***PRMT7*** | **16** | **36** | **4** | **3.03** | **0.0012** | **0.0415** | **3.80** | **0.0001** | **0.0063** |
| ***VPS29*** | **12** | **2** | **1** | **3.03** | **0.0012** | **0.0420** | **4.02** | **2.9E-05** | **0.0031** |
| ***GIPC2*** | **1** | **72** | **10** | **3.03** | **0.0012** | **0.0423** | **4.86** | **5.8E-07** | **0.0001** |
| ***KDM3B*** | **5** | **35** | **6** | **3.02** | **0.0012** | **0.0424** | **3.03** | **0.0012** | **0.0433** |
| ***DGKG*** | **3** | **214** | **48** | **3.00** | **0.0013** | **0.0446** | **4.32** | **7.6E-06** | **0.0010** |
| *SSBP2* | 5 | 180 | 20 | 5.63 | 9.1E-09 | 4.97E-05 | 2.76 | 0.0029 | 0.0761 |
| *ITIH4* | 3 | 11 | 3 | 5.41 | 3.2E-08 | 0.0001 | 2.46 | 0.0070 | 0.1346 |
| *RP5-966M1.6* | 3 | 11 | 3 | 5.41 | 3.2E-08 | 0.0001 | 2.46 | 0.0070 | 0.1346 |
| *SPCS1* | 3 | 1 | 1 | 4.96 | 3.5E-07 | 0.0003 | 2.54 | 0.0056 | 0.1162 |
| *NEK4* | 3 | 13 | 3 | 4.96 | 3.5E-07 | 0.0003 | 2.19 | 0.0142 | 0.2043 |
| *TMEM110-MUSTN1* | 3 | 43 | 5 | 4.87 | 5.4E-07 | 0.0004 | 2.37 | 0.0090 | 0.1557 |
| *ANKS1B* | 12 | 903 | 97 | 4.86 | 5.8E-07 | 0.0004 | 2.64 | 0.0042 | 0.0965 |
| *GNL3* | 3 | 7 | 2 | 4.84 | 6.6E-07 | 0.0004 | 2.45 | 0.0071 | 0.1359 |
| *TMEM110* | 3 | 42 | 5 | 4.86 | 6.0E-07 | 0.0004 | 2.34 | 0.0096 | 0.1628 |
| *PBRM1* | 3 | 52 | 3 | 4.84 | 6.4E-07 | 0.0004 | 2.22 | 0.0131 | 0.1982 |
| *STARD9* | 15 | 48 | 4 | 4.78 | 8.8E-07 | 0.0005 | 1.65 | 0.0498 | 0.4065 |
| *SMIM4* | 3 | 18 | 2 | 4.74 | 1.1E-06 | 0.0005 | 1.96 | 0.0253 | 0.2898 |
| *MAP1LC3A* | 20 | 2 | 1 | 4.64 | 1.7E-06 | 0.0008 | 2.80 | 0.0026 | 0.0697 |
| *ATP13A1* | 19 | 10 | 2 | 4.64 | 1.7E-06 | 0.0008 | 2.53 | 0.0057 | 0.1168 |
| *DDN* | 12 | 2 | 1 | 4.58 | 2.3E-06 | 0.0008 | 2.91 | 0.0018 | 0.0552 |
| *RBPJL* | 20 | 6 | 2 | 4.55 | 2.7E-06 | 0.0008 | 2.86 | 0.0021 | 0.0629 |
| *PPM1F* | 22 | 28 | 6 | 4.55 | 2.7E-06 | 0.0008 | 2.70 | 0.0035 | 0.0859 |
| *SLC4A1* | 17 | 9 | 4 | 4.59 | 2.3E-06 | 0.0008 | 2.13 | 0.0166 | 0.2268 |
| *C17orf53* | 17 | 8 | 2 | 4.51 | 3.3E-06 | 0.0009 | 1.68 | 0.0467 | 0.3898 |
| *GLT8D1* | 3 | 4 | 1 | 4.48 | 3.7E-06 | 0.0010 | 1.99 | 0.0232 | 0.2779 |
| *SFMBT1* | 3 | 60 | 7 | 4.49 | 3.5E-06 | 0.0010 | 1.72 | 0.0429 | 0.3761 |
| *AC074091.13* | 2 | 6 | 2 | 4.45 | 4.2E-06 | 0.0010 | 1.83 | 0.0334 | 0.3371 |
| *SP4* | 7 | 68 | 11 | 4.34 | 7.2E-06 | 0.0015 | 1.74 | 0.0408 | 0.3697 |
| *CDKN2C* | 1 | 3 | 1 | 4.30 | 8.7E-06 | 0.0017 | 2.86 | 0.0021 | 0.0619 |
| *EEF1A2* | 20 | 5 | 3 | 4.20 | 1.3E-05 | 0.0022 | 1.84 | 0.0328 | 0.3336 |
| *KCTD9* | 8 | 29 | 2 | 4.16 | 1.6E-05 | 0.0025 | 2.54 | 0.0055 | 0.1159 |
| *FAF1* | 1 | 138 | 16 | 4.08 | 2.2E-05 | 0.0034 | 2.20 | 0.0139 | 0.2033 |
| *IFRD2* | 3 | 1 | 1 | 4.07 | 2.3E-05 | 0.0035 | 1.72 | 0.0430 | 0.3763 |
| *ASB16* | 17 | 3 | 1 | 4.04 | 2.7E-05 | 0.0038 | 1.74 | 0.0405 | 0.3691 |
| *SF3B1* | 2 | 10 | 2 | 3.95 | 3.9E-05 | 0.0048 | 1.73 | 0.0420 | 0.3744 |
| *PIGU* | 20 | 43 | 5 | 3.95 | 4.0E-05 | 0.0049 | 2.55 | 0.0054 | 0.1136 |
| *RPRD2* | 1 | 57 | 6 | 3.94 | 4.0E-05 | 0.0049 | 2.88 | 0.0020 | 0.0593 |
| *NT5DC2* | 3 | 5 | 1 | 3.93 | 4.2E-05 | 0.0051 | 2.63 | 0.0043 | 0.0983 |
| *TARS2* | 1 | 15 | 3 | 3.92 | 4.4E-05 | 0.0051 | 2.59 | 0.0048 | 0.1048 |
| *CHDH* | 3 | 23 | 5 | 3.91 | 4.5E-05 | 0.0052 | 1.82 | 0.0347 | 0.3407 |
| *JADE2* | 5 | 40 | 10 | 3.90 | 4.9E-05 | 0.0055 | 2.78 | 0.0027 | 0.0724 |
| *UBTF* | 17 | 4 | 1 | 3.81 | 0.0001 | 0.0070 | 2.50 | 0.0063 | 0.1263 |
| *PHF7* | 3 | 4 | 2 | 3.78 | 0.0001 | 0.0075 | 1.72 | 0.0428 | 0.3761 |
| *HSPE1-MOB4* | 2 | 9 | 2 | 3.77 | 0.0001 | 0.0075 | 1.80 | 0.0362 | 0.3461 |
| *MATN4* | 20 | 12 | 3 | 3.77 | 0.0001 | 0.0076 | 1.91 | 0.0284 | 0.3096 |
| *LRRC3C* | 17 | 1 | 1 | 3.76 | 0.0001 | 0.0077 | 2.38 | 0.0087 | 0.1532 |
| *KCNS1* | 20 | 6 | 2 | 3.69 | 0.0001 | 0.0091 | 2.31 | 0.0105 | 0.1731 |
| *TSNAXIP1* | 16 | 5 | 2 | 3.68 | 0.0001 | 0.0095 | 2.41 | 0.0080 | 0.1455 |
| *GALNT14* | 2 | 242 | 32 | 3.65 | 0.0001 | 0.0100 | 1.87 | 0.0308 | 0.3211 |
| *CDCA2* | 8 | 35 | 2 | 3.61 | 0.0002 | 0.0109 | 2.59 | 0.0048 | 0.1057 |
| *MACROD1* | 11 | 119 | 23 | 3.61 | 0.0002 | 0.0109 | 2.11 | 0.0176 | 0.2352 |
| *HSPD1* | 2 | 12 | 2 | 3.61 | 0.0002 | 0.0109 | 1.69 | 0.0451 | 0.3821 |
| *FKBP2* | 11 | 2 | 1 | 3.61 | 0.0002 | 0.0110 | 2.80 | 0.0025 | 0.0694 |
| *HDAC7* | 12 | 27 | 10 | 3.60 | 0.0002 | 0.0111 | 1.97 | 0.0245 | 0.2865 |
| *NKAPL* | 6 | 4 | 2 | 3.60 | 0.0002 | 0.0113 | 1.93 | 0.0268 | 0.2998 |
| *TMUB2* | 17 | 3 | 1 | 3.59 | 0.0002 | 0.0115 | 1.74 | 0.0411 | 0.3713 |
| *SLC4A10* | 2 | 253 | 19 | 3.58 | 0.0002 | 0.0117 | 1.80 | 0.0359 | 0.3447 |
| *PIGC* | 1 | 46 | 10 | 3.54 | 0.0002 | 0.0129 | 2.20 | 0.0139 | 0.2033 |
| *PI3* | 20 | 2 | 1 | 3.51 | 0.0002 | 0.0140 | 1.81 | 0.0350 | 0.3421 |
| *ZFYVE21* | 14 | 8 | 2 | 3.50 | 0.0002 | 0.0144 | 2.55 | 0.0054 | 0.1136 |
| *ACHE* | 7 | 1 | 1 | 3.47 | 0.0003 | 0.0152 | 1.71 | 0.0440 | 0.3793 |
| *APOPT1* | 14 | 10 | 2 | 3.46 | 0.0003 | 0.0156 | 2.42 | 0.0077 | 0.1425 |
| *PPP1R13B* | 14 | 51 | 3 | 3.43 | 0.0003 | 0.0170 | 1.71 | 0.0438 | 0.3784 |
| *REEP2* | 5 | 4 | 1 | 3.43 | 0.0003 | 0.0172 | 2.43 | 0.0075 | 0.1402 |
| *RANBP10* | 16 | 10 | 3 | 3.43 | 0.0003 | 0.0173 | 2.63 | 0.0042 | 0.0969 |
| *TXNDC15* | 5 | 3 | 2 | 3.41 | 0.0003 | 0.0180 | 1.89 | 0.0293 | 0.3139 |
| *GSS* | 20 | 11 | 3 | 3.38 | 0.0004 | 0.0196 | 2.38 | 0.0087 | 0.1530 |
| *MPDU1* | 17 | 3 | 2 | 3.38 | 0.0004 | 0.0196 | 1.79 | 0.0369 | 0.3493 |
| *MARK1* | 1 | 48 | 4 | 3.36 | 0.0004 | 0.0203 | 1.80 | 0.0356 | 0.3443 |
| *PSMB10* | 16 | 2 | 1 | 3.34 | 0.0004 | 0.0215 | 1.74 | 0.0410 | 0.3709 |
| *AK3* | 9 | 40 | 8 | 3.32 | 0.0004 | 0.0223 | 1.96 | 0.0253 | 0.2899 |
| *CTC-479C5.12* | 16 | 2 | 1 | 3.31 | 0.0005 | 0.0227 | 1.73 | 0.0419 | 0.3735 |
| *MARS2* | 2 | 1 | 1 | 3.31 | 0.0005 | 0.0230 | 2.37 | 0.0089 | 0.1539 |
| *ZSWIM6* | 5 | 80 | 8 | 3.30 | 0.0005 | 0.0232 | 2.35 | 0.0095 | 0.1621 |
| *SLC27A5* | 19 | 8 | 3 | 3.28 | 0.0005 | 0.0244 | 2.02 | 0.0218 | 0.2682 |
| *PRKD1* | 14 | 392 | 57 | 3.27 | 0.0005 | 0.0253 | 2.39 | 0.0085 | 0.1506 |
| *CYP2R1* | 11 | 6 | 2 | 3.26 | 0.0006 | 0.0257 | 2.14 | 0.0162 | 0.2242 |
| *ZNF101* | 19 | 7 | 2 | 3.24 | 0.0006 | 0.0269 | 2.90 | 0.0018 | 0.0560 |
| *KCNN2* | 5 | 89 | 15 | 3.23 | 0.0006 | 0.0275 | 2.02 | 0.0219 | 0.2689 |
| *NAGPA* | 16 | 9 | 3 | 3.23 | 0.0006 | 0.0275 | 2.56 | 0.0053 | 0.1126 |
| *GNRH1* | 8 | 2 | 1 | 3.23 | 0.0006 | 0.0275 | 1.75 | 0.0400 | 0.3664 |
| *ETF1* | 5 | 13 | 2 | 3.22 | 0.0006 | 0.0279 | 2.23 | 0.0129 | 0.1964 |
| *PDE3B* | 11 | 64 | 7 | 3.17 | 0.0008 | 0.0309 | 2.10 | 0.0179 | 0.2379 |
| *RTN4RL1* | 17 | 50 | 16 | 3.17 | 0.0008 | 0.0313 | 2.66 | 0.0039 | 0.0933 |
| *ARHGAP15* | 2 | 478 | 62 | 3.16 | 0.0008 | 0.0316 | 2.27 | 0.0115 | 0.1843 |
| *SPATA33* | 16 | 11 | 3 | 3.16 | 0.0008 | 0.0316 | 2.11 | 0.0173 | 0.2326 |
| *CSK* | 15 | 14 | 3 | 3.16 | 0.0008 | 0.0319 | 2.93 | 0.0017 | 0.0523 |
| *IQCH* | 15 | 106 | 10 | 3.16 | 0.0008 | 0.0319 | 2.45 | 0.0072 | 0.1361 |
| *ZNF516* | 18 | 100 | 20 | 3.14 | 0.0008 | 0.0325 | 2.39 | 0.0084 | 0.1497 |
| *HSD11B2* | 16 | 1 | 1 | 3.14 | 0.0009 | 0.0332 | 1.97 | 0.0245 | 0.2865 |
| *S100PBP* | 1 | 14 | 4 | 3.13 | 0.0009 | 0.0337 | 1.72 | 0.0427 | 0.3761 |
| *ATP6V0D1* | 16 | 12 | 3 | 3.10 | 0.0010 | 0.0354 | 1.90 | 0.0288 | 0.3116 |
| *ZBTB45* | 19 | 5 | 2 | 3.08 | 0.0010 | 0.0371 | 2.31 | 0.0104 | 0.1725 |
| *SRPK2* | 7 | 126 | 7 | 3.07 | 0.0011 | 0.0385 | 1.84 | 0.0332 | 0.3359 |
| *NTM* | 11 | 1082 | 144 | 3.06 | 0.0011 | 0.0397 | 2.69 | 0.0036 | 0.0877 |
| *CYP1A2* | 15 | 3 | 1 | 3.05 | 0.0011 | 0.0397 | 2.54 | 0.0056 | 0.1161 |
| *MYH15* | 3 | 128 | 19 | 3.04 | 0.0012 | 0.0409 | 2.11 | 0.0173 | 0.2328 |
| *HSPA9* | 5 | 11 | 3 | 3.03 | 0.0012 | 0.0415 | 2.47 | 0.0068 | 0.1331 |
| *CDK10* | 16 | 14 | 4 | 2.99 | 0.0014 | 0.0456 | 1.98 | 0.0241 | 0.2849 |
| *FAHD2B* | 2 | 3 | 1 | 2.99 | 0.0014 | 0.0457 | 2.42 | 0.0078 | 0.1425 |
| *PBXIP1* | 1 | 4 | 1 | 2.98 | 0.0014 | 0.0460 | 1.77 | 0.0385 | 0.3586 |
| *HSPE1* | 2 | 1 | 1 | 2.97 | 0.0015 | 0.0473 | 2.12 | 0.0170 | 0.2294 |
| *UGGT2* | 13 | 104 | 8 | 2.97 | 0.0015 | 0.0475 | 1.74 | 0.0410 | 0.3709 |
| *COQ10B* | 2 | 2 | 1 | 2.96 | 0.0015 | 0.0479 | 1.98 | 0.0238 | 0.2827 |
| *ECM1* | 1 | 6 | 2 | 2.95 | 0.0016 | 0.0489 | 2.45 | 0.0071 | 0.1360 |
| *UBE2M* | 19 | 2 | 1 | 2.95 | 0.0016 | 0.0491 | 2.67 | 0.0038 | 0.0901 |
| *ATP6V1G3* | 1 | 10 | 3 | 2.94 | 0.0016 | 0.0496 | 2.74 | 0.0031 | 0.0793 |
| *HYAL2* | 3 | 1 | 1 | 2.93 | 0.0017 | 0.0507 | 2.31 | 0.0104 | 0.1726 |
| *TUSC2* | 3 | 1 | 1 | 2.93 | 0.0017 | 0.0507 | 2.31 | 0.0104 | 0.1726 |
| *SLC10A7* | 4 | 166 | 14 | 2.92 | 0.0017 | 0.0511 | 2.03 | 0.0210 | 0.2634 |
| *MSI2* | 17 | 416 | 93 | 2.92 | 0.0017 | 0.0512 | 1.88 | 0.0300 | 0.3180 |
| *SNX8* | 7 | 26 | 6 | 2.92 | 0.0018 | 0.0512 | 1.73 | 0.0417 | 0.3732 |
| *UBE3C* | 7 | 90 | 9 | 2.90 | 0.0018 | 0.0528 | 3.32 | 0.0004 | 0.0219 |
| *KCTD13* | 16 | 10 | 2 | 2.90 | 0.0019 | 0.0536 | 3.55 | 0.0002 | 0.0123 |
| *DYM* | 18 | 191 | 16 | 2.90 | 0.0019 | 0.0537 | 2.31 | 0.0105 | 0.1731 |
| *INO80E* | 16 | 4 | 1 | 2.89 | 0.0019 | 0.0539 | 4.34 | 7.0E-06 | 0.0010 |
| *GS1-259H13.10* | 7 | 18 | 3 | 2.89 | 0.0019 | 0.0540 | 1.79 | 0.0366 | 0.3487 |
| *DNAJB4* | 1 | 10 | 3 | 2.88 | 0.0020 | 0.0546 | 4.85 | 6.1E-07 | 0.0001 |
| *NT5C2* | 10 | 54 | 6 | 2.88 | 0.0020 | 0.0546 | 4.24 | 1.1E-05 | 0.0014 |
| *ATP2B2* | 3 | 444 | 77 | 2.86 | 0.0021 | 0.0566 | 1.92 | 0.0275 | 0.3034 |
| *IFT57* | 3 | 84 | 4 | 2.86 | 0.0021 | 0.0577 | 2.32 | 0.0101 | 0.1685 |
| *MAPK3* | 16 | 2 | 1 | 2.85 | 0.0022 | 0.0578 | 2.65 | 0.0040 | 0.0942 |
| *RORB* | 9 | 161 | 22 | 2.84 | 0.0023 | 0.0587 | 1.74 | 0.0407 | 0.3693 |
| *TCTN1* | 12 | 8 | 2 | 2.81 | 0.0025 | 0.0624 | 3.56 | 0.0002 | 0.0123 |
| *ZBTB38* | 3 | 72 | 10 | 2.80 | 0.0026 | 0.0636 | 2.23 | 0.0128 | 0.1942 |
| *MMEL1* | 1 | 23 | 2 | 2.80 | 0.0026 | 0.0636 | 2.07 | 0.0194 | 0.2513 |
| *TUBA1A* | 12 | 1 | 1 | 2.79 | 0.0026 | 0.0640 | 3.07 | 0.0011 | 0.0398 |
| *ZKSCAN5* | 7 | 9 | 2 | 2.79 | 0.0026 | 0.0644 | 1.90 | 0.0287 | 0.3115 |
| *ADCY9* | 16 | 122 | 33 | 2.78 | 0.0027 | 0.0653 | 5.28 | 6.4E-08 | 1.8E-05 |
| *PRPF3* | 1 | 13 | 3 | 2.78 | 0.0028 | 0.0662 | 3.01 | 0.0013 | 0.0456 |
| *ABCA3* | 16 | 24 | 6 | 2.78 | 0.0027 | 0.0662 | 1.97 | 0.0242 | 0.2853 |
| *HCAR1* | 12 | 32 | 7 | 2.77 | 0.0028 | 0.0669 | 2.24 | 0.0127 | 0.1937 |
| *ZNF789* | 7 | 4 | 2 | 2.77 | 0.0028 | 0.0669 | 1.89 | 0.0296 | 0.3163 |
| *ASAP2* | 2 | 146 | 11 | 2.77 | 0.0028 | 0.0669 | 1.70 | 0.0441 | 0.3794 |
| *AS3MT* | 10 | 19 | 5 | 2.76 | 0.0029 | 0.0673 | 4.52 | 3.1E-06 | 0.0005 |
| *CADPS* | 3 | 554 | 74 | 2.76 | 0.0029 | 0.0679 | 2.98 | 0.0014 | 0.0474 |
| *CIB3* | 19 | 9 | 2 | 2.75 | 0.0030 | 0.0691 | 2.86 | 0.0021 | 0.0618 |
| *RLTPR* | 16 | 2 | 1 | 2.74 | 0.0031 | 0.0706 | 2.54 | 0.0056 | 0.1161 |
| *PRPF39* | 14 | 5 | 1 | 2.74 | 0.0031 | 0.0707 | 1.65 | 0.0499 | 0.4065 |
| *SLTM* | 15 | 22 | 3 | 2.73 | 0.0031 | 0.0711 | 2.05 | 0.0203 | 0.2577 |
| *PGBD1* | 6 | 20 | 5 | 2.72 | 0.0032 | 0.0727 | 1.83 | 0.0338 | 0.3381 |
| *ZDHHC1* | 16 | 6 | 2 | 2.72 | 0.0033 | 0.0729 | 1.94 | 0.0262 | 0.2965 |
| *ZNF655* | 7 | 7 | 2 | 2.70 | 0.0034 | 0.0750 | 1.97 | 0.0244 | 0.2863 |
| *PBX4* | 19 | 13 | 4 | 2.70 | 0.0035 | 0.0758 | 2.98 | 0.0014 | 0.0480 |
| *CACNA1D* | 3 | 276 | 39 | 2.69 | 0.0036 | 0.0769 | 3.54 | 0.0002 | 0.0126 |
| *FAM53C* | 5 | 15 | 4 | 2.69 | 0.0036 | 0.0771 | 2.70 | 0.0035 | 0.0862 |
| *FAM200A* | 7 | 1 | 1 | 2.69 | 0.0036 | 0.0771 | 1.82 | 0.0342 | 0.3388 |
| *LRRC36* | 16 | 19 | 3 | 2.69 | 0.0036 | 0.0771 | 1.80 | 0.0359 | 0.3447 |
| *SLC35F1* | 6 | 319 | 34 | 2.68 | 0.0037 | 0.0786 | 3.40 | 0.0003 | 0.0181 |
| *KLC1* | 14 | 49 | 5 | 2.68 | 0.0037 | 0.0786 | 3.13 | 0.0009 | 0.0353 |
| *DDX19A* | 16 | 5 | 1 | 2.67 | 0.0038 | 0.0810 | 2.62 | 0.0043 | 0.0990 |
| *TRIM28* | 19 | 2 | 1 | 2.65 | 0.0040 | 0.0832 | 2.56 | 0.0053 | 0.1127 |
| *RGS17* | 6 | 130 | 15 | 2.65 | 0.0041 | 0.0833 | 3.62 | 0.0001 | 0.0101 |
| *RP11-73M18.2* | 14 | 37 | 4 | 2.64 | 0.0041 | 0.0842 | 3.23 | 0.0006 | 0.0269 |
| *CATSPER3* | 5 | 22 | 5 | 2.64 | 0.0042 | 0.0847 | 2.01 | 0.0222 | 0.2706 |
| *PSMA5* | 1 | 14 | 2 | 2.64 | 0.0042 | 0.0848 | 2.38 | 0.0087 | 0.1530 |
| *CNOT1* | 16 | 59 | 6 | 2.63 | 0.0042 | 0.0850 | 2.37 | 0.0088 | 0.1537 |
| *YARS* | 1 | 11 | 3 | 2.63 | 0.0042 | 0.0850 | 1.85 | 0.0320 | 0.3295 |
| *WBP1L* | 10 | 63 | 10 | 2.63 | 0.0043 | 0.0854 | 2.64 | 0.0041 | 0.0955 |
| *POMC* | 2 | 6 | 3 | 2.63 | 0.0043 | 0.0857 | 4.26 | 1.0E-05 | 0.0014 |
| *PPP1R14B* | 11 | 2 | 1 | 2.63 | 0.0043 | 0.0859 | 1.65 | 0.0498 | 0.4065 |
| *AP000295.9* | 21 | 21 | 6 | 2.63 | 0.0043 | 0.0860 | 1.70 | 0.0449 | 0.3819 |
| *ZCCHC7* | 9 | 94 | 8 | 2.62 | 0.0044 | 0.0865 | 3.31 | 0.0005 | 0.0225 |
| *ZNF839* | 14 | 12 | 4 | 2.62 | 0.0044 | 0.0875 | 1.69 | 0.0458 | 0.3848 |
| *AKT3* | 1 | 117 | 8 | 2.61 | 0.0045 | 0.0887 | 3.46 | 0.0003 | 0.0156 |
| *LFNG* | 7 | 4 | 2 | 2.61 | 0.0045 | 0.0887 | 1.97 | 0.0245 | 0.2865 |
| *RNPS1* | 16 | 4 | 1 | 2.60 | 0.0046 | 0.0895 | 1.83 | 0.0335 | 0.3372 |
| *FUT2* | 19 | 12 | 1 | 2.60 | 0.0047 | 0.0904 | 2.26 | 0.0119 | 0.1874 |
| *IFT81* | 12 | 18 | 5 | 2.59 | 0.0047 | 0.0905 | 3.10 | 0.0010 | 0.0376 |
| *RBM25* | 14 | 28 | 7 | 2.59 | 0.0049 | 0.0922 | 1.70 | 0.0445 | 0.3805 |
| *SDPR* | 2 | 7 | 2 | 2.57 | 0.0050 | 0.0945 | 1.92 | 0.0276 | 0.3036 |
| *TWF2* | 3 | 5 | 2 | 2.56 | 0.0052 | 0.0967 | 1.65 | 0.0498 | 0.4065 |
| *DCST2* | 1 | 4 | 2 | 2.55 | 0.0054 | 0.0988 | 2.42 | 0.0077 | 0.1425 |
| *DZIP3* | 3 | 41 | 6 | 2.55 | 0.0054 | 0.0988 | 1.87 | 0.0306 | 0.3205 |
| *GFOD2* | 16 | 4 | 2 | 2.54 | 0.0055 | 0.0994 | 1.73 | 0.0418 | 0.3735 |
| *POU6F1* | 12 | 16 | 5 | 2.54 | 0.0055 | 0.0996 | 1.82 | 0.0345 | 0.3404 |
| *AMN* | 14 | 4 | 2 | 2.53 | 0.0056 | 0.1008 | 1.94 | 0.0260 | 0.2960 |
| *TUBA1B* | 12 | 2 | 1 | 2.53 | 0.0058 | 0.1021 | 2.96 | 0.0015 | 0.0493 |
| *SIGLECL1* | 19 | 22 | 4 | 2.52 | 0.0058 | 0.1030 | 1.88 | 0.0300 | 0.3182 |
| *GGNBP1* | 6 | 8 | 3 | 2.52 | 0.0059 | 0.1033 | 1.85 | 0.0325 | 0.3316 |
| *NPEPPS* | 17 | 11 | 2 | 2.52 | 0.0059 | 0.1038 | 2.60 | 0.0047 | 0.1039 |
| *KPNB1* | 17 | 9 | 2 | 2.51 | 0.0060 | 0.1051 | 2.84 | 0.0023 | 0.0651 |
| *SKIDA1* | 10 | 2 | 1 | 2.51 | 0.0061 | 0.1055 | 3.03 | 0.0012 | 0.0434 |
| *FKBP3* | 14 | 4 | 1 | 2.49 | 0.0065 | 0.1088 | 1.91 | 0.0281 | 0.3082 |
| *MRPS21* | 1 | 12 | 4 | 2.47 | 0.0068 | 0.1125 | 2.71 | 0.0034 | 0.0842 |
| *YPEL1* | 22 | 21 | 6 | 2.47 | 0.0068 | 0.1126 | 2.00 | 0.0230 | 0.2768 |
| *ANKRD36* | 2 | 7 | 2 | 2.46 | 0.0069 | 0.1128 | 2.41 | 0.0079 | 0.1447 |
| *RP11-321N4.5* | 6 | 16 | 4 | 2.46 | 0.0069 | 0.1128 | 2.06 | 0.0196 | 0.2527 |
| *ZNF512* | 2 | 31 | 3 | 2.46 | 0.0070 | 0.1140 | 2.43 | 0.0076 | 0.1414 |
| *BAG5* | 14 | 4 | 2 | 2.46 | 0.0070 | 0.1143 | 3.62 | 0.0001 | 0.0103 |
| *ACD* | 16 | 1 | 1 | 2.45 | 0.0071 | 0.1157 | 2.51 | 0.0060 | 0.1216 |
| *GIPR* | 19 | 3 | 2 | 2.44 | 0.0074 | 0.1185 | 6.91 | 2.4E-12 | 2.5E-09 |
| *ETFB* | 19 | 13 | 3 | 2.44 | 0.0074 | 0.1185 | 2.69 | 0.0036 | 0.0870 |
| *RABGEF1* | 7 | 53 | 8 | 2.44 | 0.0074 | 0.1185 | 1.92 | 0.0273 | 0.3030 |
| *GMIP* | 19 | 4 | 2 | 2.43 | 0.0075 | 0.1189 | 2.02 | 0.0216 | 0.2672 |
| *CDC25C* | 5 | 27 | 5 | 2.43 | 0.0075 | 0.1190 | 3.76 | 0.0001 | 0.0071 |
| *QKI* | 6 | 126 | 10 | 2.43 | 0.0076 | 0.1196 | 1.96 | 0.0250 | 0.2885 |
| *RTF1* | 15 | 17 | 2 | 2.43 | 0.0076 | 0.1201 | 2.22 | 0.0133 | 0.1992 |
| *PSKH1* | 16 | 3 | 2 | 2.42 | 0.0078 | 0.1216 | 3.26 | 0.0006 | 0.0253 |
| *SNX14* | 6 | 42 | 7 | 2.40 | 0.0081 | 0.1250 | 1.83 | 0.0334 | 0.3372 |
| *AK5* | 1 | 281 | 42 | 2.40 | 0.0081 | 0.1251 | 5.15 | 1.3E-07 | 3.4E-05 |
| *KLF16* | 19 | 5 | 1 | 2.40 | 0.0081 | 0.1251 | 3.87 | 0.0001 | 0.0050 |
| *NEK7* | 1 | 106 | 12 | 2.39 | 0.0084 | 0.1271 | 1.87 | 0.0305 | 0.3205 |
| *CASC10* | 10 | 2 | 1 | 2.38 | 0.0086 | 0.1289 | 2.42 | 0.0078 | 0.1426 |
| *CLDN11* | 3 | 280 | 39 | 2.38 | 0.0086 | 0.1292 | 1.70 | 0.0442 | 0.3794 |
| *NSUN2* | 5 | 46 | 6 | 2.38 | 0.0086 | 0.1294 | 2.75 | 0.0030 | 0.0780 |
| *PPTC7* | 12 | 13 | 4 | 2.38 | 0.0088 | 0.1303 | 3.29 | 0.0005 | 0.0235 |
| *CCNT2* | 2 | 22 | 4 | 2.38 | 0.0088 | 0.1303 | 2.80 | 0.0025 | 0.0692 |
| *KIAA1522* | 1 | 8 | 3 | 2.37 | 0.0088 | 0.1303 | 1.96 | 0.0249 | 0.2880 |
| *CSAD* | 12 | 7 | 2 | 2.37 | 0.0088 | 0.1303 | 1.93 | 0.0268 | 0.2998 |
| *ARHGEF25* | 12 | 5 | 2 | 2.37 | 0.0088 | 0.1305 | 2.46 | 0.0070 | 0.1351 |
| *PHF14* | 7 | 108 | 8 | 2.37 | 0.0088 | 0.1305 | 1.78 | 0.0376 | 0.3532 |
| *NRXN2* | 11 | 39 | 4 | 2.36 | 0.0091 | 0.1332 | 2.83 | 0.0024 | 0.0668 |
| *OASL* | 12 | 13 | 5 | 2.36 | 0.0091 | 0.1332 | 1.76 | 0.0392 | 0.3629 |
| *REPIN1* | 7 | 6 | 1 | 2.36 | 0.0093 | 0.1342 | 2.59 | 0.0049 | 0.1059 |
| *ZFYVE26* | 14 | 82 | 9 | 2.35 | 0.0095 | 0.1358 | 2.11 | 0.0176 | 0.2356 |
| *GTF3C2* | 2 | 7 | 1 | 2.34 | 0.0095 | 0.1359 | 2.47 | 0.0068 | 0.1324 |
| *CAD* | 2 | 23 | 5 | 2.34 | 0.0097 | 0.1366 | 1.76 | 0.0391 | 0.3624 |
| *PPL* | 16 | 49 | 13 | 2.33 | 0.0098 | 0.1379 | 3.30 | 0.0005 | 0.0230 |
| *KCTD7* | 7 | 82 | 9 | 2.31 | 0.0105 | 0.1434 | 1.75 | 0.0397 | 0.3648 |
| *CCP110* | 16 | 26 | 6 | 2.30 | 0.0106 | 0.1445 | 2.25 | 0.0122 | 0.1894 |
| *M6PR* | 12 | 9 | 3 | 2.30 | 0.0108 | 0.1471 | 1.71 | 0.0438 | 0.3784 |
| *AC109829.1* | 2 | 5 | 1 | 2.29 | 0.0109 | 0.1482 | 2.66 | 0.0039 | 0.0924 |
| *ZNF513* | 2 | 1 | 1 | 2.29 | 0.0109 | 0.1483 | 1.66 | 0.0484 | 0.3992 |
| *KCNK3* | 2 | 20 | 3 | 2.29 | 0.0110 | 0.1487 | 5.01 | 2.7E-07 | 0.0001 |
| *RPS6KA5* | 14 | 96 | 9 | 2.29 | 0.0110 | 0.1487 | 2.46 | 0.0070 | 0.1351 |
| *NRXN1* | 2 | 1156 | 93 | 2.29 | 0.0111 | 0.1488 | 1.77 | 0.0383 | 0.3574 |
| *ATP2A2* | 12 | 12 | 4 | 2.28 | 0.0112 | 0.1495 | 2.52 | 0.0059 | 0.1200 |
| *DAGLA* | 11 | 31 | 7 | 2.28 | 0.0114 | 0.1507 | 2.00 | 0.0226 | 0.2732 |
| *CPSF6* | 12 | 17 | 4 | 2.28 | 0.0114 | 0.1508 | 1.85 | 0.0325 | 0.3317 |
| *SLC7A6* | 16 | 11 | 2 | 2.27 | 0.0115 | 0.1519 | 3.96 | 3.8E-05 | 0.0039 |
| *PPM1G* | 2 | 6 | 1 | 2.27 | 0.0116 | 0.1526 | 1.90 | 0.0286 | 0.3110 |
| *REXO1* | 19 | 13 | 3 | 2.27 | 0.0117 | 0.1531 | 3.66 | 0.0001 | 0.0092 |
| *MRAS* | 3 | 40 | 4 | 2.26 | 0.0119 | 0.1546 | 3.59 | 0.0002 | 0.0112 |
| *ZDHHC21* | 9 | 111 | 17 | 2.25 | 0.0122 | 0.1563 | 1.94 | 0.0260 | 0.2960 |
| *AARS* | 16 | 3 | 2 | 2.25 | 0.0123 | 0.1571 | 2.10 | 0.0179 | 0.2373 |
| *PLA2G3* | 22 | 7 | 4 | 2.24 | 0.0126 | 0.1586 | 2.05 | 0.0201 | 0.2567 |
| *DGKI* | 7 | 262 | 33 | 2.24 | 0.0126 | 0.1590 | 1.88 | 0.0301 | 0.3185 |
| *RTN4* | 2 | 126 | 16 | 2.24 | 0.0127 | 0.1592 | 1.92 | 0.0273 | 0.3030 |
| *RSRC1* | 3 | 277 | 11 | 2.23 | 0.0129 | 0.1598 | 2.92 | 0.0018 | 0.0545 |
| *MST1R* | 3 | 6 | 2 | 2.23 | 0.0130 | 0.1601 | 4.01 | 3.0E-05 | 0.0032 |
| *CNNM2* | 10 | 120 | 9 | 2.23 | 0.0130 | 0.1601 | 3.22 | 0.0006 | 0.0279 |
| *TSC2* | 16 | 13 | 6 | 2.23 | 0.0130 | 0.1601 | 2.52 | 0.0059 | 0.1200 |
| *SPATA18* | 4 | 16 | 4 | 2.22 | 0.0131 | 0.1612 | 2.70 | 0.0035 | 0.0862 |
| *SUFU* | 10 | 87 | 5 | 2.22 | 0.0132 | 0.1612 | 2.94 | 0.0017 | 0.0516 |
| *SLC4A1AP* | 2 | 4 | 2 | 2.22 | 0.0132 | 0.1612 | 1.70 | 0.0446 | 0.3807 |
| *RASIP1* | 19 | 7 | 2 | 2.21 | 0.0135 | 0.1629 | 2.24 | 0.0124 | 0.1912 |
| *G6PC3* | 17 | 2 | 1 | 2.20 | 0.0139 | 0.1667 | 1.68 | 0.0465 | 0.3891 |
| *ARL3* | 10 | 12 | 3 | 2.19 | 0.0141 | 0.1682 | 4.26 | 1.0E-05 | 0.0013 |
| *EPB41L1* | 20 | 36 | 5 | 2.19 | 0.0141 | 0.1685 | 2.26 | 0.0121 | 0.1883 |
| *ESRRA* | 11 | 3 | 1 | 2.19 | 0.0142 | 0.1687 | 2.21 | 0.0136 | 0.2012 |
| *TMEM248* | 7 | 20 | 5 | 2.19 | 0.0142 | 0.1687 | 2.02 | 0.0217 | 0.2676 |
| *MARK3* | 14 | 72 | 6 | 2.19 | 0.0143 | 0.1690 | 3.58 | 0.0002 | 0.0116 |
| *EIF4G1* | 3 | 5 | 2 | 2.18 | 0.0147 | 0.1714 | 3.01 | 0.0013 | 0.0454 |
| *NUTF2* | 16 | 5 | 2 | 2.18 | 0.0147 | 0.1714 | 1.68 | 0.0468 | 0.3905 |
| *BTBD2* | 19 | 15 | 7 | 2.18 | 0.0148 | 0.1718 | 4.25 | 1.1E-05 | 0.0014 |
| *OLA1* | 2 | 148 | 10 | 2.18 | 0.0148 | 0.1718 | 2.89 | 0.0019 | 0.0577 |
| *EIF2B4* | 2 | 3 | 1 | 2.18 | 0.0148 | 0.1718 | 2.25 | 0.0122 | 0.1900 |
| *AC087239.1* | 12 | 11 | 5 | 2.18 | 0.0148 | 0.1718 | 1.86 | 0.0313 | 0.3245 |
| *ZNF790* | 19 | 11 | 2 | 2.17 | 0.0149 | 0.1724 | 2.03 | 0.0214 | 0.2657 |
| *TTC34* | 1 | 4 | 1 | 2.17 | 0.0150 | 0.1732 | 3.11 | 0.0009 | 0.0366 |
| *IZUMO1* | 19 | 13 | 1 | 2.16 | 0.0154 | 0.1761 | 1.74 | 0.0410 | 0.3709 |
| *LTK* | 15 | 5 | 3 | 2.16 | 0.0154 | 0.1762 | 2.12 | 0.0172 | 0.2322 |
| *MAPK7* | 17 | 1 | 1 | 2.16 | 0.0154 | 0.1762 | 1.69 | 0.0455 | 0.3839 |
| *RP11-529K1.3* | 16 | 4 | 2 | 2.16 | 0.0156 | 0.1770 | 2.44 | 0.0073 | 0.1378 |
| *PIP4K2C* | 12 | 3 | 2 | 2.16 | 0.0156 | 0.1770 | 1.65 | 0.0499 | 0.4065 |
| *PLEKHG5* | 1 | 17 | 6 | 2.14 | 0.0160 | 0.1800 | 2.04 | 0.0209 | 0.2626 |
| *ASF1B* | 19 | 3 | 1 | 2.14 | 0.0163 | 0.1818 | 1.94 | 0.0261 | 0.2960 |
| *ADAM29* | 4 | 116 | 14 | 2.14 | 0.0164 | 0.1824 | 1.67 | 0.0476 | 0.3953 |
| *MIS12* | 17 | 10 | 3 | 2.13 | 0.0164 | 0.1829 | 4.05 | 2.5E-05 | 0.0028 |
| *MLLT10* | 10 | 36 | 4 | 2.13 | 0.0165 | 0.1830 | 2.94 | 0.0016 | 0.0512 |
| *SYNCRIP* | 6 | 15 | 4 | 2.13 | 0.0165 | 0.1830 | 1.73 | 0.0416 | 0.3727 |
| *VCL* | 10 | 48 | 6 | 2.13 | 0.0167 | 0.1836 | 2.63 | 0.0043 | 0.0979 |
| *ANKRD31* | 5 | 81 | 6 | 2.13 | 0.0166 | 0.1836 | 1.87 | 0.0305 | 0.3205 |
| *HIVEP3* | 1 | 382 | 37 | 2.13 | 0.0167 | 0.1836 | 3.61 | 0.0002 | 0.0106 |
| *TNS4* | 17 | 23 | 7 | 2.13 | 0.0168 | 0.1837 | 2.69 | 0.0036 | 0.0870 |
| *KRTCAP3* | 2 | 1 | 1 | 2.13 | 0.0168 | 0.1837 | 2.17 | 0.0149 | 0.2136 |
| *PEPD* | 19 | 121 | 18 | 2.12 | 0.0171 | 0.1861 | 2.47 | 0.0067 | 0.1313 |
| *NTHL1* | 16 | 3 | 1 | 2.12 | 0.0172 | 0.1865 | 3.42 | 0.0003 | 0.0170 |
| *C1orf54* | 1 | 7 | 3 | 2.11 | 0.0172 | 0.1869 | 2.62 | 0.0044 | 0.0990 |
| *TUFM* | 16 | 2 | 1 | 2.11 | 0.0173 | 0.1875 | 7.29 | 1.5E-13 | 2.4E-10 |
| *FANCL* | 2 | 39 | 8 | 2.11 | 0.0174 | 0.1879 | 2.06 | 0.0197 | 0.2535 |
| *ITPR3* | 6 | 75 | 15 | 2.11 | 0.0175 | 0.1883 | 2.14 | 0.0160 | 0.2227 |
| *KIF5A* | 12 | 12 | 3 | 2.11 | 0.0176 | 0.1890 | 2.59 | 0.0047 | 0.1046 |
| *SMPD3* | 16 | 71 | 7 | 2.10 | 0.0178 | 0.1904 | 4.19 | 1.4E-05 | 0.0017 |
| *SSBP3-AS1* | 1 | 1 | 1 | 2.10 | 0.0178 | 0.1904 | 2.31 | 0.0103 | 0.1711 |
| *CBX5* | 12 | 12 | 2 | 2.10 | 0.0178 | 0.1904 | 2.37 | 0.0089 | 0.1546 |
| *SCAMP2* | 15 | 12 | 4 | 2.10 | 0.0180 | 0.1918 | 2.48 | 0.0065 | 0.1296 |
| *UBIAD1* | 1 | 6 | 1 | 2.09 | 0.0183 | 0.1929 | 1.85 | 0.0323 | 0.3310 |
| *FXR2* | 17 | 9 | 3 | 2.09 | 0.0183 | 0.1931 | 2.33 | 0.0100 | 0.1681 |
| *EFR3B* | 2 | 57 | 10 | 2.08 | 0.0188 | 0.1956 | 5.44 | 2.7E-08 | 8.8E-06 |
| *SLC41A1* | 1 | 16 | 5 | 2.08 | 0.0187 | 0.1956 | 3.92 | 4.4E-05 | 0.0043 |
| *XRCC3* | 14 | 11 | 3 | 2.08 | 0.0188 | 0.1956 | 3.29 | 0.0005 | 0.0237 |
| *C10orf11* | 10 | 788 | 83 | 2.08 | 0.0188 | 0.1956 | 1.82 | 0.0345 | 0.3404 |
| *SRRM4* | 12 | 201 | 32 | 2.08 | 0.0189 | 0.1964 | 1.87 | 0.0306 | 0.3205 |
| *MFN2* | 1 | 13 | 3 | 2.07 | 0.0191 | 0.1976 | 2.49 | 0.0064 | 0.1271 |
| *MAP2K1* | 15 | 60 | 9 | 2.07 | 0.0191 | 0.1976 | 2.16 | 0.0154 | 0.2177 |
| *PPIL2* | 22 | 38 | 5 | 2.07 | 0.0192 | 0.1976 | 2.08 | 0.0190 | 0.2480 |
| *UCHL1* | 4 | 10 | 3 | 2.07 | 0.0193 | 0.1983 | 1.95 | 0.0254 | 0.2908 |
| *ZWILCH* | 15 | 16 | 4 | 2.07 | 0.0194 | 0.1989 | 3.30 | 0.0005 | 0.0232 |
| *PPM1A* | 14 | 30 | 2 | 2.07 | 0.0195 | 0.1993 | 2.05 | 0.0203 | 0.2576 |
| *BNIPL* | 1 | 6 | 2 | 2.06 | 0.0195 | 0.1996 | 3.63 | 0.0001 | 0.0099 |
| *RYBP* | 3 | 19 | 5 | 2.06 | 0.0197 | 0.2003 | 2.45 | 0.0071 | 0.1351 |
| *HYAL3* | 3 | 2 | 1 | 2.06 | 0.0198 | 0.2003 | 1.72 | 0.0429 | 0.3761 |
| *POFUT2* | 21 | 12 | 2 | 2.05 | 0.0200 | 0.2016 | 2.27 | 0.0117 | 0.1858 |
| *ZNHIT3* | 17 | 4 | 2 | 2.05 | 0.0200 | 0.2018 | 4.46 | 4.1E-06 | 0.0006 |
| *ST3GAL2* | 16 | 5 | 2 | 2.05 | 0.0201 | 0.2023 | 2.49 | 0.0063 | 0.1270 |
| *AP3M1* | 10 | 20 | 3 | 2.05 | 0.0202 | 0.2032 | 2.55 | 0.0054 | 0.1136 |
| *FAM19A2* | 12 | 522 | 62 | 2.04 | 0.0205 | 0.2049 | 2.07 | 0.0194 | 0.2513 |
| *SLC1A3* | 5 | 78 | 21 | 2.04 | 0.0208 | 0.2068 | 4.46 | 4.1E-06 | 0.0006 |
| *ITGAL* | 16 | 24 | 9 | 2.04 | 0.0208 | 0.2068 | 2.57 | 0.0051 | 0.1097 |
| *IFT172* | 2 | 11 | 2 | 2.04 | 0.0209 | 0.2068 | 2.20 | 0.0137 | 0.2025 |
| *RPTOR* | 17 | 470 | 50 | 2.03 | 0.0210 | 0.2068 | 3.89 | 0.0001 | 0.0048 |
| *C1orf51* | 1 | 3 | 2 | 2.04 | 0.0209 | 0.2068 | 2.62 | 0.0044 | 0.0990 |
| *C5orf49* | 5 | 16 | 4 | 2.03 | 0.0210 | 0.2068 | 1.97 | 0.0242 | 0.2850 |
| *PPAPDC2* | 9 | 3 | 2 | 2.03 | 0.0211 | 0.2077 | 1.70 | 0.0443 | 0.3798 |
| *NRBP1* | 2 | 4 | 1 | 2.03 | 0.0213 | 0.2086 | 2.04 | 0.0205 | 0.2592 |
| *ATRAID* | 2 | 3 | 2 | 2.03 | 0.0214 | 0.2092 | 1.96 | 0.0251 | 0.2886 |
| *CLN3* | 16 | 1 | 1 | 2.02 | 0.0215 | 0.2094 | 8.51 | 8.5E-18 | 3.7E-14 |
| *SH2B1* | 16 | 8 | 1 | 2.03 | 0.0214 | 0.2094 | 7.03 | 1.1E-12 | 1.2E-09 |
| *ATXN2L* | 16 | 5 | 1 | 2.02 | 0.0216 | 0.2106 | 7.29 | 1.5E-13 | 2.4E-10 |
| *ZNF280D* | 15 | 130 | 12 | 2.01 | 0.0220 | 0.2128 | 1.71 | 0.0437 | 0.3781 |
| *TTC7B* | 14 | 274 | 44 | 2.01 | 0.0222 | 0.2143 | 2.39 | 0.0084 | 0.1499 |
| *TRMT61A* | 14 | 6 | 2 | 2.01 | 0.0224 | 0.2152 | 2.43 | 0.0075 | 0.1402 |
| *AAGAB* | 15 | 29 | 3 | 2.01 | 0.0224 | 0.2152 | 1.96 | 0.0251 | 0.2886 |
| *RP11-463C8.4* | 14 | 121 | 25 | 2.01 | 0.0225 | 0.2153 | 2.01 | 0.0222 | 0.2705 |
| *HNF4G* | 8 | 190 | 23 | 2.00 | 0.0225 | 0.2155 | 3.26 | 0.0006 | 0.0254 |
| *RAB7L1* | 1 | 12 | 3 | 2.00 | 0.0226 | 0.2160 | 3.93 | 4.2E-05 | 0.0042 |
| *POLR3C* | 1 | 6 | 2 | 2.00 | 0.0227 | 0.2161 | 1.88 | 0.0303 | 0.3199 |
| *NDUFAF1* | 15 | 3 | 1 | 2.00 | 0.0228 | 0.2168 | 2.21 | 0.0134 | 0.2009 |
| *SLC7A6OS* | 16 | 12 | 2 | 2.00 | 0.0230 | 0.2179 | 3.56 | 0.0002 | 0.0121 |
| *GNAT1* | 3 | 1 | 1 | 2.00 | 0.0230 | 0.2179 | 2.97 | 0.0015 | 0.0485 |
| *MBD2* | 18 | 35 | 5 | 1.99 | 0.0231 | 0.2182 | 2.54 | 0.0056 | 0.1161 |
| *FAT3* | 11 | 419 | 32 | 1.99 | 0.0234 | 0.2194 | 2.64 | 0.0042 | 0.0964 |
| *ALDOA* | 16 | 3 | 2 | 1.98 | 0.0237 | 0.2205 | 2.98 | 0.0014 | 0.0477 |
| *VANGL2* | 1 | 20 | 3 | 1.98 | 0.0238 | 0.2207 | 2.07 | 0.0194 | 0.2513 |
| *GPR135* | 14 | 27 | 3 | 1.98 | 0.0238 | 0.2208 | 1.93 | 0.0268 | 0.2995 |
| *PEBP4* | 8 | 231 | 60 | 1.97 | 0.0244 | 0.2241 | 1.74 | 0.0413 | 0.3718 |
| *C16orf62* | 16 | 117 | 20 | 1.96 | 0.0247 | 0.2255 | 2.71 | 0.0034 | 0.0848 |
| *CA14* | 1 | 1 | 1 | 1.96 | 0.0250 | 0.2269 | 2.04 | 0.0209 | 0.2626 |
| *SLC12A9* | 7 | 13 | 3 | 1.96 | 0.0250 | 0.2269 | 1.81 | 0.0355 | 0.3441 |
| *SSBP3* | 1 | 110 | 29 | 1.95 | 0.0256 | 0.2302 | 3.45 | 0.0003 | 0.0160 |
| *FAM212B* | 1 | 50 | 13 | 1.95 | 0.0257 | 0.2302 | 3.17 | 0.0008 | 0.0318 |
| *HERC4* | 10 | 58 | 6 | 1.95 | 0.0256 | 0.2302 | 2.29 | 0.0110 | 0.1797 |
| *ZNF668* | 16 | 7 | 2 | 1.94 | 0.0260 | 0.2321 | 4.75 | 1.0E-06 | 0.0002 |
| *PDGFRL* | 8 | 110 | 21 | 1.94 | 0.0261 | 0.2324 | 3.15 | 0.0008 | 0.0329 |
| *LINGO2* | 9 | 781 | 106 | 1.94 | 0.0263 | 0.2335 | 4.42 | 4.9E-06 | 0.0007 |
| *ALDH1L2* | 12 | 65 | 12 | 1.93 | 0.0265 | 0.2341 | 1.72 | 0.0431 | 0.3766 |
| *NOS1AP* | 1 | 388 | 46 | 1.93 | 0.0268 | 0.2355 | 3.38 | 0.0004 | 0.0189 |
| *RFWD2* | 1 | 135 | 14 | 1.93 | 0.0271 | 0.2364 | 2.86 | 0.0021 | 0.0618 |
| *FAM219B* | 15 | 3 | 1 | 1.93 | 0.0270 | 0.2364 | 2.12 | 0.0169 | 0.2285 |
| *SLC30A3* | 2 | 8 | 1 | 1.93 | 0.0271 | 0.2364 | 1.94 | 0.0261 | 0.2960 |
| *ZZZ3* | 1 | 45 | 6 | 1.92 | 0.0273 | 0.2371 | 3.01 | 0.0013 | 0.0452 |
| *NRXN3* | 14 | 1344 | 130 | 1.91 | 0.0280 | 0.2414 | 5.83 | 2.8E-09 | 1.0E-06 |
| *BBX* | 3 | 179 | 20 | 1.90 | 0.0287 | 0.2450 | 1.82 | 0.0345 | 0.3404 |
| *DNMT3A* | 2 | 41 | 10 | 1.90 | 0.0289 | 0.2458 | 3.81 | 0.0001 | 0.0060 |
| *AMIGO2* | 12 | 2 | 1 | 1.89 | 0.0292 | 0.2466 | 2.61 | 0.0046 | 0.1026 |
| *CCBL1* | 9 | 10 | 3 | 1.89 | 0.0292 | 0.2466 | 2.61 | 0.0046 | 0.1026 |
| *ATP8B3* | 19 | 19 | 7 | 1.89 | 0.0292 | 0.2466 | 2.29 | 0.0110 | 0.1789 |
| *TMX1* | 14 | 7 | 3 | 1.89 | 0.0292 | 0.2466 | 1.85 | 0.0321 | 0.3301 |
| *CDC37L1* | 9 | 26 | 3 | 1.89 | 0.0294 | 0.2473 | 3.74 | 0.0001 | 0.0074 |
| *VAX1* | 10 | 3 | 1 | 1.89 | 0.0294 | 0.2473 | 2.37 | 0.0088 | 0.1535 |
| *BLMH* | 17 | 20 | 5 | 1.89 | 0.0293 | 0.2473 | 1.95 | 0.0256 | 0.2922 |
| *MVP* | 16 | 9 | 1 | 1.88 | 0.0297 | 0.2488 | 2.86 | 0.0021 | 0.0629 |
| *PEAK1* | 15 | 109 | 10 | 1.88 | 0.0298 | 0.2490 | 1.98 | 0.0241 | 0.2849 |
| *LARS* | 5 | 61 | 2 | 1.88 | 0.0298 | 0.2490 | 1.75 | 0.0403 | 0.3679 |
| *DIS3L* | 15 | 20 | 6 | 1.87 | 0.0307 | 0.2538 | 1.97 | 0.0242 | 0.2853 |
| *AP1M1* | 19 | 28 | 3 | 1.87 | 0.0307 | 0.2539 | 3.14 | 0.0008 | 0.0337 |
| *GLTP* | 12 | 12 | 4 | 1.87 | 0.0308 | 0.2539 | 2.48 | 0.0065 | 0.1295 |
| *KLHL26* | 19 | 15 | 4 | 1.87 | 0.0308 | 0.2544 | 1.86 | 0.0313 | 0.3245 |
| *SIRT1* | 10 | 15 | 2 | 1.86 | 0.0312 | 0.2564 | 1.65 | 0.0495 | 0.4050 |
| *TNKS* | 8 | 162 | 11 | 1.86 | 0.0313 | 0.2568 | 2.04 | 0.0205 | 0.2592 |
| *CTD-2616J11.11* | 19 | 6 | 2 | 1.86 | 0.0314 | 0.2569 | 2.44 | 0.0073 | 0.1375 |
| *CLIP1* | 12 | 39 | 7 | 1.86 | 0.0317 | 0.2576 | 4.12 | 1.9E-05 | 0.0022 |
| *FOXO3* | 6 | 59 | 8 | 1.85 | 0.0319 | 0.2588 | 4.44 | 4.4E-06 | 0.0007 |
| *SHISA4* | 1 | 1 | 1 | 1.85 | 0.0325 | 0.2614 | 5.20 | 1.0E-07 | 2.7E-05 |
| *FHIT* | 3 | 1859 | 246 | 1.84 | 0.0326 | 0.2617 | 1.88 | 0.0302 | 0.3193 |
| *PRKACA* | 19 | 3 | 1 | 1.84 | 0.0328 | 0.2620 | 1.93 | 0.0267 | 0.2991 |
| *AKAP10* | 17 | 23 | 2 | 1.84 | 0.0329 | 0.2625 | 2.83 | 0.0023 | 0.0666 |
| *PRB1* | 12 | 15 | 3 | 1.84 | 0.0330 | 0.2625 | 1.87 | 0.0305 | 0.3205 |
| *LPPR1* | 9 | 326 | 37 | 1.84 | 0.0332 | 0.2638 | 4.07 | 2.4E-05 | 0.0026 |
| *HPCA* | 1 | 2 | 1 | 1.83 | 0.0334 | 0.2643 | 1.73 | 0.0415 | 0.3722 |
| *ZBTB43* | 9 | 7 | 2 | 1.83 | 0.0335 | 0.2646 | 2.29 | 0.0110 | 0.1789 |
| *PARD3* | 10 | 565 | 40 | 1.83 | 0.0336 | 0.2654 | 2.74 | 0.0030 | 0.0784 |
| *ULK3* | 15 | 7 | 2 | 1.83 | 0.0338 | 0.2657 | 2.78 | 0.0027 | 0.0731 |
| *MDH1* | 2 | 13 | 2 | 1.83 | 0.0337 | 0.2657 | 2.16 | 0.0153 | 0.2174 |
| *MLLT3* | 9 | 175 | 30 | 1.83 | 0.0337 | 0.2657 | 2.15 | 0.0158 | 0.2210 |
| *MAP2K5* | 15 | 191 | 17 | 1.83 | 0.0339 | 0.2661 | 6.47 | 5.0E-11 | 3.0E-08 |
| *DNAJC11* | 1 | 30 | 5 | 1.83 | 0.0339 | 0.2661 | 2.53 | 0.0058 | 0.1189 |
| *POU6F2* | 7 | 462 | 46 | 1.83 | 0.0339 | 0.2661 | 2.13 | 0.0167 | 0.2280 |
| *LPHN3* | 4 | 409 | 65 | 1.83 | 0.0339 | 0.2661 | 1.70 | 0.0442 | 0.3794 |
| *KIAA1598* | 10 | 48 | 4 | 1.82 | 0.0342 | 0.2666 | 3.52 | 0.0002 | 0.0132 |
| *LRRC61* | 7 | 10 | 1 | 1.82 | 0.0341 | 0.2666 | 2.70 | 0.0035 | 0.0859 |
| *ZNF345* | 19 | 23 | 4 | 1.82 | 0.0340 | 0.2666 | 1.88 | 0.0300 | 0.3180 |
| *PLAC8L1* | 5 | 14 | 1 | 1.82 | 0.0342 | 0.2666 | 1.77 | 0.0382 | 0.3572 |
| *SRRT* | 7 | 10 | 3 | 1.82 | 0.0345 | 0.2675 | 1.83 | 0.0339 | 0.3383 |
| *EFNA1* | 1 | 1 | 1 | 1.82 | 0.0347 | 0.2683 | 2.07 | 0.0192 | 0.2498 |
| *AXIN1* | 16 | 42 | 8 | 1.81 | 0.0350 | 0.2695 | 4.37 | 6.3E-06 | 0.0009 |
| *CRB1* | 1 | 120 | 17 | 1.81 | 0.0350 | 0.2695 | 2.57 | 0.0052 | 0.1106 |
| *DOC2A* | 16 | 9 | 1 | 1.81 | 0.0352 | 0.2697 | 4.36 | 6.5E-06 | 0.0009 |
| *COX5A* | 15 | 5 | 1 | 1.81 | 0.0352 | 0.2697 | 2.07 | 0.0193 | 0.2513 |
| *GRIK5* | 19 | 8 | 3 | 1.80 | 0.0356 | 0.2707 | 2.14 | 0.0160 | 0.2226 |
| *PTPRN2* | 7 | 671 | 125 | 1.80 | 0.0359 | 0.2718 | 2.28 | 0.0113 | 0.1825 |
| *SLC8A1* | 2 | 555 | 60 | 1.80 | 0.0362 | 0.2727 | 2.55 | 0.0053 | 0.1129 |
| *PRRT2* | 16 | 2 | 1 | 1.80 | 0.0362 | 0.2729 | 2.68 | 0.0037 | 0.0891 |
| *MON1A* | 3 | 4 | 1 | 1.79 | 0.0368 | 0.2752 | 2.43 | 0.0075 | 0.1402 |
| *SYT14* | 1 | 96 | 9 | 1.79 | 0.0370 | 0.2763 | 3.79 | 0.0001 | 0.0065 |
| *TUBA4A* | 2 | 18 | 3 | 1.79 | 0.0370 | 0.2763 | 2.48 | 0.0065 | 0.1291 |
| *WDPCP* | 2 | 374 | 12 | 1.79 | 0.0371 | 0.2765 | 3.01 | 0.0013 | 0.0456 |
| *IPO9* | 1 | 18 | 4 | 1.78 | 0.0373 | 0.2768 | 5.33 | 4.8E-08 | 1.4E-05 |
| *NFE2* | 12 | 1 | 1 | 1.78 | 0.0374 | 0.2773 | 2.74 | 0.0031 | 0.0788 |
| *HOXA7* | 7 | 3 | 1 | 1.78 | 0.0376 | 0.2781 | 2.39 | 0.0084 | 0.1497 |
| *RESP18* | 2 | 9 | 2 | 1.78 | 0.0377 | 0.2781 | 2.20 | 0.0140 | 0.2035 |
| *ZNF829* | 19 | 6 | 2 | 1.78 | 0.0376 | 0.2781 | 1.72 | 0.0423 | 0.3760 |
| *ZNF850* | 19 | 19 | 4 | 1.77 | 0.0381 | 0.2805 | 1.70 | 0.0450 | 0.3819 |
| *CAMKMT* | 2 | 305 | 15 | 1.77 | 0.0382 | 0.2807 | 3.30 | 0.0005 | 0.0232 |
| *ZFPM2* | 8 | 399 | 57 | 1.77 | 0.0382 | 0.2807 | 2.42 | 0.0077 | 0.1419 |
| *FAM65A* | 16 | 2 | 1 | 1.77 | 0.0382 | 0.2807 | 1.89 | 0.0296 | 0.3163 |
| *MYO15A* | 17 | 37 | 7 | 1.77 | 0.0385 | 0.2817 | 2.33 | 0.0098 | 0.1658 |
| *TMEM63C* | 14 | 157 | 31 | 1.77 | 0.0385 | 0.2817 | 1.80 | 0.0357 | 0.3445 |
| *PSEN1* | 14 | 38 | 8 | 1.76 | 0.0388 | 0.2823 | 2.16 | 0.0153 | 0.2174 |
| *ERP44* | 9 | 47 | 5 | 1.76 | 0.0390 | 0.2829 | 3.13 | 0.0009 | 0.0348 |
| *MPC1* | 6 | 12 | 2 | 1.75 | 0.0403 | 0.2896 | 2.32 | 0.0102 | 0.1694 |
| *CRTC1* | 19 | 26 | 6 | 1.74 | 0.0406 | 0.2907 | 2.49 | 0.0065 | 0.1287 |
| *TUBA1C* | 12 | 20 | 5 | 1.74 | 0.0410 | 0.2926 | 3.59 | 0.0002 | 0.0112 |
| *PLCG1* | 20 | 16 | 4 | 1.74 | 0.0414 | 0.2939 | 2.44 | 0.0073 | 0.1378 |
| *FSTL5* | 4 | 613 | 74 | 1.73 | 0.0415 | 0.2946 | 3.05 | 0.0011 | 0.0416 |
| *ARL15* | 5 | 437 | 57 | 1.73 | 0.0419 | 0.2956 | 1.67 | 0.0473 | 0.3936 |
| *SUPT7L* | 2 | 4 | 2 | 1.73 | 0.0419 | 0.2956 | 1.66 | 0.0486 | 0.4000 |
| *DERL2* | 17 | 19 | 5 | 1.73 | 0.0421 | 0.2967 | 4.16 | 1.6E-05 | 0.0019 |
| *GTF2A1L* | 2 | 136 | 24 | 1.73 | 0.0421 | 0.2967 | 1.71 | 0.0434 | 0.3768 |
| *RIC8B* | 12 | 49 | 6 | 1.72 | 0.0429 | 0.3002 | 1.77 | 0.0383 | 0.3574 |
| *ZBED6CL* | 7 | 1 | 1 | 1.72 | 0.0432 | 0.3012 | 2.73 | 0.0032 | 0.0802 |
| *FNDC4* | 2 | 2 | 1 | 1.71 | 0.0432 | 0.3012 | 2.13 | 0.0167 | 0.2278 |
| *CTNNAL1* | 9 | 64 | 8 | 1.72 | 0.0431 | 0.3012 | 1.82 | 0.0344 | 0.3402 |
| *CHRNB4* | 15 | 62 | 10 | 1.71 | 0.0433 | 0.3014 | 2.19 | 0.0142 | 0.2043 |
| *MAP3K19* | 2 | 45 | 8 | 1.71 | 0.0436 | 0.3020 | 3.29 | 0.0005 | 0.0232 |
| *BEND5* | 1 | 44 | 9 | 1.71 | 0.0438 | 0.3029 | 1.80 | 0.0358 | 0.3447 |
| *HNRNPA1* | 12 | 3 | 1 | 1.71 | 0.0438 | 0.3029 | 2.22 | 0.0131 | 0.1980 |
| *KLK7* | 19 | 5 | 2 | 1.71 | 0.0440 | 0.3034 | 1.67 | 0.0470 | 0.3920 |
| *PGPEP1* | 19 | 14 | 6 | 1.70 | 0.0441 | 0.3039 | 1.87 | 0.0305 | 0.3205 |
| *ITGB3* | 17 | 73 | 12 | 1.70 | 0.0445 | 0.3046 | 3.10 | 0.0010 | 0.0376 |
| *AC091801.1* | 7 | 48 | 11 | 1.70 | 0.0445 | 0.3046 | 2.21 | 0.0137 | 0.2024 |
| *PAGR1* | 16 | 4 | 1 | 1.70 | 0.0446 | 0.3049 | 2.52 | 0.0058 | 0.1198 |
| *DRD2* | 11 | 69 | 10 | 1.70 | 0.0448 | 0.3053 | 2.97 | 0.0015 | 0.0483 |
| *ISL1* | 5 | 2 | 1 | 1.70 | 0.0448 | 0.3053 | 1.82 | 0.0341 | 0.3383 |
| *INVS* | 9 | 126 | 7 | 1.69 | 0.0451 | 0.3060 | 2.64 | 0.0041 | 0.0957 |
| *LDHAL6B* | 15 | 5 | 2 | 1.69 | 0.0453 | 0.3068 | 2.70 | 0.0035 | 0.0859 |
| *RANBP1* | 22 | 4 | 2 | 1.69 | 0.0455 | 0.3073 | 1.70 | 0.0449 | 0.3819 |
| *LHCGR* | 2 | 157 | 29 | 1.69 | 0.0456 | 0.3075 | 1.90 | 0.0285 | 0.3106 |
| *CKMT1A* | 15 | 2 | 1 | 1.69 | 0.0459 | 0.3094 | 3.37 | 0.0004 | 0.0192 |
| *ATP2A1* | 16 | 6 | 2 | 1.68 | 0.0462 | 0.3100 | 7.42 | 5.7E-14 | 1.7E-10 |
| *SNX19* | 11 | 81 | 7 | 1.68 | 0.0463 | 0.3104 | 1.66 | 0.0487 | 0.4002 |
| *PAN3* | 13 | 72 | 9 | 1.68 | 0.0464 | 0.3107 | 1.80 | 0.0360 | 0.3455 |
| *GOLGA3* | 12 | 30 | 9 | 1.68 | 0.0466 | 0.3116 | 2.41 | 0.0081 | 0.1461 |
| *SPRYD3* | 12 | 11 | 3 | 1.68 | 0.0466 | 0.3117 | 2.16 | 0.0153 | 0.2174 |
| *VPRBP* | 3 | 10 | 3 | 1.68 | 0.0468 | 0.3126 | 2.02 | 0.0218 | 0.2680 |
| *ENO4* | 10 | 16 | 3 | 1.68 | 0.0470 | 0.3131 | 3.76 | 0.0001 | 0.0070 |
| *ULK4* | 3 | 478 | 31 | 1.67 | 0.0471 | 0.3132 | 1.72 | 0.0429 | 0.3761 |
| *SNTG1* | 8 | 694 | 41 | 1.67 | 0.0471 | 0.3132 | 1.65 | 0.0499 | 0.4065 |
| *CHRNA3* | 15 | 25 | 4 | 1.67 | 0.0474 | 0.3145 | 1.98 | 0.0239 | 0.2831 |
| *CUX2* | 12 | 80 | 12 | 1.67 | 0.0478 | 0.3159 | 2.32 | 0.0101 | 0.1685 |
| *OR10K1* | 1 | 1 | 1 | 1.67 | 0.0479 | 0.3162 | 1.75 | 0.0401 | 0.3668 |
| *DDX59* | 1 | 33 | 7 | 1.66 | 0.0482 | 0.3171 | 1.77 | 0.0386 | 0.3592 |
| *UPK3B* | 7 | 94 | 27 | 1.66 | 0.0485 | 0.3173 | 3.10 | 0.0010 | 0.0376 |
| *SOAT2* | 12 | 18 | 4 | 1.66 | 0.0485 | 0.3176 | 1.85 | 0.0321 | 0.3301 |
| *TP63* | 3 | 298 | 52 | 1.65 | 0.0490 | 0.3194 | 2.28 | 0.0113 | 0.1829 |
| *CDH11* | 16 | 152 | 16 | 1.65 | 0.0491 | 0.3195 | 2.75 | 0.0030 | 0.0782 |
| *RP11-1055B8.7* | 17 | 16 | 6 | 1.65 | 0.0492 | 0.3200 | 1.82 | 0.0341 | 0.3383 |
| *HKR1* | 19 | 23 | 4 | 1.65 | 0.0494 | 0.3207 | 1.88 | 0.0299 | 0.3180 |
| *PHF13* | 1 | 5 | 2 | 1.65 | 0.0500 | 0.3230 | 2.51 | 0.0060 | 0.1222 |

Abbreviations: BH, Benjamini–Hochberg procedure; BMI, body mass index; SNP, single nucleotide polymorphism

#SNPs: number of SNPs annotated to a specific gene in the data and not excluded based on internal MAGMA quality control; NPARAM: number of SNPs used by MAGMA in the model; Z: Z-statistics for the gene, based on its p-value.

Genes significant after multiple testing correction according to the BH procedure in both BD and BMI are reported in bold.
