# Supplementary material for: Evidence that genes involved in hedgehog signaling are associated with both bipolar disorder and high BMI
Source: Transl Psychiatry. 2019 Nov 21;9:315. doi: 10.1038/s41398-019-0652-x (PMC6872724; doi:10.1038/s41398-019-0652-x)
Supplement: Supplementary file 3 — Supplementary Table 2 [file 41398_2019_652_MOESM3_ESM.docx]

**Supplementary Table 2. Enrichment for genes associated with BMI, T2D or both phenotypes among genes associated with BD**

| **Comparison** | **Total number of genes** | **Genes associated with BD** | **Genes associated with the second phenotype** | **Expected number of overlapping genes** | **Observed number of overlapping genes** | **Enrichment** | **p** |
| --- | --- | --- | --- | --- | --- | --- | --- |
| **BD and BMI** | **17,455** | **579** | **549** | **18** | **52** | **2.86** | **9.4E-12** |
| **BD and T2D** | **17,455** | **579** | **173** | **6** | **12** | **2.09** | **0.01** |
| **BD and BMI or T2D** | **17,455** | **579** | **690** | **23** | **61** | **2.67** | **2.6E-12** |
| BD and BMI and T2D | 17,455 | 579 | 30 | 1 | 3 | 3.01 | 0.076 |

Abbreviations: BD, bipolar disorder; BMI, body mass index; T2D, type 2 diabetes.
