# Supplementary material for: Evidence that genes involved in hedgehog signaling are associated with both bipolar disorder and high BMI
Source: Transl Psychiatry. 2019 Nov 21;9:315. doi: 10.1038/s41398-019-0652-x (PMC6872724; doi:10.1038/s41398-019-0652-x)
Supplement: Supplementary file 4 — Supplementary Table 3 [file 41398_2019_652_MOESM4_ESM.docx]

**Supplementary Table 3. Genes associated with both bipolar disorder and T2D in the gene-based analysis conducted with MAGMA**

|  |  |  |  | **Bipolar disorder** | | | **Type 2 Diabetes** | | |
| --- | --- | --- | --- | --- | --- | --- | --- | --- | --- |
| **Gene** | **Chr** | **# SNPs** | **NPARAM** | **Z** | **p** | **adj p (BH)** | **Z** | **p** | **adj p (BH)** |
| ***FADS2*** | **11** | **35** | **6** | **5.47** | **2.28E-08** | **5.0E-05** | **4.23** | **1.1E-05** | **0.0039** |
| ***MYRF*** | **11** | **21** | **6** | **5.57** | **1.30E-08** | **5.0E-05** | **3.54** | **0.0002** | **0.0280** |
| ***TMEM258*** | **11** | **16** | **4** | **5.14** | **1.37E-07** | **0.0001** | **3.78** | **0.0001** | **0.0153** |
| ***PACS1*** | **11** | **66** | **7** | **5.12** | **1.49E-07** | **0.0002** | **3.46** | **0.0003** | **0.0334** |
| ***FADS1*** | **11** | **7** | **1** | **4.38** | **5.95E-06** | **0.0013** | **3.45** | **0.0003** | **0.0334** |
| ***STAB1*** | **3** | **14** | **4** | **4.08** | **2.27E-05** | **0.0034** | **3.37** | **0.0004** | **0.0424** |
| ***RAB1B*** | **11** | **3** | **1** | **3.70** | **0.0001084** | **0.0090** | **3.46** | **0.0003** | **0.0334** |
| ***TMEM151A*** | **11** | **3** | **2** | **3.65** | **0.000133** | **0.0101** | **3.32** | **0.0005** | **0.0478** |
| ***TAOK2*** | **16** | **11** | **1** | **3.37** | **0.0003798** | **0.0200** | **3.50** | **0.0002** | **0.0299** |
| ***SF3B2*** | **11** | **7** | **2** | **3.30** | **0.0004841** | **0.0234** | **3.30** | **0.0005** | **0.0496** |
| ***TMEM219*** | **16** | **3** | **1** | **3.21** | **0.0006672** | **0.0286** | **3.30** | **0.0005** | **0.0491** |
| ***PABPC4*** | **1** | **12** | **4** | **2.99** | **0.0014079** | **0.0457** | **4.18** | **1.4E-05** | **0.0045** |
| *ITIH3* | 3 | 7 | 2 | 5.51 | 1.77E-08 | 5.0E-05 | 1.69 | 0.0455 | 0.4264 |
| *HLF* | 17 | 55 | 11 | 5.36 | 4.17E-08 | 0.0001 | 2.47 | 0.0068 | 0.1910 |
| *NEK4* | 3 | 13 | 3 | 4.96 | 3.52E-07 | 0.0003 | 2.01 | 0.0222 | 0.3265 |
| *SPCS1* | 3 | 1 | 1 | 4.96 | 3.52E-07 | 0.0003 | 1.94 | 0.0260 | 0.3466 |
| *MAPK1* | 22 | 66 | 7 | 4.93 | 4.11E-07 | 0.0003 | 1.71 | 0.0433 | 0.4223 |
| *PBRM1* | 3 | 52 | 3 | 4.84 | 6.38E-07 | 0.0004 | 2.12 | 0.0169 | 0.2851 |
| *GNL3* | 3 | 7 | 2 | 4.84 | 6.59E-07 | 0.0004 | 2.06 | 0.0195 | 0.3069 |
| *RP11-1167A19.2* | 11 | 1 | 1 | 4.75 | 1.01E-06 | 0.0005 | 2.95 | 0.0016 | 0.0879 |
| *SMIM4* | 3 | 18 | 2 | 4.74 | 1.09E-06 | 0.0005 | 2.23 | 0.0128 | 0.2501 |
| *MAP1LC3A* | 20 | 2 | 1 | 4.64 | 1.75E-06 | 0.0008 | 2.66 | 0.0039 | 0.1401 |
| *ITIH1* | 3 | 16 | 2 | 4.65 | 1.69E-06 | 0.0008 | 1.94 | 0.0265 | 0.3483 |
| *TLR9* | 3 | 4 | 2 | 4.61 | 2.02E-06 | 0.0008 | 2.90 | 0.0019 | 0.0967 |
| *TLR9* | 3 | 8 | 3 | 4.56 | 2.54E-06 | 0.0008 | 2.67 | 0.0038 | 0.1392 |
| *RP11-343C2.9* | 16 | 11 | 3 | 4.57 | 2.49E-06 | 0.0008 | 2.28 | 0.0113 | 0.2343 |
| *RP11-343C2.7* | 16 | 11 | 3 | 4.57 | 2.49E-06 | 0.0008 | 2.28 | 0.0113 | 0.2343 |
| *SLC4A1* | 17 | 9 | 4 | 4.59 | 2.26E-06 | 0.0008 | 1.75 | 0.0403 | 0.4083 |
| *TMED6* | 16 | 6 | 3 | 4.57 | 2.41E-06 | 0.0008 | 1.69 | 0.0457 | 0.4273 |
| *GRIN2A* | 16 | 546 | 33 | 4.51 | 3.19E-06 | 0.0009 | 2.15 | 0.0157 | 0.2744 |
| *GOPC* | 6 | 281 | 31 | 4.49 | 3.53E-06 | 0.0010 | 2.27 | 0.0117 | 0.2382 |
| *GLT8D1* | 3 | 4 | 1 | 4.48 | 3.66E-06 | 0.0010 | 1.92 | 0.0273 | 0.3529 |
| *SFMBT1* | 3 | 60 | 7 | 4.49 | 3.54E-06 | 0.0010 | 1.81 | 0.0348 | 0.3879 |
| *DCBLD1* | 6 | 125 | 20 | 4.45 | 4.21E-06 | 0.0010 | 2.53 | 0.0057 | 0.1722 |
| *CDKN2C* | 1 | 3 | 1 | 4.30 | 8.71E-06 | 0.0017 | 2.98 | 0.0014 | 0.0864 |
| *NCOA6* | 20 | 42 | 4 | 4.27 | 9.63E-06 | 0.0018 | 2.67 | 0.0038 | 0.1392 |
| *KLC2* | 11 | 4 | 1 | 4.25 | 1.07E-05 | 0.0019 | 3.11 | 0.0009 | 0.0695 |
| *GGT7* | 20 | 8 | 2 | 4.22 | 1.24E-05 | 0.0021 | 2.89 | 0.0019 | 0.0987 |
| *COG8* | 16 | 8 | 3 | 4.20 | 1.31E-05 | 0.0022 | 2.38 | 0.0086 | 0.2080 |
| *FAF1* | 1 | 138 | 16 | 4.08 | 2.24E-05 | 0.0034 | 2.53 | 0.0057 | 0.1722 |
| *DYNLRB1* | 20 | 11 | 2 | 4.06 | 2.49E-05 | 0.0036 | 2.96 | 0.0015 | 0.0873 |
| *PROZ* | 13 | 9 | 2 | 4.05 | 2.52E-05 | 0.0036 | 1.71 | 0.0438 | 0.4236 |
| *VPS4A* | 16 | 10 | 4 | 4.04 | 2.69E-05 | 0.0038 | 2.75 | 0.0030 | 0.1265 |
| *CENPT* | 16 | 5 | 2 | 4.01 | 3.07E-05 | 0.0041 | 2.15 | 0.0160 | 0.2768 |
| *FEN1* | 11 | 3 | 2 | 3.97 | 3.53E-05 | 0.0046 | 2.96 | 0.0016 | 0.0875 |
| *TP53I13* | 17 | 1 | 1 | 3.95 | 3.93E-05 | 0.0049 | 2.34 | 0.0097 | 0.2174 |
| *PIGU* | 20 | 43 | 5 | 3.95 | 3.96E-05 | 0.0049 | 3.04 | 0.0012 | 0.0787 |
| *NT5DC2* | 3 | 5 | 1 | 3.93 | 4.24E-05 | 0.0051 | 2.57 | 0.0050 | 0.1620 |
| *KCNG1* | 20 | 6 | 2 | 3.92 | 4.44E-05 | 0.0052 | 2.82 | 0.0024 | 0.1128 |
| *SNTB2* | 16 | 24 | 4 | 3.92 | 4.48E-05 | 0.0052 | 2.28 | 0.0114 | 0.2353 |
| *ZNF276* | 16 | 25 | 6 | 3.91 | 4.66E-05 | 0.0053 | 2.10 | 0.0177 | 0.2950 |
| *JADE2* | 5 | 40 | 10 | 3.90 | 4.90E-05 | 0.0055 | 2.33 | 0.0098 | 0.2184 |
| *CIRH1A* | 16 | 23 | 4 | 3.87 | 5.47E-05 | 0.0058 | 2.34 | 0.0096 | 0.2171 |
| *RP11-343C2.12* | 16 | 1 | 1 | 3.85 | 5.93E-05 | 0.0061 | 2.44 | 0.0074 | 0.1983 |
| *MSI1* | 12 | 15 | 3 | 3.83 | 6.39E-05 | 0.0065 | 2.53 | 0.0058 | 0.1731 |
| *LCAT* | 16 | 2 | 1 | 3.78 | 7.78E-05 | 0.0074 | 1.99 | 0.0233 | 0.3338 |
| *PHF7* | 3 | 4 | 2 | 3.78 | 7.98E-05 | 0.0075 | 2.05 | 0.0201 | 0.3109 |
| *ZNF584* | 19 | 15 | 2 | 3.77 | 8.07E-05 | 0.0075 | 2.47 | 0.0068 | 0.1911 |
| *MACROD2* | 20 | 2242 | 244 | 3.77 | 8.31E-05 | 0.0076 | 1.81 | 0.0353 | 0.3905 |
| *RP11-343C2.11* | 16 | 9 | 2 | 3.75 | 8.75E-05 | 0.0078 | 2.96 | 0.0015 | 0.0870 |
| *UBAP2* | 9 | 72 | 8 | 3.71 | 0.0001023 | 0.0087 | 1.68 | 0.0464 | 0.4285 |
| *FANCA* | 16 | 81 | 7 | 3.71 | 0.000104 | 0.0088 | 1.74 | 0.0406 | 0.4094 |
| *NIP7* | 16 | 4 | 1 | 3.70 | 0.0001071 | 0.0089 | 2.34 | 0.0095 | 0.2169 |
| *CSDE1* | 1 | 22 | 3 | 3.67 | 0.0001197 | 0.0096 | 1.86 | 0.0316 | 0.3762 |
| *ALAS1* | 3 | 9 | 1 | 3.67 | 0.0001214 | 0.0096 | 3.19 | 0.0007 | 0.0625 |
| *TRPC4AP* | 20 | 56 | 6 | 3.67 | 0.0001232 | 0.0097 | 2.06 | 0.0197 | 0.3093 |
| *CLCN6* | 1 | 39 | 7 | 3.65 | 0.0001308 | 0.0100 | 1.80 | 0.0361 | 0.3933 |
| *LMBR1L* | 12 | 5 | 1 | 3.62 | 0.0001466 | 0.0107 | 1.73 | 0.0417 | 0.4147 |
| *RP11-894J14.5* | 3 | 71 | 7 | 3.60 | 0.0001606 | 0.0113 | 2.80 | 0.0026 | 0.1159 |
| *NKAPL* | 6 | 4 | 2 | 3.60 | 0.0001608 | 0.0113 | 1.97 | 0.0243 | 0.3370 |
| *SLC4A10* | 2 | 253 | 19 | 3.58 | 0.0001711 | 0.0117 | 1.88 | 0.0298 | 0.3664 |
| *GIT1* | 17 | 7 | 3 | 3.55 | 0.0001915 | 0.0128 | 2.10 | 0.0180 | 0.2971 |
| *ATXN7* | 3 | 54 | 8 | 3.52 | 0.0002167 | 0.0137 | 1.65 | 0.0493 | 0.4368 |
| *ATXN7L1* | 7 | 220 | 58 | 3.50 | 0.0002345 | 0.0144 | 1.98 | 0.0237 | 0.3352 |
| *ACHE* | 7 | 1 | 1 | 3.47 | 0.0002565 | 0.0152 | 1.72 | 0.0430 | 0.4214 |
| *BIN3* | 8 | 43 | 6 | 3.43 | 0.0002996 | 0.0170 | 1.94 | 0.0263 | 0.3477 |
| *RANBP10* | 16 | 10 | 3 | 3.43 | 0.0003065 | 0.0173 | 1.96 | 0.0248 | 0.3399 |
| *ITCH* | 20 | 40 | 2 | 3.38 | 0.0003597 | 0.0195 | 2.94 | 0.0017 | 0.0903 |
| *GSS* | 20 | 11 | 3 | 3.38 | 0.0003634 | 0.0196 | 2.89 | 0.0019 | 0.0985 |
| *FAM189A2* | 9 | 84 | 12 | 3.35 | 0.0004049 | 0.0207 | 2.25 | 0.0121 | 0.2412 |
| *PSMB10* | 16 | 2 | 1 | 3.34 | 0.0004244 | 0.0215 | 1.78 | 0.0378 | 0.4021 |
| *CTC-479C5.12* | 16 | 2 | 1 | 3.31 | 0.0004603 | 0.0227 | 1.77 | 0.0381 | 0.4022 |
| *CATSPER1* | 11 | 8 | 3 | 3.31 | 0.0004671 | 0.0230 | 2.54 | 0.0056 | 0.1714 |
| *KIAA1524* | 3 | 15 | 4 | 3.27 | 0.0005462 | 0.0253 | 2.15 | 0.0158 | 0.2752 |
| *MKRN2* | 3 | 18 | 4 | 3.26 | 0.0005557 | 0.0257 | 2.88 | 0.0020 | 0.0999 |
| *ZNF101* | 19 | 7 | 2 | 3.24 | 0.0005971 | 0.0269 | 1.70 | 0.0442 | 0.4247 |
| *ZNF132* | 19 | 9 | 2 | 3.23 | 0.0006239 | 0.0275 | 1.84 | 0.0332 | 0.3817 |
| *C3orf83* | 3 | 21 | 3 | 3.22 | 0.0006416 | 0.0280 | 3.21 | 0.0007 | 0.0601 |
| *RFT1* | 3 | 25 | 3 | 3.21 | 0.0006662 | 0.0286 | 2.49 | 0.0063 | 0.1830 |
| *XPNPEP1* | 10 | 27 | 4 | 3.21 | 0.0006662 | 0.0286 | 2.18 | 0.0145 | 0.2661 |
| *TP53INP2* | 20 | 4 | 1 | 3.20 | 0.0006758 | 0.0289 | 2.82 | 0.0024 | 0.1123 |
| *AC003043.1* | 17 | 9 | 5 | 3.20 | 0.0006902 | 0.0292 | 2.01 | 0.0221 | 0.3263 |
| *CA10* | 17 | 464 | 51 | 3.19 | 0.0007104 | 0.0298 | 2.63 | 0.0042 | 0.1480 |
| *EDC4* | 16 | 2 | 1 | 3.18 | 0.0007335 | 0.0306 | 1.75 | 0.0399 | 0.4076 |
| *HAS3* | 16 | 4 | 2 | 3.18 | 0.0007429 | 0.0307 | 2.00 | 0.0228 | 0.3315 |
| *HS6ST3* | 13 | 518 | 43 | 3.17 | 0.0007513 | 0.0309 | 1.75 | 0.0404 | 0.4089 |
| *TCF12* | 15 | 305 | 13 | 3.16 | 0.0007946 | 0.0319 | 2.77 | 0.0028 | 0.1226 |
| *SPHKAP* | 2 | 154 | 18 | 3.16 | 0.0008001 | 0.0319 | 1.85 | 0.0325 | 0.3808 |
| *GDAP1* | 8 | 109 | 12 | 3.15 | 0.0008175 | 0.0323 | 2.39 | 0.0085 | 0.2073 |
| *VPS9D1* | 16 | 2 | 1 | 3.15 | 0.000825 | 0.0325 | 1.66 | 0.0488 | 0.4356 |
| *SETD1B* | 12 | 13 | 3 | 3.14 | 0.0008332 | 0.0325 | 1.88 | 0.0302 | 0.3671 |
| *HSD11B2* | 16 | 1 | 1 | 3.14 | 0.0008587 | 0.0332 | 2.41 | 0.0079 | 0.2030 |
| *SLC12A4* | 16 | 8 | 3 | 3.13 | 0.0008604 | 0.0332 | 1.93 | 0.0267 | 0.3505 |
| *MSRA* | 8 | 545 | 43 | 3.13 | 0.0008656 | 0.0332 | 1.90 | 0.0285 | 0.3595 |
| *SUMO2* | 17 | 1 | 1 | 3.13 | 0.0008673 | 0.0332 | 1.71 | 0.0440 | 0.4241 |
| *CCAR2* | 8 | 18 | 2 | 3.11 | 0.0009289 | 0.0346 | 2.27 | 0.0116 | 0.2377 |
| *RPGRIP1L* | 16 | 67 | 8 | 3.11 | 0.0009333 | 0.0347 | 2.74 | 0.0030 | 0.1271 |
| *ATP6V0D1* | 16 | 12 | 3 | 3.10 | 0.0009591 | 0.0354 | 2.26 | 0.0119 | 0.2401 |
| *MFAP3L* | 4 | 38 | 6 | 3.10 | 0.0009763 | 0.0357 | 1.93 | 0.0270 | 0.3514 |
| *TOB1* | 17 | 1 | 1 | 3.05 | 0.001162 | 0.0406 | 1.68 | 0.0460 | 0.4276 |
| *CWF19L1* | 10 | 24 | 4 | 3.02 | 0.0012759 | 0.0431 | 1.82 | 0.0345 | 0.3864 |
| *PRR12* | 19 | 5 | 1 | 3.00 | 0.0013291 | 0.0442 | 1.94 | 0.0260 | 0.3466 |
| *PLXNA4* | 7 | 485 | 80 | 2.99 | 0.0013733 | 0.0453 | 1.77 | 0.0384 | 0.4037 |
| *DNAJC17* | 15 | 13 | 2 | 2.95 | 0.0015989 | 0.0489 | 3.17 | 0.0008 | 0.0638 |
| *SLC35C1* | 11 | 6 | 3 | 2.93 | 0.0016841 | 0.0503 | 2.65 | 0.0040 | 0.1441 |
| *FLJ00418* | 16 | 1 | 1 | 2.93 | 0.001722 | 0.0509 | 1.85 | 0.0320 | 0.3784 |
| *BIRC6* | 2 | 64 | 8 | 2.91 | 0.0017938 | 0.0522 | 2.17 | 0.0150 | 0.2696 |
| *FDPS* | 1 | 3 | 1 | 2.91 | 0.001795 | 0.0522 | 1.89 | 0.0294 | 0.3645 |
| *SFPQ* | 1 | 5 | 1 | 2.91 | 0.0018168 | 0.0525 | 2.05 | 0.0203 | 0.3125 |
| *ASH1L* | 1 | 24 | 2 | 2.91 | 0.0018287 | 0.0527 | 1.68 | 0.0465 | 0.4289 |
| *UBE3C* | 7 | 90 | 9 | 2.90 | 0.0018397 | 0.0528 | 3.42 | 0.0003 | 0.0374 |
| *RSPH3* | 6 | 27 | 4 | 2.90 | 0.0018817 | 0.0536 | 2.29 | 0.0109 | 0.2316 |
| *KCTD13* | 16 | 10 | 2 | 2.90 | 0.0018797 | 0.0536 | 1.92 | 0.0273 | 0.3529 |
| *INO80E* | 16 | 4 | 1 | 2.89 | 0.0019334 | 0.0539 | 3.50 | 0.0002 | 0.0299 |
| *EXTL1* | 1 | 10 | 3 | 2.89 | 0.0019328 | 0.0539 | 1.71 | 0.0436 | 0.4229 |
| *ZFYVE19* | 15 | 10 | 2 | 2.88 | 0.0020123 | 0.0550 | 2.74 | 0.0031 | 0.1279 |
| *GAPDH* | 12 | 4 | 1 | 2.87 | 0.0020267 | 0.0552 | 1.94 | 0.0259 | 0.3466 |
| *WFIKKN2* | 17 | 5 | 1 | 2.86 | 0.0021457 | 0.0577 | 2.13 | 0.0167 | 0.2845 |
| *WDR82* | 3 | 8 | 2 | 2.84 | 0.0022535 | 0.0587 | 2.86 | 0.0021 | 0.1051 |
| *FAM135B* | 8 | 425 | 70 | 2.84 | 0.002229 | 0.0587 | 2.41 | 0.0079 | 0.2030 |
| *RORB* | 9 | 161 | 22 | 2.84 | 0.0022539 | 0.0587 | 2.18 | 0.0146 | 0.2669 |
| *NCAN* | 19 | 15 | 4 | 2.84 | 0.0022475 | 0.0587 | 2.10 | 0.0179 | 0.2965 |
| *ESCO2* | 8 | 45 | 6 | 2.84 | 0.002251 | 0.0587 | 1.78 | 0.0377 | 0.4021 |
| *NAPA* | 19 | 16 | 3 | 2.84 | 0.0022747 | 0.0589 | 1.65 | 0.0496 | 0.4374 |
| *GOSR1* | 17 | 13 | 3 | 2.83 | 0.0023315 | 0.0598 | 1.73 | 0.0420 | 0.4157 |
| *UQCR10* | 22 | 2 | 1 | 2.82 | 0.0023897 | 0.0607 | 4.45 | 4.3E-06 | 0.0022 |
| *CCDC25* | 8 | 60 | 6 | 2.81 | 0.0024588 | 0.0621 | 1.96 | 0.0248 | 0.3399 |
| *TUBA1A* | 12 | 1 | 1 | 2.79 | 0.002605 | 0.0640 | 1.67 | 0.0470 | 0.4313 |
| *ZNF7* | 8 | 21 | 3 | 2.79 | 0.0026189 | 0.0641 | 2.13 | 0.0166 | 0.2838 |
| *NDST3* | 4 | 161 | 15 | 2.78 | 0.0027441 | 0.0662 | 3.14 | 0.0009 | 0.0679 |
| *COPG1* | 3 | 10 | 2 | 2.77 | 0.0027863 | 0.0665 | 2.36 | 0.0090 | 0.2119 |
| *EDEM2* | 20 | 77 | 12 | 2.77 | 0.0028281 | 0.0670 | 3.05 | 0.0012 | 0.0787 |
| *MYH7B* | 20 | 15 | 3 | 2.77 | 0.0028268 | 0.0670 | 2.66 | 0.0039 | 0.1419 |
| *RLTPR* | 16 | 2 | 1 | 2.74 | 0.0031055 | 0.0706 | 2.21 | 0.0134 | 0.2556 |
| *HNF1A* | 12 | 28 | 4 | 2.73 | 0.0031712 | 0.0717 | 3.01 | 0.0013 | 0.0815 |
| *MAPK10* | 4 | 331 | 31 | 2.72 | 0.003237 | 0.0727 | 3.24 | 0.0006 | 0.0555 |
| *ZMIZ1* | 10 | 278 | 55 | 2.72 | 0.003267 | 0.0729 | 6.32 | 1.3E-10 | 2.3E-07 |
| *ZDHHC1* | 16 | 6 | 2 | 2.72 | 0.0032607 | 0.0729 | 2.16 | 0.0155 | 0.2718 |
| *MAP1LC3B* | 16 | 10 | 4 | 2.71 | 0.0033623 | 0.0739 | 1.69 | 0.0455 | 0.4264 |
| *TBRG4* | 7 | 18 | 5 | 2.71 | 0.0033822 | 0.0743 | 2.40 | 0.0083 | 0.2055 |
| *TSEN2* | 3 | 41 | 5 | 2.69 | 0.0035593 | 0.0769 | 3.34 | 0.0004 | 0.0456 |
| *LRRC36* | 16 | 19 | 3 | 2.69 | 0.0035941 | 0.0771 | 2.11 | 0.0175 | 0.2937 |
| *ERBB2* | 17 | 8 | 3 | 2.68 | 0.0036337 | 0.0778 | 2.29 | 0.0110 | 0.2328 |
| *SEMA3G* | 3 | 6 | 3 | 2.67 | 0.0037535 | 0.0794 | 2.96 | 0.0015 | 0.0870 |
| *DDX19A* | 16 | 5 | 1 | 2.67 | 0.0038442 | 0.0810 | 1.65 | 0.0495 | 0.4370 |
| *PGAP3* | 17 | 24 | 1 | 2.66 | 0.0039287 | 0.0822 | 2.35 | 0.0095 | 0.2169 |
| *NRAS* | 1 | 3 | 1 | 2.66 | 0.0039405 | 0.0824 | 1.78 | 0.0373 | 0.4002 |
| *FNDC5* | 1 | 5 | 2 | 2.65 | 0.0039677 | 0.0826 | 1.67 | 0.0479 | 0.4328 |
| *CREBZF* | 11 | 13 | 4 | 2.65 | 0.0040405 | 0.0832 | 2.20 | 0.0139 | 0.2612 |
| *RGS17* | 6 | 130 | 15 | 2.65 | 0.0040719 | 0.0833 | 2.60 | 0.0046 | 0.1528 |
| *NOSIP* | 19 | 10 | 2 | 2.63 | 0.004227 | 0.0851 | 1.78 | 0.0378 | 0.4021 |
| *OR10A3* | 11 | 1 | 1 | 2.63 | 0.004245 | 0.0854 | 1.67 | 0.0470 | 0.4313 |
| *WBP1L* | 10 | 63 | 10 | 2.63 | 0.0042512 | 0.0854 | 2.52 | 0.0059 | 0.1741 |
| *ZNF839* | 14 | 12 | 4 | 2.62 | 0.0044403 | 0.0875 | 3.36 | 0.0004 | 0.0430 |
| *C8orf46* | 8 | 54 | 12 | 2.60 | 0.0046086 | 0.0895 | 2.29 | 0.0110 | 0.2322 |
| *RASGRP1* | 15 | 26 | 8 | 2.60 | 0.0046943 | 0.0900 | 2.29 | 0.0112 | 0.2343 |
| *RGS12* | 4 | 98 | 17 | 2.60 | 0.0046853 | 0.0900 | 1.77 | 0.0381 | 0.4022 |
| *ZNF250* | 8 | 13 | 2 | 2.59 | 0.0047372 | 0.0905 | 1.96 | 0.0248 | 0.3399 |
| *COMMD5* | 8 | 18 | 2 | 2.59 | 0.0047868 | 0.0913 | 2.10 | 0.0177 | 0.2950 |
| *FAM83E* | 19 | 5 | 2 | 2.58 | 0.0048934 | 0.0927 | 1.67 | 0.0479 | 0.4328 |
| *DVL2* | 17 | 5 | 2 | 2.57 | 0.0050409 | 0.0945 | 3.32 | 0.0005 | 0.0476 |
| *HAGH* | 16 | 26 | 4 | 2.57 | 0.0051014 | 0.0952 | 2.70 | 0.0035 | 0.1343 |
| *IL34* | 16 | 29 | 5 | 2.57 | 0.0051166 | 0.0953 | 1.93 | 0.0266 | 0.3491 |
| *RAF1* | 3 | 50 | 7 | 2.55 | 0.0053209 | 0.0980 | 2.13 | 0.0166 | 0.2840 |
| *TRABD2A* | 2 | 61 | 8 | 2.55 | 0.0053509 | 0.0981 | 1.71 | 0.0434 | 0.4223 |
| *BMPR1B* | 4 | 382 | 35 | 2.55 | 0.0054096 | 0.0987 | 1.76 | 0.0389 | 0.4043 |
| *AC079210.1* | 17 | 5 | 2 | 2.54 | 0.0055068 | 0.0995 | 1.81 | 0.0353 | 0.3903 |
| *FCHO2* | 5 | 41 | 5 | 2.53 | 0.005632 | 0.1008 | 2.32 | 0.0101 | 0.2229 |
| *AMN* | 14 | 4 | 2 | 2.53 | 0.0056244 | 0.1008 | 1.93 | 0.0271 | 0.3514 |
| *CACYBP* | 1 | 6 | 2 | 2.53 | 0.0056292 | 0.1008 | 1.71 | 0.0433 | 0.4223 |
| *AADAT* | 4 | 13 | 4 | 2.53 | 0.0057109 | 0.1018 | 1.87 | 0.0309 | 0.3714 |
| *TUBA1B* | 12 | 2 | 1 | 2.53 | 0.0057658 | 0.1021 | 1.67 | 0.0473 | 0.4327 |
| *SIGLECL1* | 19 | 22 | 4 | 2.52 | 0.0058481 | 0.1030 | 1.99 | 0.0234 | 0.3339 |
| *MACF1* | 1 | 154 | 15 | 2.52 | 0.0059217 | 0.1040 | 4.26 | 1.0E-05 | 0.0036 |
| *MYO18A* | 17 | 48 | 6 | 2.51 | 0.0061084 | 0.1056 | 2.41 | 0.0080 | 0.2035 |
| *CASC5* | 15 | 38 | 4 | 2.50 | 0.0061578 | 0.1062 | 2.75 | 0.0030 | 0.1271 |
| *CHCHD3* | 7 | 162 | 11 | 2.50 | 0.0062459 | 0.1074 | 2.63 | 0.0043 | 0.1485 |
| *FAM102A* | 9 | 19 | 2 | 2.49 | 0.0064122 | 0.1085 | 2.62 | 0.0044 | 0.1492 |
| *C7orf55-LUC7L2* | 7 | 53 | 2 | 2.49 | 0.0064379 | 0.1085 | 1.68 | 0.0464 | 0.4285 |
| *LUC7L2* | 7 | 53 | 2 | 2.49 | 0.0064379 | 0.1085 | 1.68 | 0.0464 | 0.4285 |
| *YIPF4* | 2 | 12 | 4 | 2.49 | 0.0064767 | 0.1088 | 1.94 | 0.0263 | 0.3477 |
| *PRRG2* | 19 | 4 | 1 | 2.48 | 0.0065291 | 0.1094 | 1.98 | 0.0239 | 0.3352 |
| *ARID2* | 12 | 36 | 4 | 2.47 | 0.0067377 | 0.1120 | 2.60 | 0.0047 | 0.1546 |
| *ZSCAN31* | 6 | 33 | 6 | 2.47 | 0.0068508 | 0.1128 | 1.68 | 0.0466 | 0.4289 |
| *ANKRD36* | 2 | 7 | 2 | 2.46 | 0.0068676 | 0.1128 | 1.66 | 0.0487 | 0.4356 |
| *SYT7* | 11 | 9 | 3 | 2.45 | 0.007046 | 0.1147 | 1.78 | 0.0375 | 0.4012 |
| *CHST1* | 11 | 12 | 3 | 2.45 | 0.0071497 | 0.1158 | 1.80 | 0.0362 | 0.3933 |
| *GIPR* | 19 | 3 | 2 | 2.44 | 0.0074134 | 0.1185 | 3.53 | 0.0002 | 0.0280 |
| *ETFB* | 19 | 13 | 3 | 2.44 | 0.0074248 | 0.1185 | 1.71 | 0.0434 | 0.4223 |
| *GMIP* | 19 | 4 | 2 | 2.43 | 0.0074924 | 0.1189 | 2.01 | 0.0220 | 0.3261 |
| *QKI* | 6 | 126 | 10 | 2.43 | 0.0075715 | 0.1196 | 2.08 | 0.0186 | 0.3016 |
| *ISLR* | 15 | 6 | 1 | 2.41 | 0.0080538 | 0.1247 | 1.91 | 0.0281 | 0.3582 |
| *PROX1* | 1 | 20 | 4 | 2.39 | 0.0084715 | 0.1280 | 4.41 | 5.2E-06 | 0.0022 |
| *PTPRE* | 10 | 165 | 45 | 2.37 | 0.0088656 | 0.1308 | 1.80 | 0.0360 | 0.3929 |
| *OASL* | 12 | 13 | 5 | 2.36 | 0.0091182 | 0.1332 | 3.26 | 0.0006 | 0.0537 |
| *TCAP* | 17 | 2 | 1 | 2.36 | 0.009155 | 0.1334 | 1.88 | 0.0298 | 0.3664 |
| *HMCES* | 3 | 6 | 2 | 2.36 | 0.0092351 | 0.1341 | 2.78 | 0.0027 | 0.1189 |
| *LRP5* | 11 | 64 | 10 | 2.35 | 0.0094713 | 0.1358 | 1.77 | 0.0380 | 0.4021 |
| *HS3ST6* | 16 | 4 | 1 | 2.35 | 0.0094959 | 0.1359 | 1.89 | 0.0294 | 0.3645 |
| *C7orf55* | 7 | 5 | 1 | 2.34 | 0.0095881 | 0.1363 | 1.71 | 0.0432 | 0.4223 |
| *MKL1* | 22 | 72 | 7 | 2.34 | 0.0097609 | 0.1371 | 2.17 | 0.0152 | 0.2699 |
| *NUS1* | 6 | 15 | 3 | 2.33 | 0.0098564 | 0.1380 | 3.53 | 0.0002 | 0.0280 |
| *H1FX* | 3 | 2 | 1 | 2.32 | 0.010076 | 0.1397 | 2.45 | 0.0072 | 0.1960 |
| *RPS6KA5* | 14 | 96 | 9 | 2.29 | 0.01101 | 0.1487 | 2.07 | 0.0191 | 0.3047 |
| *FEZF1* | 7 | 1 | 1 | 2.28 | 0.01116 | 0.1495 | 1.91 | 0.0280 | 0.3579 |
| *C15orf62* | 15 | 1 | 1 | 2.28 | 0.01141 | 0.1510 | 2.85 | 0.0022 | 0.1070 |
| *SLC22A7* | 6 | 4 | 2 | 2.27 | 0.01148 | 0.1513 | 3.21 | 0.0007 | 0.0604 |
| *C1orf185* | 1 | 7 | 2 | 2.26 | 0.011859 | 0.1540 | 1.90 | 0.0286 | 0.3596 |
| *MRAS* | 3 | 40 | 4 | 2.26 | 0.01194 | 0.1546 | 2.38 | 0.0086 | 0.2080 |
| *SETD2* | 3 | 38 | 3 | 2.26 | 0.012021 | 0.1554 | 2.42 | 0.0077 | 0.1995 |
| *SETBP1* | 18 | 241 | 48 | 2.26 | 0.012042 | 0.1554 | 2.40 | 0.0082 | 0.2055 |
| *COX7A1* | 19 | 1 | 1 | 2.26 | 0.01204 | 0.1554 | 1.85 | 0.0320 | 0.3784 |
| *EZH2* | 7 | 40 | 6 | 2.26 | 0.012063 | 0.1554 | 1.91 | 0.0282 | 0.3582 |
| *NBEAL2* | 3 | 9 | 2 | 2.25 | 0.012308 | 0.1574 | 2.25 | 0.0121 | 0.2412 |
| *SCAF11* | 12 | 26 | 4 | 2.25 | 0.01235 | 0.1574 | 2.49 | 0.0065 | 0.1853 |
| *ADHFE1* | 8 | 45 | 7 | 2.24 | 0.012643 | 0.1590 | 2.52 | 0.0058 | 0.1741 |
| *RTN4* | 2 | 126 | 16 | 2.24 | 0.012692 | 0.1592 | 3.62 | 0.0001 | 0.0240 |
| *PSMD6* | 3 | 12 | 3 | 2.23 | 0.012739 | 0.1592 | 1.98 | 0.0236 | 0.3352 |
| *SPG7* | 16 | 37 | 4 | 2.23 | 0.012829 | 0.1594 | 2.66 | 0.0040 | 0.1420 |
| *CAMKK2* | 12 | 34 | 9 | 2.23 | 0.012925 | 0.1600 | 1.88 | 0.0300 | 0.3667 |
| *SMEK1* | 14 | 42 | 4 | 2.23 | 0.012988 | 0.1601 | 3.24 | 0.0006 | 0.0555 |
| *MST1R* | 3 | 6 | 2 | 2.23 | 0.012971 | 0.1601 | 2.17 | 0.0149 | 0.2696 |
| *PNMAL2* | 19 | 3 | 2 | 2.23 | 0.012968 | 0.1601 | 1.81 | 0.0350 | 0.3886 |
| *CLVS2* | 6 | 37 | 5 | 2.22 | 0.013247 | 0.1616 | 1.83 | 0.0336 | 0.3824 |
| *RAP1GAP* | 1 | 57 | 18 | 2.22 | 0.013343 | 0.1624 | 1.65 | 0.0498 | 0.4380 |
| *ADAM32* | 8 | 59 | 6 | 2.21 | 0.013419 | 0.1626 | 2.61 | 0.0046 | 0.1527 |
| *ASIC4* | 2 | 13 | 4 | 2.21 | 0.013636 | 0.1644 | 2.14 | 0.0162 | 0.2792 |
| *SMARCAD1* | 4 | 50 | 5 | 2.20 | 0.013984 | 0.1676 | 3.99 | 3.3E-05 | 0.0082 |
| *ZNF236* | 18 | 101 | 12 | 2.20 | 0.013988 | 0.1676 | 2.23 | 0.0128 | 0.2507 |
| *TOP1* | 20 | 32 | 6 | 2.20 | 0.014071 | 0.1682 | 1.99 | 0.0234 | 0.3339 |
| *IFFO1* | 12 | 3 | 1 | 2.19 | 0.014333 | 0.1690 | 2.56 | 0.0052 | 0.1641 |
| *PMFBP1* | 16 | 50 | 7 | 2.19 | 0.014378 | 0.1691 | 1.76 | 0.0394 | 0.4059 |
| *POP5* | 12 | 5 | 2 | 2.18 | 0.014804 | 0.1718 | 3.01 | 0.0013 | 0.0818 |
| *DPM2* | 9 | 3 | 1 | 2.18 | 0.014773 | 0.1718 | 2.39 | 0.0083 | 0.2061 |
| *BRAF* | 7 | 66 | 5 | 2.17 | 0.014824 | 0.1719 | 2.34 | 0.0096 | 0.2171 |
| *DYNLL1* | 12 | 11 | 3 | 2.17 | 0.015047 | 0.1735 | 2.54 | 0.0055 | 0.1705 |
| *RICTOR* | 5 | 76 | 6 | 2.16 | 0.015236 | 0.1752 | 1.80 | 0.0360 | 0.3929 |
| *MGAT4A* | 2 | 59 | 8 | 2.16 | 0.015439 | 0.1762 | 2.53 | 0.0057 | 0.1722 |
| *PTRF* | 17 | 11 | 4 | 2.15 | 0.0156 | 0.1772 | 2.39 | 0.0084 | 0.2064 |
| *ACADVL* | 17 | 3 | 1 | 2.15 | 0.015659 | 0.1776 | 2.58 | 0.0050 | 0.1612 |
| *GFI1B* | 9 | 33 | 13 | 2.15 | 0.015684 | 0.1776 | 1.68 | 0.0460 | 0.4276 |
| *NECAP1* | 12 | 8 | 3 | 2.14 | 0.016184 | 0.1812 | 2.38 | 0.0087 | 0.2088 |
| *ZNF318* | 6 | 23 | 4 | 2.14 | 0.016277 | 0.1819 | 3.90 | 4.8E-05 | 0.0105 |
| *CTTNBP2* | 7 | 91 | 16 | 2.13 | 0.016662 | 0.1836 | 2.32 | 0.0101 | 0.2230 |
| *ZNF34* | 8 | 11 | 2 | 2.13 | 0.016733 | 0.1837 | 2.67 | 0.0037 | 0.1387 |
| *ELP6* | 3 | 6 | 2 | 2.12 | 0.016983 | 0.1854 | 1.84 | 0.0328 | 0.3808 |
| *OCIAD2* | 4 | 3 | 1 | 2.12 | 0.017153 | 0.1863 | 1.87 | 0.0307 | 0.3695 |
| *FADS3* | 11 | 8 | 3 | 2.11 | 0.017318 | 0.1875 | 2.63 | 0.0043 | 0.1485 |
| *PKNOX1* | 21 | 62 | 9 | 2.11 | 0.017568 | 0.1890 | 2.10 | 0.0177 | 0.2950 |
| *SLC39A1* | 1 | 3 | 2 | 2.11 | 0.0176 | 0.1892 | 2.00 | 0.0226 | 0.3308 |
| *TMEM126B* | 11 | 5 | 2 | 2.09 | 0.018101 | 0.1919 | 2.37 | 0.0088 | 0.2108 |
| *SLCO4C1* | 5 | 48 | 7 | 2.09 | 0.018137 | 0.1921 | 4.22 | 1.2E-05 | 0.0040 |
| *KLHL18* | 3 | 13 | 2 | 2.09 | 0.018195 | 0.1921 | 2.07 | 0.0193 | 0.3060 |
| *TPP2* | 13 | 85 | 6 | 2.09 | 0.018407 | 0.1937 | 1.96 | 0.0252 | 0.3422 |
| *CCND2* | 12 | 29 | 9 | 2.08 | 0.018708 | 0.1956 | 2.73 | 0.0031 | 0.1284 |
| *XRCC3* | 14 | 11 | 3 | 2.08 | 0.018784 | 0.1956 | 1.90 | 0.0285 | 0.3595 |
| *HPGDS* | 4 | 20 | 3 | 2.08 | 0.018817 | 0.1958 | 3.05 | 0.0011 | 0.0776 |
| *FN1* | 2 | 82 | 12 | 2.08 | 0.018878 | 0.1959 | 1.87 | 0.0308 | 0.3707 |
| *PPIL2* | 22 | 38 | 5 | 2.07 | 0.019154 | 0.1976 | 2.48 | 0.0066 | 0.1893 |
| *KIF9* | 3 | 16 | 2 | 2.07 | 0.019371 | 0.1988 | 2.16 | 0.0154 | 0.2713 |
| *SCAP* | 3 | 10 | 1 | 2.06 | 0.019746 | 0.2003 | 2.07 | 0.0190 | 0.3041 |
| *CCT2* | 12 | 21 | 5 | 2.06 | 0.019711 | 0.2003 | 1.72 | 0.0425 | 0.4193 |
| *ZSCAN23* | 6 | 11 | 3 | 2.06 | 0.019827 | 0.2007 | 2.32 | 0.0102 | 0.2232 |
| *KY* | 3 | 40 | 3 | 2.05 | 0.020063 | 0.2020 | 2.20 | 0.0140 | 0.2614 |
| *ITGAL* | 16 | 24 | 9 | 2.04 | 0.020839 | 0.2068 | 2.19 | 0.0143 | 0.2636 |
| *VSTM5* | 11 | 22 | 5 | 2.04 | 0.020803 | 0.2068 | 1.80 | 0.0358 | 0.3925 |
| *ASCC2* | 22 | 38 | 7 | 2.03 | 0.020935 | 0.2068 | 3.28 | 0.0005 | 0.0513 |
| *NSUN3* | 3 | 15 | 4 | 2.03 | 0.02095 | 0.2068 | 2.46 | 0.0070 | 0.1925 |
| *PROS1* | 3 | 19 | 5 | 2.03 | 0.021128 | 0.2077 | 1.97 | 0.0241 | 0.3360 |
| *LMBR1* | 7 | 158 | 19 | 2.02 | 0.021551 | 0.2102 | 1.85 | 0.0325 | 0.3808 |
| *ATXN2L* | 16 | 5 | 1 | 2.02 | 0.021643 | 0.2106 | 1.89 | 0.0296 | 0.3656 |
| *ADAMTS6* | 5 | 225 | 26 | 2.02 | 0.021905 | 0.2124 | 2.30 | 0.0108 | 0.2306 |
| *RILPL2* | 12 | 3 | 2 | 2.01 | 0.022023 | 0.2131 | 1.93 | 0.0267 | 0.3497 |
| *RAB7L1* | 1 | 12 | 3 | 2.00 | 0.022623 | 0.2160 | 2.82 | 0.0024 | 0.1128 |
| *SSR1* | 6 | 46 | 9 | 2.00 | 0.022657 | 0.2160 | 3.31 | 0.0005 | 0.0486 |
| *MSH4* | 1 | 90 | 12 | 2.00 | 0.022928 | 0.2175 | 2.26 | 0.0120 | 0.2402 |
| *ALDOA* | 16 | 3 | 2 | 1.98 | 0.023737 | 0.2205 | 2.31 | 0.0105 | 0.2281 |
| *ACADM* | 1 | 40 | 5 | 1.98 | 0.023843 | 0.2208 | 2.15 | 0.0156 | 0.2726 |
| *CKLF* | 16 | 9 | 3 | 1.98 | 0.024044 | 0.2219 | 1.66 | 0.0485 | 0.4349 |
| *OMP* | 11 | 1 | 1 | 1.98 | 0.02413 | 0.2224 | 2.00 | 0.0230 | 0.3320 |
| *TIAF1* | 17 | 10 | 2 | 1.97 | 0.024558 | 0.2250 | 1.94 | 0.0264 | 0.3477 |
| *STARD3* | 17 | 6 | 2 | 1.97 | 0.02465 | 0.2254 | 1.98 | 0.0238 | 0.3352 |
| *METTL10* | 10 | 15 | 3 | 1.96 | 0.025127 | 0.2274 | 2.20 | 0.0139 | 0.2612 |
| *TSPAN15* | 10 | 63 | 6 | 1.96 | 0.025258 | 0.2282 | 1.73 | 0.0415 | 0.4144 |
| *SPRED1* | 15 | 130 | 5 | 1.94 | 0.026048 | 0.2323 | 2.02 | 0.0217 | 0.3238 |
| *MRPS30* | 5 | 7 | 1 | 1.93 | 0.026657 | 0.2349 | 3.02 | 0.0013 | 0.0810 |
| *NF2* | 22 | 54 | 7 | 1.93 | 0.026658 | 0.2349 | 1.82 | 0.0343 | 0.3853 |
| *ZNF799* | 19 | 2 | 1 | 1.93 | 0.027024 | 0.2364 | 2.22 | 0.0132 | 0.2535 |
| *ZHX3* | 20 | 70 | 9 | 1.92 | 0.027144 | 0.2365 | 2.47 | 0.0068 | 0.1910 |
| *BCAS1* | 20 | 244 | 33 | 1.92 | 0.027339 | 0.2377 | 2.34 | 0.0096 | 0.2171 |
| *EHD4* | 15 | 62 | 13 | 1.92 | 0.027357 | 0.2377 | 1.98 | 0.0238 | 0.3352 |
| *ZNF544* | 19 | 26 | 5 | 1.92 | 0.027696 | 0.2396 | 1.71 | 0.0436 | 0.4229 |
| *NRXN3* | 14 | 1344 | 130 | 1.91 | 0.028022 | 0.2414 | 4.43 | 4.8E-06 | 0.0022 |
| *ZDHHC8* | 22 | 8 | 4 | 1.90 | 0.028587 | 0.2444 | 2.29 | 0.0110 | 0.2322 |
| *MTUS1* | 8 | 240 | 35 | 1.90 | 0.028772 | 0.2454 | 1.84 | 0.0330 | 0.3810 |
| *CCDC12* | 3 | 14 | 3 | 1.90 | 0.028876 | 0.2457 | 2.72 | 0.0032 | 0.1299 |
| *DLG4* | 17 | 11 | 2 | 1.90 | 0.028963 | 0.2460 | 2.91 | 0.0018 | 0.0952 |
| *BLMH* | 17 | 20 | 5 | 1.89 | 0.029334 | 0.2473 | 2.44 | 0.0073 | 0.1966 |
| *NOSTRIN* | 2 | 91 | 13 | 1.89 | 0.029395 | 0.2473 | 1.67 | 0.0479 | 0.4328 |
| *SPC25* | 2 | 76 | 11 | 1.89 | 0.029582 | 0.2480 | 2.81 | 0.0025 | 0.1140 |
| *MVP* | 16 | 9 | 1 | 1.88 | 0.029732 | 0.2488 | 2.52 | 0.0059 | 0.1746 |
| *PEAK1* | 15 | 109 | 10 | 1.88 | 0.029806 | 0.2490 | 4.35 | 6.7E-06 | 0.0027 |
| *CRHR2* | 7 | 33 | 8 | 1.88 | 0.02982 | 0.2490 | 3.19 | 0.0007 | 0.0625 |
| *PTPN23* | 3 | 6 | 1 | 1.88 | 0.029793 | 0.2490 | 2.16 | 0.0154 | 0.2713 |
| *TCF25* | 16 | 19 | 2 | 1.88 | 0.029878 | 0.2493 | 1.75 | 0.0397 | 0.4066 |
| *MUS81* | 11 | 5 | 1 | 1.88 | 0.029942 | 0.2497 | 3.00 | 0.0014 | 0.0836 |
| *ELSPBP1* | 19 | 35 | 7 | 1.88 | 0.030049 | 0.2502 | 2.17 | 0.0151 | 0.2696 |
| *AC037459.4* | 8 | 8 | 2 | 1.88 | 0.030295 | 0.2517 | 1.86 | 0.0314 | 0.3751 |
| *RNF10* | 12 | 22 | 6 | 1.87 | 0.030521 | 0.2528 | 1.87 | 0.0307 | 0.3695 |
| *SPATA20* | 17 | 5 | 2 | 1.87 | 0.030729 | 0.2539 | 3.97 | 3.5E-05 | 0.0084 |
| *MAP6* | 11 | 58 | 8 | 1.87 | 0.031042 | 0.2557 | 1.81 | 0.0354 | 0.3905 |
| *CTD-2616J11.11* | 19 | 6 | 2 | 1.86 | 0.031374 | 0.2569 | 2.05 | 0.0203 | 0.3125 |
| *CRY2* | 11 | 19 | 5 | 1.86 | 0.031714 | 0.2576 | 2.70 | 0.0035 | 0.1343 |
| *SLA2* | 20 | 4 | 2 | 1.86 | 0.031746 | 0.2576 | 2.05 | 0.0203 | 0.3125 |
| *MSANTD1* | 4 | 15 | 3 | 1.85 | 0.032099 | 0.2598 | 1.69 | 0.0457 | 0.4270 |
| *MTMR3* | 22 | 103 | 14 | 1.85 | 0.03228 | 0.2607 | 3.81 | 0.0001 | 0.0138 |
| *PKD2L1* | 10 | 34 | 8 | 1.85 | 0.032382 | 0.2610 | 2.62 | 0.0044 | 0.1504 |
| *EPPIN-WFDC6* | 20 | 5 | 3 | 1.84 | 0.032635 | 0.2617 | 1.76 | 0.0392 | 0.4051 |
| *THADA* | 2 | 318 | 20 | 1.84 | 0.032763 | 0.2619 | 6.65 | 1.4E-11 | 2.8E-08 |
| *GARNL3* | 9 | 95 | 14 | 1.84 | 0.032722 | 0.2619 | 2.31 | 0.0104 | 0.2265 |
| *C8orf58* | 8 | 1 | 1 | 1.84 | 0.03294 | 0.2625 | 1.81 | 0.0350 | 0.3886 |
| *MITF* | 3 | 105 | 7 | 1.84 | 0.033254 | 0.2639 | 2.12 | 0.0168 | 0.2851 |
| *PARD3* | 10 | 565 | 40 | 1.83 | 0.033618 | 0.2654 | 1.71 | 0.0434 | 0.4223 |
| *DNAJC11* | 1 | 30 | 5 | 1.83 | 0.033923 | 0.2661 | 2.15 | 0.0157 | 0.2732 |
| *MAP2K5* | 15 | 191 | 17 | 1.83 | 0.033925 | 0.2661 | 1.82 | 0.0345 | 0.3864 |
| *GPSM1* | 9 | 5 | 1 | 1.82 | 0.03406 | 0.2666 | 3.02 | 0.0013 | 0.0810 |
| *AL021546.6* | 12 | 7 | 3 | 1.82 | 0.034173 | 0.2666 | 2.26 | 0.0119 | 0.2401 |
| *MAN1C1* | 1 | 54 | 9 | 1.82 | 0.034172 | 0.2666 | 1.97 | 0.0244 | 0.3370 |
| *TMC5* | 16 | 69 | 14 | 1.82 | 0.034488 | 0.2675 | 2.03 | 0.0214 | 0.3208 |
| *ADAMTSL3* | 15 | 275 | 24 | 1.82 | 0.034554 | 0.2678 | 2.78 | 0.0027 | 0.1193 |
| *CFL1* | 11 | 10 | 2 | 1.82 | 0.034749 | 0.2685 | 3.08 | 0.0010 | 0.0745 |
| *DOC2A* | 16 | 9 | 1 | 1.81 | 0.035168 | 0.2697 | 3.63 | 0.0001 | 0.0238 |
| *OGDH* | 7 | 47 | 8 | 1.81 | 0.035178 | 0.2697 | 2.74 | 0.0031 | 0.1282 |
| *ECT2* | 3 | 28 | 4 | 1.81 | 0.035184 | 0.2697 | 2.43 | 0.0076 | 0.1995 |
| *BCL6* | 3 | 16 | 4 | 1.81 | 0.035092 | 0.2697 | 2.08 | 0.0190 | 0.3039 |
| *HOXC4* | 12 | 10 | 2 | 1.81 | 0.03553 | 0.2706 | 2.95 | 0.0016 | 0.0879 |
| *PITPNM2* | 12 | 48 | 6 | 1.80 | 0.035586 | 0.2707 | 3.73 | 0.0001 | 0.0176 |
| *PTPRN2* | 7 | 671 | 125 | 1.80 | 0.035902 | 0.2718 | 3.37 | 0.0004 | 0.0426 |
| *C12orf65* | 12 | 13 | 2 | 1.80 | 0.036078 | 0.2725 | 3.18 | 0.0007 | 0.0625 |
| *PRRT2* | 16 | 2 | 1 | 1.80 | 0.036219 | 0.2729 | 2.14 | 0.0161 | 0.2784 |
| *FABP4* | 8 | 9 | 2 | 1.78 | 0.037235 | 0.2768 | 4.14 | 1.8E-05 | 0.0052 |
| *TOPAZ1* | 3 | 55 | 6 | 1.78 | 0.037172 | 0.2768 | 1.92 | 0.0273 | 0.3528 |
| *GLI2* | 2 | 189 | 37 | 1.78 | 0.037473 | 0.2779 | 2.29 | 0.0109 | 0.2315 |
| *PDLIM2* | 8 | 11 | 3 | 1.78 | 0.037733 | 0.2784 | 2.09 | 0.0184 | 0.3003 |
| *ZSCAN12* | 6 | 31 | 5 | 1.77 | 0.038159 | 0.2807 | 2.70 | 0.0035 | 0.1343 |
| *FAM46A* | 6 | 211 | 28 | 1.77 | 0.038313 | 0.2811 | 2.41 | 0.0081 | 0.2042 |
| *SLC16A10* | 6 | 66 | 8 | 1.76 | 0.038978 | 0.2829 | 1.76 | 0.0394 | 0.4059 |
| *FAM133B* | 7 | 15 | 3 | 1.76 | 0.039403 | 0.2851 | 2.16 | 0.0152 | 0.2701 |
| *CYBRD1* | 2 | 22 | 6 | 1.75 | 0.039786 | 0.2870 | 2.22 | 0.0134 | 0.2554 |
| *GALNT6* | 12 | 37 | 12 | 1.74 | 0.041017 | 0.2926 | 2.07 | 0.0192 | 0.3047 |
| *TUBA1C* | 12 | 20 | 5 | 1.74 | 0.041014 | 0.2926 | 1.72 | 0.0425 | 0.4193 |
| *AQP6* | 12 | 4 | 2 | 1.74 | 0.041159 | 0.2930 | 1.75 | 0.0403 | 0.4083 |
| *PLCG1* | 20 | 16 | 4 | 1.74 | 0.04135 | 0.2939 | 2.17 | 0.0151 | 0.2696 |
| *TMEM81* | 1 | 4 | 2 | 1.73 | 0.04156 | 0.2949 | 2.09 | 0.0181 | 0.2981 |
| *ARL15* | 5 | 437 | 57 | 1.73 | 0.041855 | 0.2956 | 4.08 | 2.2E-05 | 0.0061 |
| *CTC-236F12.4* | 5 | 26 | 8 | 1.71 | 0.043466 | 0.3018 | 2.13 | 0.0164 | 0.2817 |
| *HORMAD2* | 22 | 59 | 9 | 1.71 | 0.04387 | 0.3030 | 3.38 | 0.0004 | 0.0422 |
| *COQ5* | 12 | 10 | 4 | 1.71 | 0.043951 | 0.3032 | 2.40 | 0.0083 | 0.2055 |
| *FRAT1* | 10 | 2 | 1 | 1.70 | 0.044536 | 0.3047 | 1.77 | 0.0386 | 0.4038 |
| *PAGR1* | 16 | 4 | 1 | 1.70 | 0.04463 | 0.3049 | 2.66 | 0.0039 | 0.1408 |
| *SCUBE2* | 11 | 86 | 19 | 1.70 | 0.044708 | 0.3050 | 1.84 | 0.0328 | 0.3808 |
| *DRD2* | 11 | 69 | 10 | 1.70 | 0.044779 | 0.3053 | 2.26 | 0.0120 | 0.2406 |
| *RANBP1* | 22 | 4 | 2 | 1.69 | 0.045461 | 0.3073 | 2.09 | 0.0184 | 0.3003 |
| *RCBTB1* | 13 | 81 | 8 | 1.69 | 0.0457 | 0.3082 | 1.83 | 0.0339 | 0.3840 |
| *MXI1* | 10 | 42 | 7 | 1.68 | 0.04619 | 0.3100 | 2.48 | 0.0065 | 0.1853 |
| *ESF1* | 20 | 54 | 8 | 1.68 | 0.046164 | 0.3100 | 1.79 | 0.0369 | 0.3983 |
| *SNX19* | 11 | 81 | 7 | 1.68 | 0.046276 | 0.3104 | 2.93 | 0.0017 | 0.0907 |
| *GOLGA3* | 12 | 30 | 9 | 1.68 | 0.046564 | 0.3116 | 2.27 | 0.0117 | 0.2382 |
| *GABARAP* | 17 | 1 | 1 | 1.67 | 0.0471 | 0.3132 | 3.04 | 0.0012 | 0.0787 |
| *CTD-2545G14.7* | 17 | 1 | 1 | 1.67 | 0.0471 | 0.3132 | 3.04 | 0.0012 | 0.0787 |
| *KIAA1731* | 11 | 14 | 4 | 1.67 | 0.047419 | 0.3145 | 1.80 | 0.0358 | 0.3925 |
| *CENPU* | 4 | 68 | 3 | 1.67 | 0.04777 | 0.3156 | 2.52 | 0.0059 | 0.1741 |
| *CUX2* | 12 | 80 | 12 | 1.67 | 0.047834 | 0.3159 | 2.61 | 0.0045 | 0.1516 |
| *SNTB1* | 8 | 222 | 33 | 1.67 | 0.047961 | 0.3163 | 1.94 | 0.0261 | 0.3472 |
| *CRIP3* | 6 | 5 | 1 | 1.66 | 0.048053 | 0.3165 | 3.48 | 0.0003 | 0.0325 |
| *ABCB5* | 7 | 165 | 29 | 1.66 | 0.048323 | 0.3173 | 2.28 | 0.0114 | 0.2353 |
| *DLEU1* | 13 | 491 | 66 | 1.66 | 0.048469 | 0.3173 | 2.23 | 0.0127 | 0.2497 |
| *BTBD11* | 12 | 292 | 35 | 1.66 | 0.048838 | 0.3189 | 1.69 | 0.0455 | 0.4264 |
| *SYT4* | 18 | 4 | 2 | 1.65 | 0.04976 | 0.3223 | 1.68 | 0.0464 | 0.4285 |
| *PHF13* | 1 | 5 | 2 | 1.65 | 0.049965 | 0.3230 | 2.88 | 0.0020 | 0.1000 |
| *KDSR* | 18 | 25 | 5 | 1.65 | 0.049987 | 0.3230 | 2.22 | 0.0133 | 0.2548 |

Abbreviations: SNP, single nucleotide polymorphism; T2D, Type 2 Diabetes

#SNPs: number of SNPs annotated to a specific gene in the data and not excluded based on internal MAGMA quality control; NPARAM: number of SNPs used by MAGMA in the model; Z: Z-statistics for the gene, based on its p-value.

Genes significant after multiple testing correction according to the BH procedure in both BD and BMI are reported in bold.
