# Supplementary material for: Evidence that genes involved in hedgehog signaling are associated with both bipolar disorder and high BMI
Source: Transl Psychiatry. 2019 Nov 21;9:315. doi: 10.1038/s41398-019-0652-x (PMC6872724; doi:10.1038/s41398-019-0652-x)
Supplement: Supplementary file 5 — Supplementary Table 4 [file 41398_2019_652_MOESM5_ESM.docx]

**Supplementary Table 4. Genes associated with bipolar disorder, BMI and T2D in the gene-based analysis conducted with MAGMA**

|  |  |  |  | **Bipolar disorder** | | | **BMI** | | | **Type 2 Disorder** | |  |
| --- | --- | --- | --- | --- | --- | --- | --- | --- | --- | --- | --- | --- |
| **Gene** | **Chr** | **# SNPS** | **NPARAM** | **Z** | **p** | **adj p (BH)** | **Z** | **p** | **adj p (BH)** | **Z** | **p** | **adj p (BH)** |
| ***TAOK2*** | **16** | **11** | **1** | **3.37** | **0.00038** | **0.0200** | **4.28** | **9.4E-06** | **0.0013** | **3.50** | **0.0002** | **0.0299** |
| ***STAB1*** | **3** | **14** | **4** | **4.08** | **2.27E-05** | **0.0034** | **3.20** | **0.0007** | **0.0292** | **3.37** | **0.0004** | **0.0424** |
| ***TMEM219*** | **16** | **3** | **1** | **3.21** | **0.000667** | **0.0286** | **4.06** | **2.4E-05** | **0.0027** | **3.30** | **0.0005** | **0.0491** |
| *NRXN3* | 14 | 1344 | 130 | 1.91 | 0.028022 | 0.2414 | 5.83 | 2.8E-09 | 1.04E-06 | 4.43 | 4.8E-06 | 0.0022 |
| *PEAK1* | 15 | 109 | 10 | 1.88 | 0.029806 | 0.2490 | 1.98 | 0.0241 | 0.2849 | 4.35 | 6.7E-06 | 0.0027 |
| *ARL15* | 5 | 437 | 57 | 1.73 | 0.041855 | 0.2956 | 1.67 | 0.0473 | 0.3936 | 4.08 | 2.2E-05 | 0.0061 |
| *DOC2A* | 16 | 9 | 1 | 1.81 | 0.035168 | 0.2697 | 4.36 | 6.5E-06 | 0.0009 | 3.63 | 0.0001 | 0.0238 |
| *RTN4* | 2 | 126 | 16 | 2.24 | 0.012692 | 0.1592 | 1.92 | 0.0273 | 0.3030 | 3.62 | 0.0001 | 0.0240 |
| *GIPR* | 19 | 3 | 2 | 2.44 | 0.007413 | 0.1185 | 6.91 | 2.4E-12 | 2.5E-09 | 3.53 | 0.0002 | 0.0280 |
| *INO80E* | 16 | 4 | 1 | 2.89 | 0.001933 | 0.0539 | 4.34 | 7.0E-06 | 0.0010 | 3.50 | 0.0002 | 0.0299 |
| *UBE3C* | 7 | 90 | 9 | 2.90 | 0.00184 | 0.0528 | 3.32 | 0.0004 | 0.0219 | 3.42 | 0.0003 | 0.0374 |
| *PTPRN2* | 7 | 671 | 125 | 1.80 | 0.035902 | 0.2718 | 2.28 | 0.0113 | 0.1825 | 3.37 | 0.0004 | 0.0426 |
| *ZNF839* | 14 | 12 | 4 | 2.62 | 0.00444 | 0.0875 | 1.69 | 0.0458 | 0.3848 | 3.36 | 0.0004 | 0.0430 |
| *OASL* | 12 | 13 | 5 | 2.36 | 0.009118 | 0.1332 | 1.76 | 0.0392 | 0.3629 | 3.26 | 0.0006 | 0.0537 |
| *PIGU* | 20 | 43 | 5 | 3.95 | 3.96E-05 | 0.0049 | 2.55 | 0.0054 | 0.1136 | 3.04 | 0.0012 | 0.0787 |
| *CDKN2C* | 1 | 3 | 1 | 4.30 | 8.71E-06 | 0.0017 | 2.86 | 0.0021 | 0.0619 | 2.98 | 0.0014 | 0.0864 |
| *SNX19* | 11 | 81 | 7 | 1.68 | 0.046276 | 0.3104 | 1.66 | 0.0487 | 0.4002 | 2.93 | 0.0017 | 0.0907 |
| *GSS* | 20 | 11 | 3 | 3.38 | 0.000363 | 0.0196 | 2.38 | 0.0087 | 0.1530 | 2.89 | 0.0019 | 0.0985 |
| *GGT7* | 20 | 8 | 2 | 4.22 | 1.24E-05 | 0.0021 | 3.46 | 0.0003 | 0.0154 | 2.89 | 0.0019 | 0.0987 |
| *PHF13* | 1 | 5 | 2 | 1.65 | 0.049965 | 0.3230 | 2.51 | 0.0060 | 0.1222 | 2.88 | 0.0020 | 0.1000 |
| *TP53INP2* | 20 | 4 | 1 | 3.20 | 0.000676 | 0.0289 | 2.97 | 0.0015 | 0.0485 | 2.82 | 0.0024 | 0.1123 |
| *RAB7L1* | 1 | 12 | 3 | 2.00 | 0.022623 | 0.2160 | 3.93 | 4.2E-05 | 0.0042 | 2.82 | 0.0024 | 0.1128 |
| *RPGRIP1L* | 16 | 67 | 8 | 3.11 | 0.000933 | 0.0347 | 3.50 | 0.0002 | 0.0139 | 2.74 | 0.0030 | 0.1271 |
| *NCOA6* | 20 | 42 | 4 | 4.27 | 9.63E-06 | 0.0018 | 3.27 | 0.0005 | 0.0245 | 2.67 | 0.0038 | 0.1392 |
| *MAP1LC3A* | 20 | 2 | 1 | 4.64 | 1.75E-06 | 0.0008 | 2.80 | 0.0026 | 0.0697 | 2.66 | 0.0039 | 0.1401 |
| *PAGR1* | 16 | 4 | 1 | 1.70 | 0.04463 | 0.3049 | 2.52 | 0.0058 | 0.1198 | 2.66 | 0.0039 | 0.1408 |
| *CUX2* | 12 | 80 | 12 | 1.67 | 0.047834 | 0.3159 | 2.32 | 0.0101 | 0.1685 | 2.61 | 0.0045 | 0.1516 |
| *RGS17* | 6 | 130 | 15 | 2.65 | 0.004072 | 0.0833 | 3.62 | 0.0001 | 0.0101 | 2.60 | 0.0046 | 0.1528 |
| *NT5DC2* | 3 | 5 | 1 | 3.93 | 4.24E-05 | 0.0051 | 2.63 | 0.0043 | 0.0983 | 2.57 | 0.0050 | 0.1620 |
| *FAF1* | 1 | 138 | 16 | 4.08 | 2.24E-05 | 0.0034 | 2.20 | 0.0139 | 0.2033 | 2.53 | 0.0057 | 0.1722 |
| *WBP1L* | 10 | 63 | 10 | 2.63 | 0.004251 | 0.0854 | 2.64 | 0.0041 | 0.0955 | 2.52 | 0.0059 | 0.1741 |
| *MVP* | 16 | 9 | 1 | 1.88 | 0.029732 | 0.2488 | 2.86 | 0.0021 | 0.0629 | 2.52 | 0.0059 | 0.1746 |
| *PPIL2* | 22 | 38 | 5 | 2.07 | 0.019154 | 0.1976 | 2.08 | 0.0190 | 0.2480 | 2.48 | 0.0066 | 0.1893 |
| *BLMH* | 17 | 20 | 5 | 1.89 | 0.029334 | 0.2473 | 1.95 | 0.0256 | 0.2922 | 2.44 | 0.0073 | 0.1966 |
| *HSD11B2* | 16 | 1 | 1 | 3.14 | 0.000859 | 0.0332 | 1.97 | 0.0245 | 0.2865 | 2.41 | 0.0079 | 0.2030 |
| *MRAS* | 3 | 40 | 4 | 2.26 | 0.01194 | 0.1546 | 3.59 | 0.0002 | 0.0112 | 2.38 | 0.0086 | 0.2080 |
| *JADE2* | 5 | 40 | 10 | 3.90 | 4.90E-05 | 0.0055 | 2.78 | 0.0027 | 0.0724 | 2.33 | 0.0098 | 0.2184 |
| *ALDOA* | 16 | 3 | 2 | 1.98 | 0.023737 | 0.2205 | 2.98 | 0.0014 | 0.0477 | 2.31 | 0.0105 | 0.2281 |
| *GOLGA3* | 12 | 30 | 9 | 1.68 | 0.046564 | 0.3116 | 2.41 | 0.0081 | 0.1461 | 2.27 | 0.0117 | 0.2382 |
| *ATP6V0D1* | 16 | 12 | 3 | 3.10 | 0.000959 | 0.0354 | 1.90 | 0.0288 | 0.3116 | 2.26 | 0.0119 | 0.2401 |
| *DRD2* | 11 | 69 | 10 | 1.70 | 0.044779 | 0.3053 | 2.97 | 0.0015 | 0.0483 | 2.26 | 0.0120 | 0.2406 |
| *SMIM4* | 3 | 18 | 2 | 4.74 | 1.09E-06 | 0.0005 | 1.96 | 0.0253 | 0.2898 | 2.23 | 0.0128 | 0.2501 |
| *RLTPR* | 16 | 2 | 1 | 2.74 | 0.003106 | 0.0706 | 2.54 | 0.0056 | 0.1161 | 2.21 | 0.0134 | 0.2556 |
| *ITGAL* | 16 | 24 | 9 | 2.04 | 0.020839 | 0.2068 | 2.57 | 0.0051 | 0.1097 | 2.19 | 0.0143 | 0.2636 |
| *RORB* | 9 | 161 | 22 | 2.84 | 0.002254 | 0.0587 | 1.74 | 0.0407 | 0.3693 | 2.18 | 0.0146 | 0.2669 |
| *MST1R* | 3 | 6 | 2 | 2.23 | 0.012971 | 0.1601 | 4.01 | 3.0E-05 | 0.0032 | 2.17 | 0.0149 | 0.2696 |
| *PLCG1* | 20 | 16 | 4 | 1.74 | 0.04135 | 0.2939 | 2.44 | 0.0073 | 0.1378 | 2.17 | 0.0151 | 0.2696 |
| *ZDHHC1* | 16 | 6 | 2 | 2.72 | 0.003261 | 0.0729 | 1.94 | 0.0262 | 0.2965 | 2.16 | 0.0155 | 0.2718 |
| *DNAJC11* | 1 | 30 | 5 | 1.83 | 0.033923 | 0.2661 | 2.53 | 0.0058 | 0.1189 | 2.15 | 0.0157 | 0.2732 |
| *CENPT* | 16 | 5 | 2 | 4.01 | 3.07E-05 | 0.0041 | 3.28 | 0.0005 | 0.0241 | 2.15 | 0.0160 | 0.2768 |
| *PRRT2* | 16 | 2 | 1 | 1.80 | 0.036219 | 0.2729 | 2.68 | 0.0037 | 0.0891 | 2.14 | 0.0161 | 0.2784 |
| *PBRM1* | 3 | 52 | 3 | 4.84 | 6.38E-07 | 0.0004 | 2.22 | 0.0131 | 0.1982 | 2.12 | 0.0169 | 0.2851 |
| *LRRC36* | 16 | 19 | 3 | 2.69 | 0.003594 | 0.0771 | 1.80 | 0.0359 | 0.3447 | 2.11 | 0.0175 | 0.2937 |
| *RANBP1* | 22 | 4 | 2 | 1.69 | 0.045461 | 0.3073 | 1.70 | 0.0449 | 0.3819 | 2.09 | 0.0184 | 0.3003 |
| *QKI* | 6 | 126 | 10 | 2.43 | 0.007572 | 0.1196 | 1.96 | 0.0250 | 0.2885 | 2.08 | 0.0186 | 0.3016 |
| *RPS6KA5* | 14 | 96 | 9 | 2.29 | 0.01101 | 0.1487 | 2.46 | 0.0070 | 0.1351 | 2.07 | 0.0191 | 0.3047 |
| *GNL3* | 3 | 7 | 2 | 4.84 | 6.59E-07 | 0.0004 | 2.45 | 0.0071 | 0.1359 | 2.06 | 0.0195 | 0.3069 |
| *PHF7* | 3 | 4 | 2 | 3.78 | 7.98E-05 | 0.0075 | 1.72 | 0.0428 | 0.3761 | 2.05 | 0.0201 | 0.3109 |
| *CTD-2616J11.11* | 19 | 6 | 2 | 1.86 | 0.031374 | 0.2569 | 2.44 | 0.0073 | 0.1375 | 2.05 | 0.0203 | 0.3125 |
| *GMIP* | 19 | 4 | 2 | 2.43 | 0.007492 | 0.1189 | 2.02 | 0.0216 | 0.2672 | 2.01 | 0.0220 | 0.3261 |
| *NEK4* | 3 | 13 | 3 | 4.96 | 3.52E-07 | 0.0003 | 2.19 | 0.0142 | 0.2043 | 2.01 | 0.0222 | 0.3265 |
| *SIGLECL1* | 19 | 22 | 4 | 2.52 | 0.005848 | 0.1030 | 1.88 | 0.0300 | 0.3182 | 1.99 | 0.0234 | 0.3339 |
| *NKAPL* | 6 | 4 | 2 | 3.60 | 0.000161 | 0.0113 | 1.93 | 0.0268 | 0.2998 | 1.97 | 0.0243 | 0.3370 |
| *RANBP10* | 16 | 10 | 3 | 3.43 | 0.000306 | 0.0173 | 2.63 | 0.0042 | 0.0969 | 1.96 | 0.0248 | 0.3399 |
| *SPCS1* | 3 | 1 | 1 | 4.96 | 3.52E-07 | 0.0003 | 2.54 | 0.0056 | 0.1162 | 1.94 | 0.0260 | 0.3466 |
| *ITIH1* | 3 | 16 | 2 | 4.65 | 1.69E-06 | 0.0008 | 3.44 | 0.0003 | 0.0166 | 1.94 | 0.0265 | 0.3483 |
| *AMN* | 14 | 4 | 2 | 2.53 | 0.005624 | 0.1008 | 1.94 | 0.0260 | 0.2960 | 1.93 | 0.0271 | 0.3514 |
| *GLT8D1* | 3 | 4 | 1 | 4.48 | 3.66E-06 | 0.0010 | 1.99 | 0.0232 | 0.2779 | 1.92 | 0.0273 | 0.3529 |
| *KCTD13* | 16 | 10 | 2 | 2.90 | 0.00188 | 0.0536 | 3.55 | 0.0002 | 0.0123 | 1.92 | 0.0273 | 0.3529 |
| *MSRA* | 8 | 545 | 43 | 3.13 | 0.000866 | 0.0332 | 3.37 | 0.0004 | 0.0190 | 1.90 | 0.0285 | 0.3595 |
| *XRCC3* | 14 | 11 | 3 | 2.08 | 0.018784 | 0.1956 | 3.29 | 0.0005 | 0.0237 | 1.90 | 0.0285 | 0.3595 |
| *ATXN2L* | 16 | 5 | 1 | 2.02 | 0.021643 | 0.2106 | 7.29 | 1.5E-13 | 2.4E-10 | 1.89 | 0.0296 | 0.3656 |
| *SLC4A10* | 2 | 253 | 19 | 3.58 | 0.000171 | 0.0117 | 1.80 | 0.0359 | 0.3447 | 1.88 | 0.0298 | 0.3664 |
| *SPHKAP* | 2 | 154 | 18 | 3.16 | 0.0008 | 0.0319 | 3.39 | 0.0003 | 0.0183 | 1.85 | 0.0325 | 0.3808 |
| *MAP2K5* | 15 | 191 | 17 | 1.83 | 0.033925 | 0.2661 | 6.47 | 5.0E-11 | 3.0E-08 | 1.82 | 0.0345 | 0.3864 |
| *SFMBT1* | 3 | 60 | 7 | 4.49 | 3.54E-06 | 0.0010 | 1.72 | 0.0429 | 0.3761 | 1.81 | 0.0348 | 0.3879 |
| *PSMB10* | 16 | 2 | 1 | 3.34 | 0.000424 | 0.0215 | 1.74 | 0.0410 | 0.3709 | 1.78 | 0.0378 | 0.4021 |
| *CTC-479C5.12* | 16 | 2 | 1 | 3.31 | 0.00046 | 0.0227 | 1.73 | 0.0419 | 0.3735 | 1.77 | 0.0381 | 0.4022 |
| *SLC4A1* | 17 | 9 | 4 | 4.59 | 2.26E-06 | 0.0008 | 2.13 | 0.0166 | 0.2268 | 1.75 | 0.0403 | 0.4083 |
| *HS6ST3* | 13 | 518 | 43 | 3.17 | 0.000751 | 0.0309 | 4.96 | 3.5E-07 | 0.0001 | 1.75 | 0.0404 | 0.4089 |
| *LMBR1L* | 12 | 5 | 1 | 3.62 | 0.000147 | 0.0107 | 3.41 | 0.0003 | 0.0173 | 1.73 | 0.0417 | 0.4147 |
| *TUBA1C* | 12 | 20 | 5 | 1.74 | 0.041014 | 0.2926 | 3.59 | 0.0002 | 0.0112 | 1.72 | 0.0425 | 0.4193 |
| *ACHE* | 7 | 1 | 1 | 3.47 | 0.000257 | 0.0152 | 1.71 | 0.0440 | 0.3793 | 1.72 | 0.0430 | 0.4214 |
| *MAPK1* | 22 | 66 | 7 | 4.93 | 4.11E-07 | 0.0003 | 3.00 | 0.0014 | 0.0465 | 1.71 | 0.0433 | 0.4223 |
| *ETFB* | 19 | 13 | 3 | 2.44 | 0.007425 | 0.1185 | 2.69 | 0.0036 | 0.0870 | 1.71 | 0.0434 | 0.4223 |
| *PARD3* | 10 | 565 | 40 | 1.83 | 0.033618 | 0.2654 | 2.74 | 0.0030 | 0.0784 | 1.71 | 0.0434 | 0.4223 |
| *ZNF101* | 19 | 7 | 2 | 3.24 | 0.000597 | 0.0269 | 2.90 | 0.0018 | 0.0560 | 1.70 | 0.0442 | 0.4247 |
| *ITIH3* | 3 | 7 | 2 | 5.51 | 1.77E-08 | 5.0E-05 | 3.37 | 0.0004 | 0.0190 | 1.69 | 0.0455 | 0.4264 |
| *UBAP2* | 9 | 72 | 8 | 3.71 | 0.000102 | 0.0087 | 3.39 | 0.0004 | 0.0184 | 1.68 | 0.0464 | 0.4285 |
| *TUBA1A* | 12 | 1 | 1 | 2.79 | 0.002605 | 0.0640 | 3.07 | 0.0011 | 0.0398 | 1.67 | 0.0470 | 0.4313 |
| *TUBA1B* | 12 | 2 | 1 | 2.53 | 0.005766 | 0.1021 | 2.96 | 0.0015 | 0.0493 | 1.67 | 0.0473 | 0.4327 |
| *ANKRD36* | 2 | 7 | 2 | 2.46 | 0.006868 | 0.1128 | 2.41 | 0.0079 | 0.1447 | 1.66 | 0.0487 | 0.4356 |
| *DDX19A* | 16 | 5 | 1 | 2.67 | 0.003844 | 0.0810 | 2.62 | 0.0043 | 0.0990 | 1.65 | 0.0495 | 0.4370 |

Abbreviations: BMI, body mass index; SNP, single nucleotide polymorphism, T2D, type 2 diabetes.

#SNPs: number of SNPs annotated to a specific gene in the data and not excluded based on internal MAGMA quality control; NPARAM: number of SNPs used by MAGMA in the model; Z: the Z-statistics for the gene, based on its p-value.

Genes significant after multiple testing correction according to the BH procedure in both BD and BMI are reported in bold.
