# Supplementary material for: Evidence that genes involved in hedgehog signaling are associated with both bipolar disorder and high BMI
Source: Transl Psychiatry. 2019 Nov 21;9:315. doi: 10.1038/s41398-019-0652-x (PMC6872724; doi:10.1038/s41398-019-0652-x)
Supplement: Supplementary file 6 — Supplementary Table 5 [file 41398_2019_652_MOESM6_ESM.docx]

**Supplementary Table 5. Significantly enriched pathways for genes commonly associated with bipolar disorder and BMI**

| **Gene Set** | **Description** | **Size** | **Observed** | **Enrichment ratio** | **P** | **FDR** | **Included genes** |
| --- | --- | --- | --- | --- | --- | --- | --- |
| **R-HSA-109582** | **Hemostasis** | **620** | **38** | **2.25** | **2.1E-06** | **0.004** | ***AK3, AKAP10, ALDOA, ATP2A1, ATP2A2, ATP2B2, CBX5, CDC37L1, CSK, DAGLA, DGKG, DGK1, ECM1, ITGAL, ITGB3, ITIH3, ITIH4, ITPR3, KIF5A, KLC1, MAPK1, MAPK3, MFN2, NEF2, OLA1, PDE3B, PLCG1, PPIL2, PRKACA, SH2B1, SLC7A6, SLC8A1, TUBA1A, TUBA1B, TUBA1C, TUBA4A, VCL, ZFPM2*** |
| **R-HSA-5358351** | **Signaling by Hedgehog** | **147** | **14** | **3.50** | **4.8E-05** | **0.020** | ***ADCY9, DERL2, IFT172, IFT57, PRKACA, PSMA5, PSMB10, RPGRIP1L, SUFU, TUBA1A, TUBA1B, TUBA1C, TUBA4A, ULK3*** |
| **R-HSA-373760** | **L1CAM interactions** | **117** | **12** | **3.76** | **8.1E-05** | **0.020** | ***ITGB3, KCNQ2, MAP2K1, MAPK1, MAPK3, NCAM1, RPS6KA5, SHTN1, TUBA1A, TUBA1B, TUBA1C, TUBA4A*** |
| **R-HSA-6802952** | **Signaling by BRAF and RAF fusions** | **60** | **8** | **4.89** | **0.0002** | **0.030** | ***CSK, ITGB3, MAP2K1, MAPK1, MAPK3, MARK3, QKI, VCL*** |
| R-HSA-445144 | Signal transduction by L1 | 21 | 5 | 8.72 | 2.0E-04 | 0.030 | *ITGB3, MAP2K1, MAPK1, MAPK3, NCAM1* |
| R-HSA-6802948 | Signaling by high-kinase activity BRAF mutants | 36 | 7 | 7.13 | 4.0E-05 | 0.019 | *CSK, ITGB3, MAP2K1, MAPK1, MAPK3, MARK3, VCL* |
| R-HSA-5674135 | MAP2K and MAPK activation | 40 | 7 | 6.41 | 9.0E-05 | 0.020 | *CSK, ITGB3, MAP2K1, MAPK1, MAPK3, MARK3, VCL* |
| R-HSA-6802946 | Signaling by moderate kinase activity BRAF mutants | 40 | 7 | 6.41 | 9.0E-05 | 0.020 | *CSK, ITGB3, MAP2K1, MAPK1, MAPK3, MARK3, VCL* |
| R-HSA-6802955 | Paradoxical activation of RAF signaling by kinase inactive BRAF | 40 | 7 | 6.41 | 9.0E-05 | 0.020 | *CSK, ITGB3, MAP2K1, MAPK1, MAPK3, MARK3, VCL* |
| R-HSA-437239 | Recycling pathway of L1 | 45 | 7 | 5.70 | 2.0E-04 | 0.030 | *MAPK1, RPS6KA5, SHTN1, TUBA1A, TUBA1B, TUBA1C, TUB4A* |
| R-HSA-5610787 | Hedgehog 'off' state | 111 | 12 | 3.96 | 4.8E-05 | 0.020 | *ADCY9, IFT172, IFT57, PRKACA, PSMA5, PSMB10, RPGRIP1L, SUFU, TUBA1A, TUBA1B, TUBA1C, TUBA4A* |
| R-HSA-983231 | Factors involved in megakaryocyte development and platelet production | 166 | 14 | 3.09 | 1.8E-04 | 0.030 | *AK3, AKAP10, CBX5, KIF5A, KLC1, MFN2, NFE2, PRKACA, SH2B1, TUBA1A, TUBA1B, TUBA1C, TUBA4A, ZFPM2* |

Abbreviations: FDR, false discovery rate

Reactome pathways significantly enriched for genes commonly associated with bipolar disorder and BMI were obtained using WebGestalt. Pathways included in the weighted set, i.e. the minimum subset of gene sets that can cover all the genes from the enriched sets, are indicated in bold.
