# Supplementary material for: Evidence that genes involved in hedgehog signaling are associated with both bipolar disorder and high BMI
Source: Transl Psychiatry. 2019 Nov 21;9:315. doi: 10.1038/s41398-019-0652-x (PMC6872724; doi:10.1038/s41398-019-0652-x)
Supplement: Supplementary file 7 — Supplementary Table 6 [file 41398_2019_652_MOESM7_ESM.docx]

**Supplementary Table 6. Significant eQTLs in adipose tissue and different brain regions in GTEx among SNPs associated with BD and metabolic phenotypes**

| **SNP** | **Gene** | **Adipose - Subcutaneous** | **Adipose - Visceral (Omentum)** | **Brain Amygdala** | **Brain - Anterior cingulate cortex (BA24)** | **Brain - Caudate** | **Brain - Cerebellar Hemisphere** | **Brain - Cerebellum** | **Brain - Cortex** | **Brain - Frontal Cortex (BA9)** | **Brain - Hippocampus** | **Brain - Hypothalamus** | **Brain - Nucleus accumbens** | **Brain - Putamen** |
| --- | --- | --- | --- | --- | --- | --- | --- | --- | --- | --- | --- | --- | --- | --- |
| **SNPs associated with BD and BMI** | | | | | | | | | | | | | | |
| rs3888190 | Intergenic | *CDC37P1, SULT1A2, RP11-1348G14.4, TUFM, SH2B1, EIF3C, NPIPB7, ATXN2L* | *SULT1A2, RP11-1348G14.4, TUFM, SH2B1* | *TUFM, RP11-1348G14.4, SULT1A2* | *NPIPB7, SULT1A2* | *TUFM, SULT1A2, SULT1A1* | *NPIPB7, RP11-1348G14.4, RP11-22P6.2, NPIPB9* | *SH2B1, NPIPB7, RP11-22P6.2, NPIPB9* | *NPIPB7, SULT1A2, SULT1A1* | *-* | *TUFM, SULT1A2, SULT1A1* | *NPIPB7, RP11-1348G14.4* | *TUFM, NPIPB7, RP11-1348G14.4* | *TUFM, SULT1A2, NPIPB7* |
| rs7498665 | *NPIPL1-SH2B1* | *CDC37P1, SULT1A2, RP11-1348G14.4, TUFM, SH2B1, EIF3C, NPIPB7* | *SULT1A2, RP11-1348G14.4, TUFM, SH2B1* | *TUFM, RP11-1348G14.4, SULT1A2* | *SULT1A2, NPIPB7* | *TUFM, SULT1A2, SULT2A2* | *NPIPB7, RP11-1348G14.4, RP11-22P6.2, SULT1A2* | *NPIPB7, SH2B1, RP11-22P6.2, NPIPB9* | *NPIPB7, SULT1A1, SULT1A2* | *SULT1A2* | *TUFM, SULT1A2, SULT1A1* | *NPIPB7, RP11-1348G14.4* | *TUFM, NPIPB7, RP11-1348G14.4, SULT1A2* | *TUFM, SULT1A2, NPIPB7* |
| rs8055982 | *NPIPL1-SH2B1* | *CDC37P1, SULT1A2, RP11-1348G14.4, TUFM, SH2B1, EIF3C, NPIPB7, ATXN2L* | *SULT1A2, RP11-1348G14.4, TUFM, SH2B1* | *TUFM, RP11-1348G14.4, SULT1A2* | *SULT1A2, NPIPB7* | *TUFM, SULT1A2, SULT1A1* | *NPIPB7, RP11-1348G14.4, RP11-22P6.2, NPIPB9* | *NPIPB7, SH2B1, RP11-22P6.2, NPIPB9* | *NPIPB7, SULT1A1, SULT1A2* | *-* | *TUFM, SULT1A2, SULT1A1* | *NPIPB7, RP11-1348G14.4* | *TUFM, NPIPB7, RP11-1348G14.4* | *TUFM, SULT1A2, NPIPB7* |
| rs4788102 | *NPIPL1-SH2B1* | *CDC37P1, SULT1A2, RP11-1348G14.4, TUFM, SH2B1, EIF3C, NPIPB7, ATXN2L* | *SULT1A2, RP11-1348G14.4, TUFM, SH2B1* | *TUFM, RP11-1348G14.4, SULT1A2* | *NPIPB7, SULT1A2* | *TUFM, SULT1A2, SULT1A1* | *NPIPB7, RP11-1348G14.4, RP11-22P6.2, NPIPB9* | *SH2B1, NPIPB7, RP11-22P6.2, NPIPB9* | *NPIPB7, SULT1A2, SULT1A1* | *-* | *TUFM, SULT1A2, SULT1A1* | *NPIPB7, RP11-1348G14.4* | *TUFM, NPIPB7, RP11-1348G14.4* | *TUFM, SULT1A2, NPIPB7* |
| rs7359397 | *SH2B1-NPIPL1* | *SULT1A2, CDC37P1, RP11-1348G14, TUFM, EIF3C, NPIPB7, SH2B1, NFATC2IP* | *SULT1A2, RP11-1348G14.4, TUFM, SH2B1* | *TUFM, RP11-1348G14.4, SULT1A2* | *SULT1A2* | *TUFM, SULT1A2* | *NPIPB7, RP11-1348G14.4, RP11-22P6.2* | *SH2B1, NPIPB7, RP11-22P6.2* | *NPIPB7, SULT1A2, SULT1A1* | *-* | *TUFM, SULT1A2* | *NPIPB7, RP11-1348G14.4* | *TUFM, NPIPB7, EIF3C* | *TUFM, SULT1A2, NPIPB7* |
| rs12443881 | *NPIPL1-ATXN2L* | *SULT1A2, CDC37P1, RP11-1348G14, TUFM, EIF3C, NPIPB7, SH2B1, NFATC2IP* | *SULT1A2, RP11-1348G14.4, TUFM, SH2B1* | *TUFM, RP11-1348G14.4, SULT1A2* | *SULT1A2* | *TUFM, SULT1A2* | *NPIPB7, RP11-1348G14.4, RP11-22P6.2* | *SH2B1, NPIPB7, RP11-22P6.* | *NPIPB7, SULT1A2, SULT1A1* | *-* | *TUFM, SULT1A2* | *NPIPB7, RP11-1348G14.4* | *TUFM, NPIPB7, RP11-1348G14.4* | *TUFM, SULT1A2, NPIPB7* |
| rs7187776 | *NPIPL1-ATXN2L* | *SULT1A2, RP11-1348G14.4, TUFM, CDC37P1, SH2B1, EIF3C, NPIPB7, ATXN2L* | *NFATC2IP, SULT1A2, RP11-1348G14.4, TUFM, SH2B1* | *TUFM, RP11-1348G14.4* | *SULT1A2, NPIPB7* | *TUFM, SULT1A2* | *NPIPB7, RP11-1348G14.4, RP11-22P6.2* | *NPIPB7, SH2B1, RP11-22P6.2, RP11-1348G14.4* | *NPIPB7, SULT1A2, SULT1A1* | *TUFM* | *TUFM* | *NPIPB7, RP11-1348G14.4* | *TUFM, RP11-1348G14.4* | *TUFM, SULT1A2* |
| rs8049439 | *NPIPL1-ATXN2L* | *SULT1A2, RP11-1348G14.4, CDC37P1, TUFM, SH2B1, EIF3C, NPIPB7, ATXN2L, NFATC2IP* | *SULT1A2, RP11-1348G14.4, TUFM, SH2B1* | *TUFM, RP11-1348G14.4* | *SULT1A2, NPIPB7* | *TUFM, SULT1A2* | *NPIPB7, RP11-1348G14.4, RP11-22P6.2* | *NPIPB7, SH2B1, RP11-22P6.2, RP11-1348G14.4* | *NPIPB7, SULT1A2, SULT1A1* | *TUFM* | *TUFM* | *NPIPB7, RP11-1348G14.4* | *TUFM, RP11-1348G14.4* | *TUFM, SULT1A2* |
| rs8055138 | *NPIPL1-ATP2A1-AS1* | *CDC37P1, SULT1A2, RP11-1348G14.4, TUFM, SH2B1, EIF3C, NPIPB7, ATXN2L* | *SULT1A2, RP11-1348G14.4, TUFM, SH2B1* | *TUFM, RP11-1348G14.4, SULT1A2* | *NPIPB7, SULT1A2* | *TUFM, SULT1A2, SULT1A1* | *NPIPB7, RP11-1348G14.4, RP11-22P6.2, NPIPB9* | *SH2B1, NPIPB7, RP11-22P6.2, NPIPB9* | *NPIPB7, SULT1A2, SULT1A1* | *-* | *TUFM, SULT1A2, SULT1A1* | *NPIPB7, RP11-1348G14.4* | *TUFM, NPIPB7, RP11-1348G14.4* | *TUFM, SULT1A2, NPIPB7* |
| rs4788084 | *NPIPL1* | *CDC37P1, SULT1A2, RP11-1348G14.4, TUFM, SH2B1, EIF3C, NPIPB7* | *SULT1A2, RP11-1348G14.4, TUFM, SH2B1, NPIPB7* | *TUFM, RP11-1348G14.4, SULT1A2* | *NPIPB7, SULT1A2* | *TUFM, SULT1A2, SULT1A1, NPIPB7* | *NPIPB7, RP11-1348G14.4, NUPR1* | *SH2B1, NPIPB7, RP11-22P6.2, SULT1A1* | *NPIPB7, SULT1A2, SULT1A1* | *SULT1A2, NPIPB7* | *TUFM, SULT1A2, SULT1A1, NPIPB7* | *NPIPB7, RP11-1348G14.4, SULT1A1, SULT1A2* | *TUFM, NPIPB7, RP11-1348G14.4, SULT1A2* | *TUFM, SULT1A2, NPIPB7* |
| rs12928404 | *NPIPL1-ATXN2L* | *SULT1A2, CDC37P1, TUFM, RP11-1348G14.4, SH2B1, EIF3C, NFATC2IP, NPIPB7* | *SULT1A2, RP11-1348G14.4, TUFM, SH2B1* | *TUFM, RP11-1348G14.4, SULT1A2* | *SULT1A2* | *TUFM, SULT1A2* | *NPIPB7, RP11-1348G14.4, SULT1A2* | *NPIPB7, SH2B1, RP11-22P6.2,* | *NPIPB7, SULT1A2, SULT1A1, TUFM* | *TUFM* | *TUFM,* | *NPIPB7, RP11-1348G14.4* | *TUFM* | *TUFM, SULT1A2* |
|  | | | | | | | | | | | | | | |
| **SNPs associated with BD and T2D** | | | | | | | | | | | | | | |
| rs614288 | Intergenic | - | - | - | - | - | - | *PPM1M* | - | - | - | - | - | - |
| rs164640 | *ALAS1* | *TMEM110* | *ITIH4* | - | - | - | - | *GLYCTK, PPM1M* | - | - | - | - | - | - |
| rs352162 | Intergenic | *ITIH4, TMEM110* | - | - | - | - | - | *GLYCTK, PPM1M* | - | - | *GLYCTK* | - | - | - |

BD, bipolar disorder; BMI, body mass index; eQTL, expression quantitative trait loci, T2D, type 2 diabetes
