# Supplementary figures and images for: Evidence that genes involved in hedgehog signaling are associated with both bipolar disorder and high BMI
Source: Transl Psychiatry. 2019 Nov 21;9:315. doi: 10.1038/s41398-019-0652-x (PMC6872724; doi:10.1038/s41398-019-0652-x)

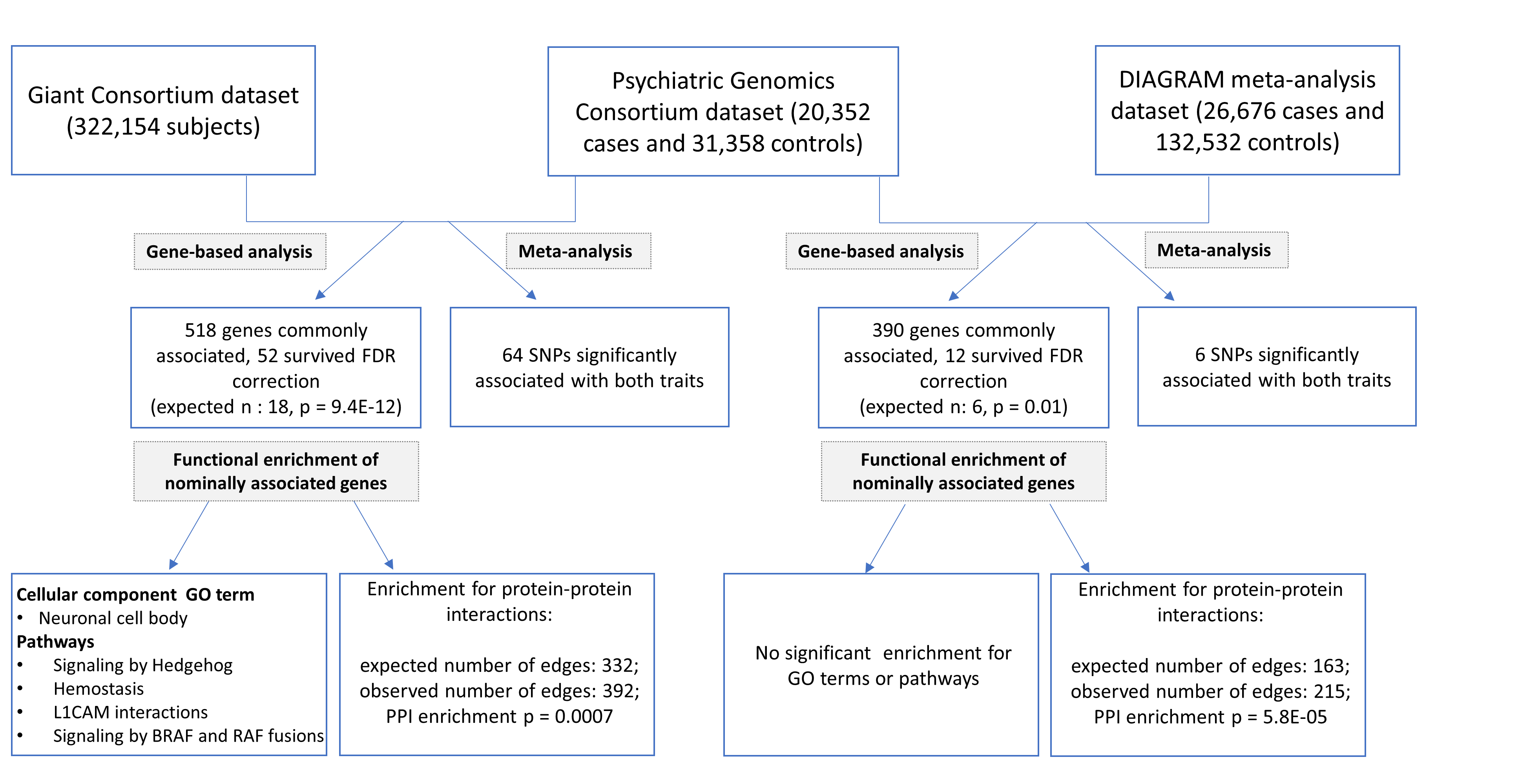

Supplement: Supplementary file 8 — Supplementary Figure 1 [file 41398_2019_652_MOESM8_ESM.tif]

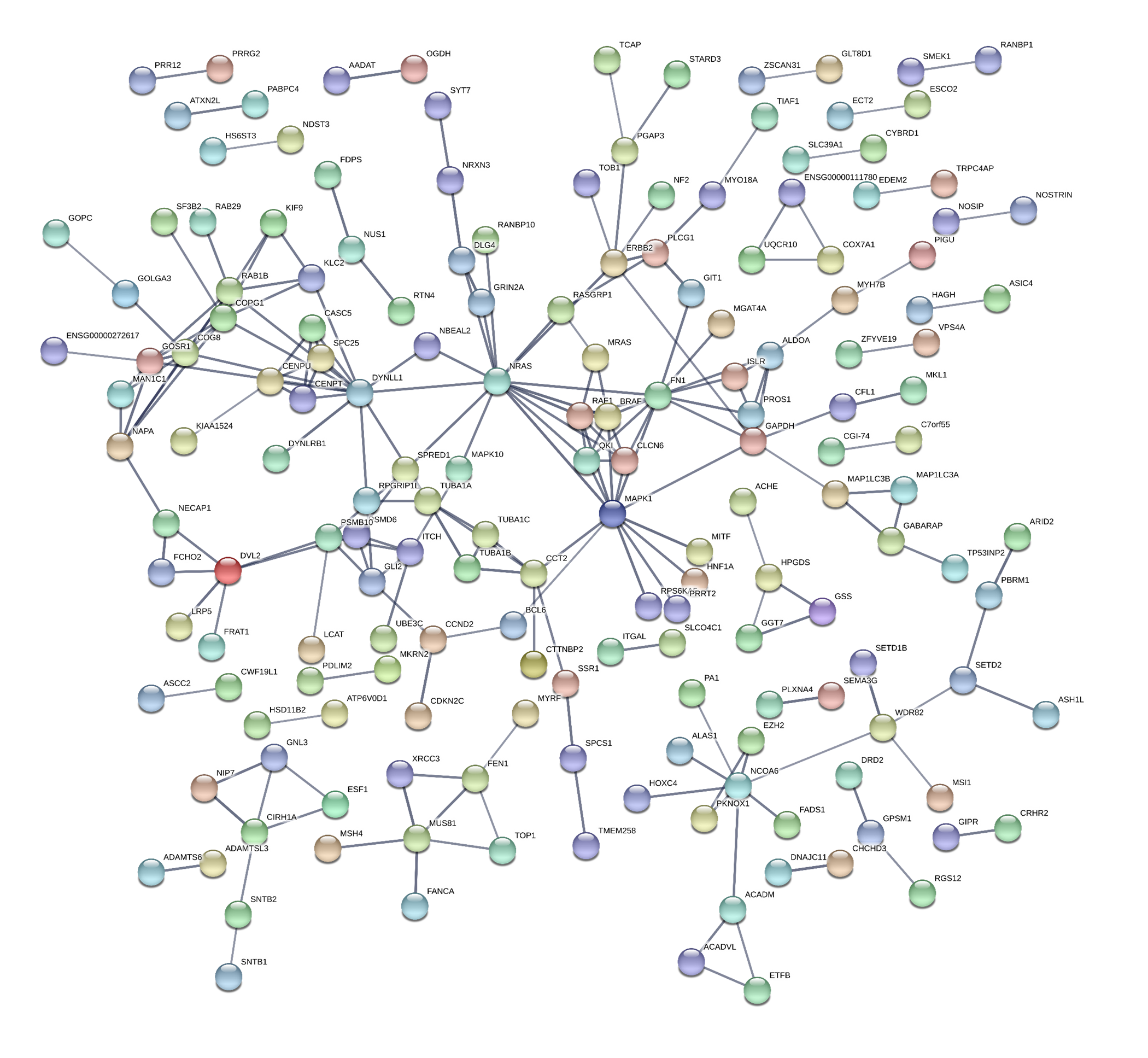

Supplement: Supplementary file 9 — Supplementary Figure 2 [file 41398_2019_652_MOESM9_ESM.tif]
